# Analysis of mononucleotides by tandem mass spectrometry: investigation of fragmentation pathways for phosphate- and ribose-modified nucleotide analogues

**DOI:** 10.1038/s41598-017-09416-6

**Published:** 2017-08-21

**Authors:** Dominika Strzelecka, Sebastian Chmielinski, Sylwia Bednarek, Jacek Jemielity, Joanna Kowalska

**Affiliations:** 1 Division of Biophysics, Institute of Experimental Physics, Faculty of Physics, University of Warsaw, Pasteura 5, 02-093 Warsaw, Poland; 2 Centre of New Technologies, University of Warsaw, Banacha 2c, 02-097 Warsaw, Poland

## Abstract

Synthetic nucleotide and nucleic acid analogues are useful research tools and modern therapeutics. Hence, methods for the rapid and unambiguous identification of mononucleotides derived from organic syntheses or biological materials are of broad interest. Here, we analysed over 150 mononucleotides (mostly nucleoside 5′-mono-, 5′-di-, and 5′-triphosphates) and their structurally related nucleobase-, phosphate-, and ribose-modified analogues by electrospray tandem mass spectrometry (ESI/MS/MS), identifying characteristic fragmentation ions that may be helpful in structure determination. While positive-ion mode yielded fragments derived mainly from nucleobases, negative-ion mode provided insight into the structures of phosphoryl and phosphoribosyl moieties, enabling the determination of structural features such as the number of phosphate groups and the presence of ribose or phosphate substitutions. Based on these data, we proposed fragmentation pathways that were confirmed by experiments with [^18^O]-isotopologues. We demonstrated the utility of ESI(−)/MS/MS in the analysis of structurally related compounds by analysing isomeric and isobaric nucleotides and applying ESI(−)/MS/MS to rapid identification of nucleotide synthesis products. We formulated general rules regarding nucleotide structure–fragmentation pattern relationships and indicating characteristic fragmentation ions for the interpretation of ESI(−)/MS/MS spectra of nucleotides and their analogues. The ESI(−)/MS/MS spectra of all nucleotides are available in an on-line database, msTide, at www.msTide-db.com.

## Introduction

Nucleotides are ubiquitous molecules with multiple biological functions. Consequently, synthetic nucleotide analogues have become increasingly useful research tools and a promising class of therapeutics with applications in anti-viral, anti-cancer, cardiovascular, and other therapies^1^. Structural analysis of nucleotides and their analogues is a key step in understanding the roles of endogenous nucleotides as well as in the development, biochemical analysis, and therapeutic usage of new nucleotide-based tools. Tandem mass spectrometry (MS/MS) is a method used for the identification and quantification of nucleotides and their analogues in biological mixtures. MS/MS has been employed to determine the structures^2, 3^ and cellular fates^4^ of endogenous nucleotides and to quantify blood or tissue levels of therapeutically relevant nucleotide analogues such as AZT oligophosphates^5^, gemcitabine triphosphate^6^, bisphosphonate (clodronate) metabolites^7^, and others^8–12^.

**Figure 1:**
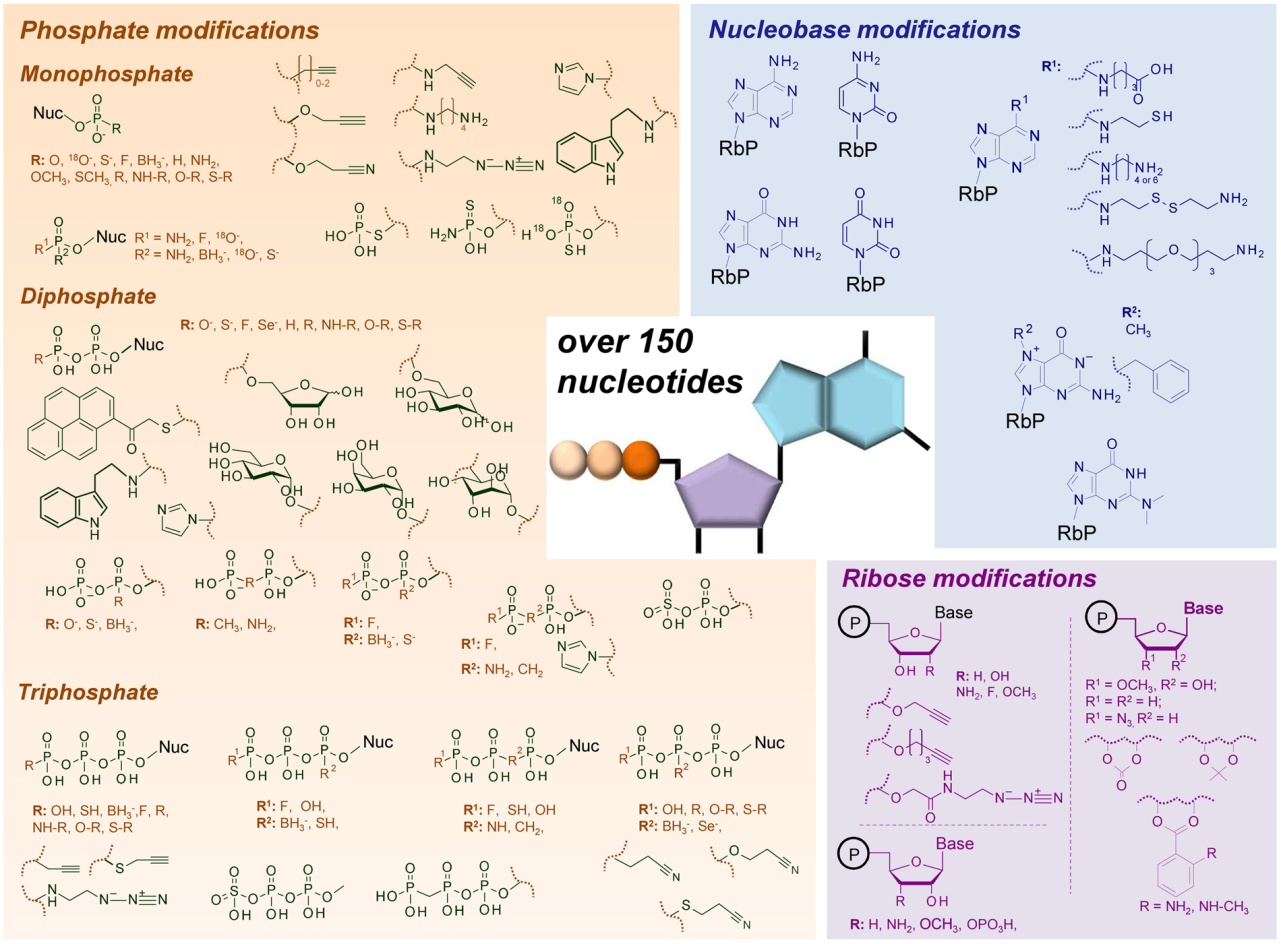
Nucleotides studied in this work—an overview. The nucleotide library contained over 150 compounds, including unmodified nucleotides, nucleotides modified either within the nucleobase, the ribose, or the phosphate moiety, and nucleotides containing various combinations of those modifications. Exact structures of all nucleotides are shown in Table S1. Nuc = 5′-nucleosidyl moiety, RbP = Phosphoribose.

Phosphate- and ribose-modified analogues constitute a nucleotide subgroup with interesting biological properties and applications arising from their reduced susceptibility to enzymatic degradation and amenability to functionalization. This class of nucleotide analogues has been used as enzymatic inhibitors^13, 14^ and labelled probes^15, 16^ and has been useful in several emerging therapeutic applications^7, 14, 17–23^; numerous examples have also been found naturally (Fig. S1)^24–26^. Along with a deeper understanding of their biological impact and an increasing number of applications for phosphate- and ribose-modified nucleotides, methods for studying their structures, *in vivo* occurrences, stabilities, and metabolisms are in high demand, with mass spectrometry being the primary tool. However, little is known regarding the general rules governing the fragmentation of nucleoside oligophosphates and, in particular, their phosphate- and ribose-modified analogues, as previous reports have usually focused on a single molecule type or narrow subgroup of molecules^14, 27, 28^. To fill this gap, we took advantage of our ‘in house’ library of nucleotide analogues to perform a broad and in-depth study on the fragmentation of nucleotides and their synthetic analogues by tandem mass spectrometry. We applied electrospray ionization with a triple quadrupole analyser to obtain fragmentation spectra of over 150 nucleotides and nucleotide analogues. We focused on compounds that contain modifications within the 5′-oligophosphate and ribose moieties, although various nucleobase-modified compounds were also included (Fig. 1). We found that tandem mass spectrometry, especially in the negative-ion mode, can be a valuable source of information on the presence, type, and position of non-isobaric and, in many cases, isobaric and isomeric modifications in nucleotides. The MS/MS spectra are freely available in an on-line database (www.mstide-db.com), which is, to our knowledge, the first database for mononucleotide MS/MS spectra. The results of our study should be relevant to those working on the biological aspects of the structure and function of modified nucleotides as well as in synthetic nucleotide chemistry.

## Results and Discussion

### Nucleotide library and database

We analysed a library of over 150 mononucleotides and their synthetic analogues (Supplementary Table S1). The majority of the compounds were nucleoside 5′-oligophosphates but varied in their nucleobase type, oligophosphate chain length (from mono- to triphosphate), and the presence of additional phosphate, ribose, and nucleobase modifications (Fig. 1). The phosphate modifications included single-atom substitutions that yielded phosphorothioate (O to S), phosphoroselenoate (O to Se), boranophosphate (O to BH_3_), fluorophosphate (O to F), phosphoroamidate (O to NH), methylenebisphosphonate (O to CH_2_), imidodiphosphate (O to NH), and their combinations as well as substitutions with bulkier groups. The (deoxy)ribose modifications involved substitutions of the 3′ (or 2′) hydroxyl groups with various moieties differing in size and electronegativity. The unmodified nucleotides were purchased from commercial sources, the nucleotide analogues were synthesized previously and taken from our ‘in-house’ collection (see Supplementary Table S1 references and Methods), and the^18^O-labelled isotopologues were synthesized for the purpose of this work using standard nucleotide chemistry and H_2_^18^O, as described in the Methods and shown in Supplementary Fig. S2.

Mass spectra were recorded on a triple quadrupole spectrometer with an electrospray ion source (API 3200 Sciex). The ion source parameters were adjusted to ensure that the intensity of the [M-H]^−^ ion was 5–30%, as these parameters gave optimal fragmentation patterns (Supplementary Fig. S3). The initial optimization experiments, performed on AMP and ADP, revealed that the fragmentation patterns were reproducible under a variety of conditions differing in parameters such as nucleotide concentration, solvent composition, and flow rate (Supplementary Fig. S4). Moreover, we confirmed that fragmentation pathways are highly similar on different mass spectrometers with electrospray ionization, such as Q Exactive Thermo, Q TOF Waters, Q TRAP 3200 Sciex, and API 3200 Sciex (Supplementary Info, Appendix A).

To make the results of our study easily available to the scientific community, we collected our results in a newly created database (msTide) available at www.mstide-db.com. The database contains the MS/MS spectra exported from the original data files for all studied nucleotides with a minimum intensity threshold of 1%. The software enables the browsing of spectra using different kinds of queries, comparison of selected spectra, and identification of compounds with spectra most similar to the spectrum introduced as a query. A more detailed description of msTide database functionality is available in the Supplementary Information and at http://mstide-db.com/static/description.html.

### Fragmentation in negative-ion mode, ESI(−), provides more structural information than positive-ion mode

Nucleotides contain both basic nitrogen atoms within the nucleobase and acidic phosphate groups and, as such, can essentially be effectively ionized both in positive-ion and in negative-ion mode. Therefore, we first compared the fragmentation spectra of selected nucleotides in the two modes (Supplementary Fig. S5). The fragmentation spectra recorded in positive-ion mode contained signals derived only from the nucleobase structure (BH^+^ and fragmentation thereof). Signals derived from phosphate or ribose moieties were either of very low intensity or not observed in the spectra, indicating that the major fragmentation pathways for nucleotides involve base protonation and glycosidic bond cleavage, as previously reported for nucleosides^29^. In contrast, the negative-mode spectra generally contained more fragmentation signals, and those signals were derived from all three structural subunits, i.e. nucleobase, ribose, and phosphate (or their combinations). This suggests that nucleotide fragmentation in negative mode can proceed via multiple pathways, resulting in the generation of greater numbers of structurally diverse fragmentation ions. As such, the negative-ion mode should be more useful in structural determination of modified nucleotides.

### ESI(−)/MS/MS fragmentation spectra of unmodified nucleoside 5′-monophosphates (NMPs) contain three types of signals: nucleobase- (B), phosphoribose- (RbP), and phosphate-derived (P)

**Figure 2:**
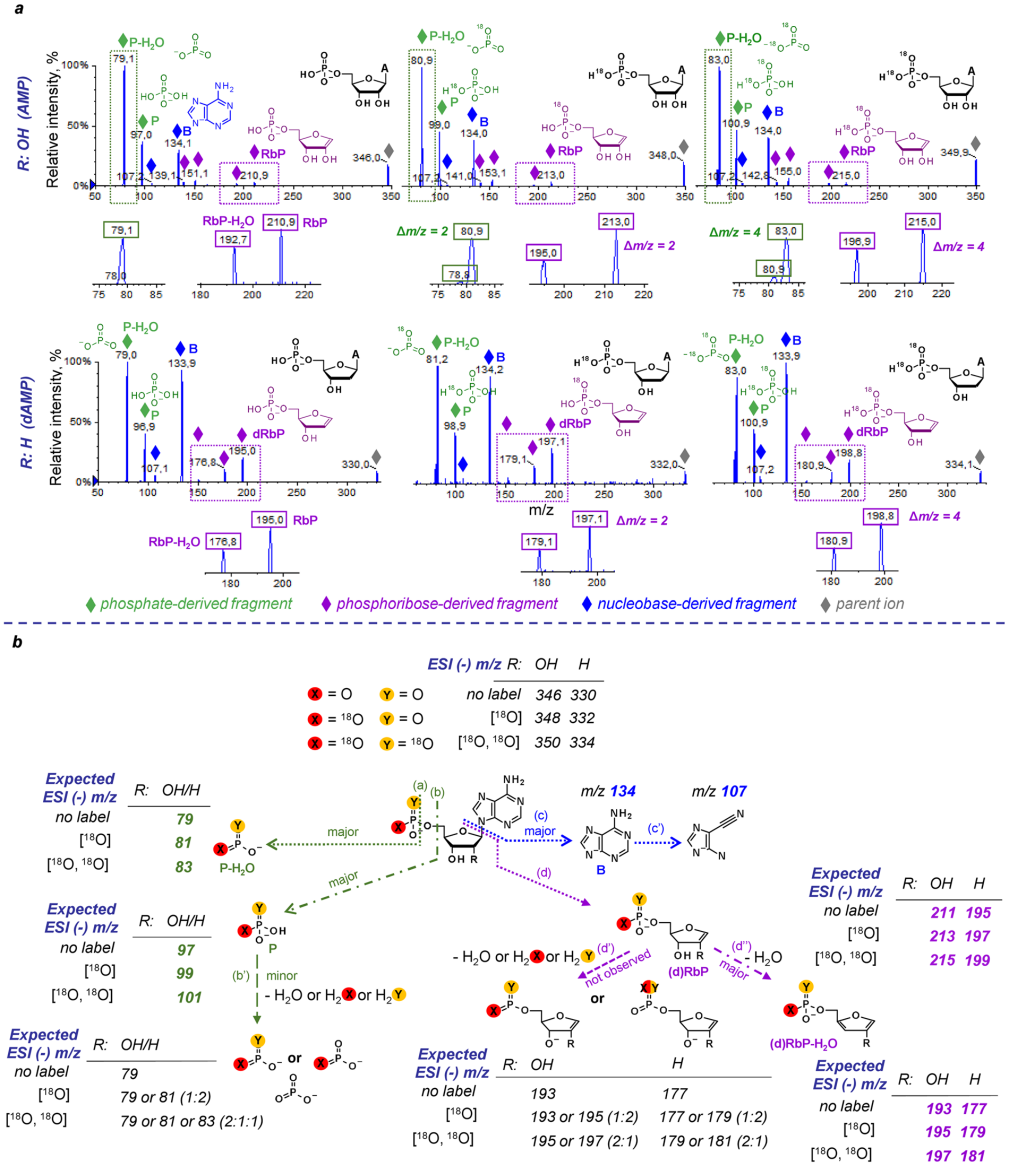
Negative-ion mode fragmentation of nucleoside 5′-monophosphates (NMP). (**a**) MS/MS spectra of AMP and dAMP and their [^18^O]-labelled analogues. Three types of fragments are visible in the spectra: nucleobase derived (marked with blue dots), phosphoribose-derived (purple dots), and phosphate-derived (green dots). Upon^18^O-isotopic labelling of phosphate moieties, the *m/z* values for phosphate- and phosphoribose-derived fragments that retain heavy water shift accordingly. (**b**) Fragmentation pathways for NMPs, exemplified by adenine nucleotides, proposed based on the interpretation of MS/MS spectra of nucleoside 5′-monophosphates and their ^18^O-isotopologues.

We analysed the ESI(−)/MS/MS spectra of six natural ribonucleoside and deoxyribonucleoside 5′-monophosphates in negative-ion mode. Representative spectra of AMP and dAMP are shown in Fig. 2a. A comparison of these spectra revealed that three main types of signals could be identified: nucleobase-derived (B; for unmodified bases A, G, C, and U at *m/z* 134, 150, 111, and 112, respectively, and the products of their fragmentation), phosphate-derived (P at *m/z* 97 and P-H_2_O at *m/z* 79), and phosphoribose-derived (RbP at *m/z* 211 and dRbP at *m/z* 195 for ribo- and deoxyribonucleotides, respectively, and the fragmentation thereof).

Signals resulting from nucleobase fragmentation were assigned based on previously published works on the fragmentation of nucleosides^30–32^. The identities of fragments corresponding to (deoxy)phosphoribosyl moieties (RbP and dRbP) and their dehydration and/or ribose fragmentation products (RbP-H_2_O at *m/z* 193, ribonucleotides at *m/z* 151 and 139, dRbP-H_2_O at *m/z* 175, and deoxyribonucleotides at *m/z* 151 and 139) were verified using^18^O-isotopic substitutions within the phosphate moiety (Fig. 2b). The comparison of spectra for [^18^O]- and [^18^O,^18^O]-substituted AMP and dAMP isotopologues with those of AMP and dAMP revealed that all fragments assigned as P- or RbP-derived showed + 2 or + 4 *m/z* shifts consistent with the presence of heavy oxygen atom(s). Additionally, we observed that all RbP- or dRbP-derived signals retained the initial number of heavy oxygen atoms upon fragmentation, indicating that all dehydration events of the RbP moiety occur exclusively within the ribose and not the phosphate (Fig. 2). In the case of unlabelled AMP, the RbP fragment (*m/z* 211) can be dehydrated in the phosphate moiety or in the ribose moiety, resulting in fragments at *m/z* 193 in both cases. However, if a phosphate-substituted heavy AMP is used, the expected *m/z* values of the dehydrated fragments differ depending on the dehydration site (Fig. 2b). Comparison of the actual ESI(−)/MS/MS spectra of AMP and dAMP indicated that the dehydration occurs exclusively at the ribose moiety. A similar observation was made for fragments at *m/z* 139 and 151, indicating that these ions contain a non-dehydrated phosphate moiety and are generated exclusively through the fragmentation and dehydration of the ribose.

We also compared the fragmentation spectra of 5′-AMP and 3′-AMP. Although the spectra were qualitatively similar, the intensity of the RbP signal (*m/z* 211) was much higher for the 3′ isomer than for the 5′, whereas RbP-H_2_O (*m/z* 193) was below the 1% threshold. This is consistent with the fact that 3′-phosphoribose cannot undergo dehydration through the same pathways as those proposed for the 5′ isomer in Fig. 2b.

### Fragmentation spectra of unmodified nucleoside diphosphates (NDPs) and nucleotide triphosphates (NTPs) contain diphosphoribose (RbDP) and diphosphate (DP) signals, while trimetaphosphate (TMP) is exclusively observed in NTPs

Next, we looked at the fragmentation spectra of unmodified (deoxy)nucleoside 5′-diphosphates present in our library (ADP, GDP, m^7^GDP, dCDP, UDP, and CDP). We found that the general fragmentation patterns were similar to those of nucleoside 5′-monophosphates, i.e. with three main types of signals present in the spectra. For all investigated NDPs, the major phosphate-derived signal was present at *m/z* 159 and corresponded to a dehydrated diphosphate (pyrophosphate) moiety (DP-H_2_O) (Supplementary Fig. S6). The main ribose-derived signal for NDPs was the dehydrated diphosphoribose (RbDP-H_2_O) at *m/z* 273 (relative intensity 7–12%). For dCDP, an analogous signal corresponding to dehydrated phosphodeoxyribose (dRbDP-H_2_O) was observed at *m/z* 257 (5%). The hydrated ions, RbDP (*m/z* 291) and dRbDP (*m/z* 275), were either of lower intensity than the corresponding dehydrated ions (m^7^GDP: 11%, UDP: 1%, dCDP: 2%) or not observed (i.e. below the 1% threshold: GDP, ADP). Signals previously identified for NMPs were often also found in the spectra of NDPs. The assignment of new signals was again verified using heavy oxygen labelling (Supplementary Fig. S6a). A comparison of the MS/MS spectra for GDP and its beta-phosphate-labelled isotopologue, GDPβ-[^18^O], revealed that, in contrast to NMPs, in the case of NDPs, the first dehydration occurred at the phosphate moiety and the second within the ribose moiety (Supplementary Fig. S6b). The spectra also indicated partial migration of the heavy oxygen atom from the β- to α-position, which can be explained by the formation of a cyclic diphospho intermediate (Supplementary Fig. S6c).

The fragmentation spectra of nucleoside 5′-triphosphates (ATP, GTP, UTP, m^7^GTP, dCTP, and CTP) were very similar to those of corresponding 5′-diphosphates, with DP and RbDP-H_2_O as the main phosphate- and phosphoribose-derived signals (Supplementary Fig. S7). Interestingly, we did not observe signals that could be assigned to TP, RbTP, or their dehydrated derivatives. This indicates that one of the first fragmentation events in the case of NTPs is the loss of the γ-phosphate, which is consistent with the chemical lability of the β,γ-pyrophosphate bond^33^. Consequently, the fragmentation of NTPs is expected to occur through the same pathways as those described for NDPs. The only fragmentation ion unique to NTPs was a signal observed at *m/z* 239, which was assigned to a (cyclic) trimetaphosphate moiety (TMP). This is also consistent with the chemical reactivity of NTPs, which are known to form nucleoside 5′-cyclotriphosphates under the action of condensing agents^34^. The identities of the fragmentation ions were also confirmed by ^18^O-isotopic substitution at the γ-position (Supplementary Fig. S7b).

### Phosphate and ribose modifications in NMPs, NDPs, and NTPs are retained in the fragmentation ions

**Figure 3:**
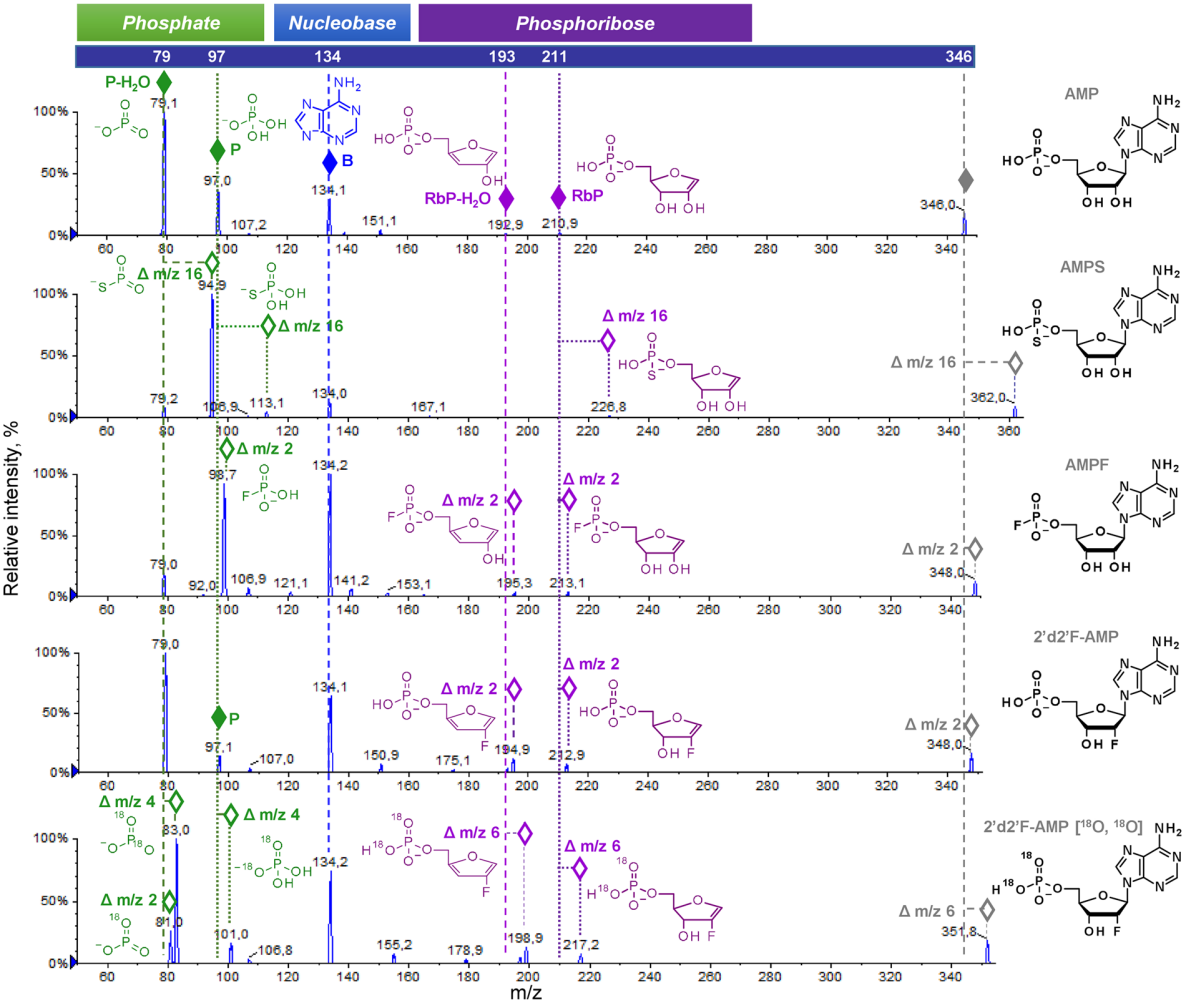
Analysis of phosphate- and ribose-modified AMP analogues by ESI(−)/MS/MS. In each spectrum, fragmentation ions enabling determination of the substitution site (nucleobase *versus* phosphate *versus* ribose) are indicated with diamonds (◊) and their proposed structures are depicted.

To establish the influence of particular modifications on nucleotide fragmentation patterns, we analysed a set of different nucleotide analogues carrying ribose, phosphate, and nucleobase modifications. We first analysed how simple, single-atom substitutions in nucleotides influence the fragmentation spectra by comparing them to those of the parent compounds. We found that all non-isobaric modifications resulted in corresponding *m/z* shifts for fragmentation signals derived from the modified part of the nucleotide, as exemplified by the analysis of several non-isobaric AMP (Fig. 3), ADP (Supplementary Fig. S8), and ATP (Supplementary Fig. S9) analogues. This simple observation enables the unambiguous determination of which part of the analysed nucleotide – the nucleobase, ribose, or phosphate moiety – is modified based on the MS/MS fragmentation spectrum. Namely, if the modification is present in the nucleobase, the *m/z* value of the nucleobase signal changes according to the mass difference, while phosphate and ribose signals remain unchanged. If the modification is in the phosphate moiety, *m/z* value changes are observed for both for RbP and P signals but not for nucleobase signals (Figs 3 and S8). Finally, if the modification is placed within the ribose moiety, an *m/z* value shift is observed only for RbP but not for the P or nucleobase signals. Importantly, we did not notice migration of substituents between ribose, phosphate and nucleobase even for relatively labile substituents such as acyl moieties (compound IDs: 524.1.1, 579.1.1).

**Figure 4:**
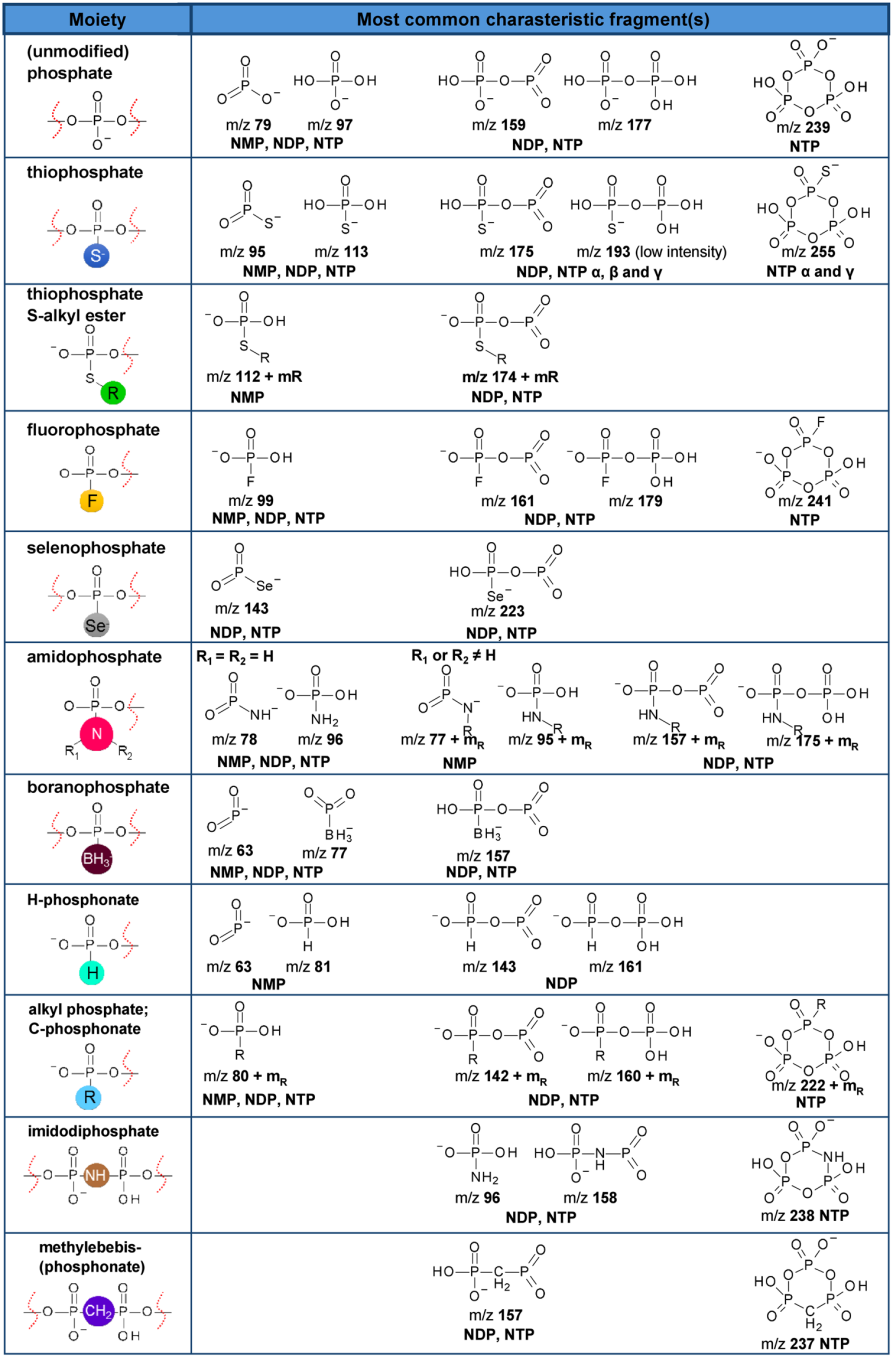
Characteristic fragmentation ions present in the ESI(−)MS/MS spectra of phosphate-modified nucleotides.

For NDP and NTP analogues, similar changes in the fragmentation spectra were observed as a result of modification. However, the loss of the phosphate modification was observed for compounds with high chemical labilities, such as nucleotides carrying boranophosphate or selenophosphate groups, or NTPs or NDPs containing a thiophosphate or sulphate moiety at the terminal position of the oligophosphate chain. For instance, in the case of boranophosphate nucleotide analogues (i.e. carrying an O-to-BH_3_ substitution) one of the fragmentation pathways occurred via P-B bond cleavage to yield H-phosphonate. For bridging modifications, especially imidodiphosphate (i.e. bridging O-to-NH substitution), signals indicating migration of the modification between the α/β and β/γ positions were observed. Signals characteristic for various phosphate modifications are listed in Fig. 4.

### Structurally similar isobaric and isomeric NMP, NDP, and NTP analogues can be differentiated based on MS/MS spectra

**Figure 5:**
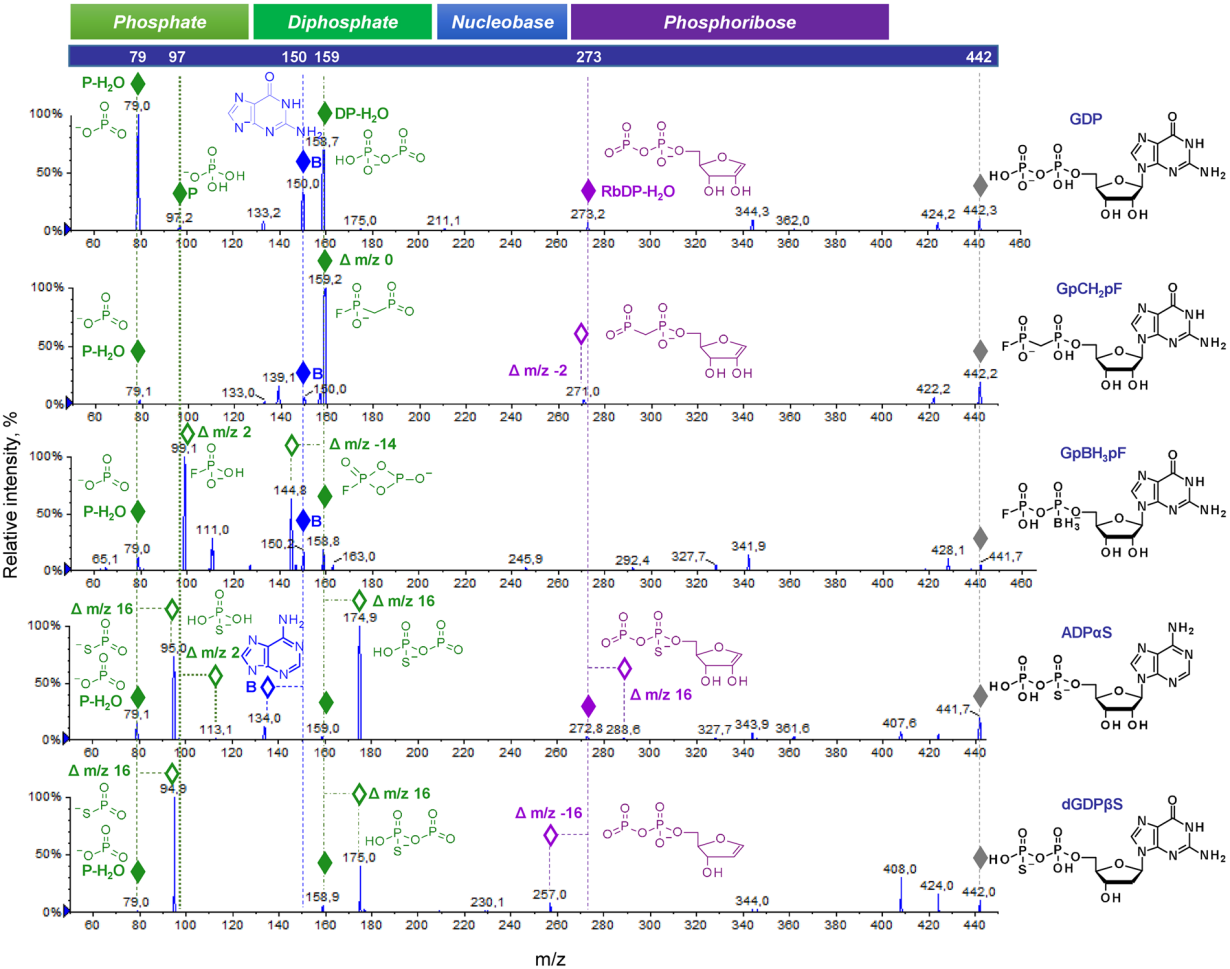
Analysis of phosphate- and ribose-modified nucleotides isobaric with GDP (*m/z* 442) by ESI(−)/MS/MS. In each spectrum, fragmentation ions enabling determination of the modification site(s) (nucleobase *versus* phosphate *versus* ribose) are indicated with diamonds (◊) and their proposed structures are depicted.

Many naturally occurring nucleotides have structurally similar isobars and isomers that are abundantly present in cells. Hence, one of the most challenging tasks in the analysis of nucleotides from biological samples, such as cell and tissue extracts, is the differentiation of the analyte of interest from isobaric (i.e. with the same nominal mass), naturally occurring compounds, especially those with very similar chromatographic properties^35, 36^. Our library contained a number of subsets of isobaric and isomeric nucleotides; therefore, we compared their MS/MS spectra to look for potential differences. We analysed the spectra of GDP (*m/z* 442) isobars by assigning phosphate- and phosphoribose-derived signals for each compound based on previously established nucleotide fragmentation pathways (Fig. 5). We observed distinct fragmentation patterns for each compound, enabling the unambiguous determination of the modifications present.

**Figure 6:**
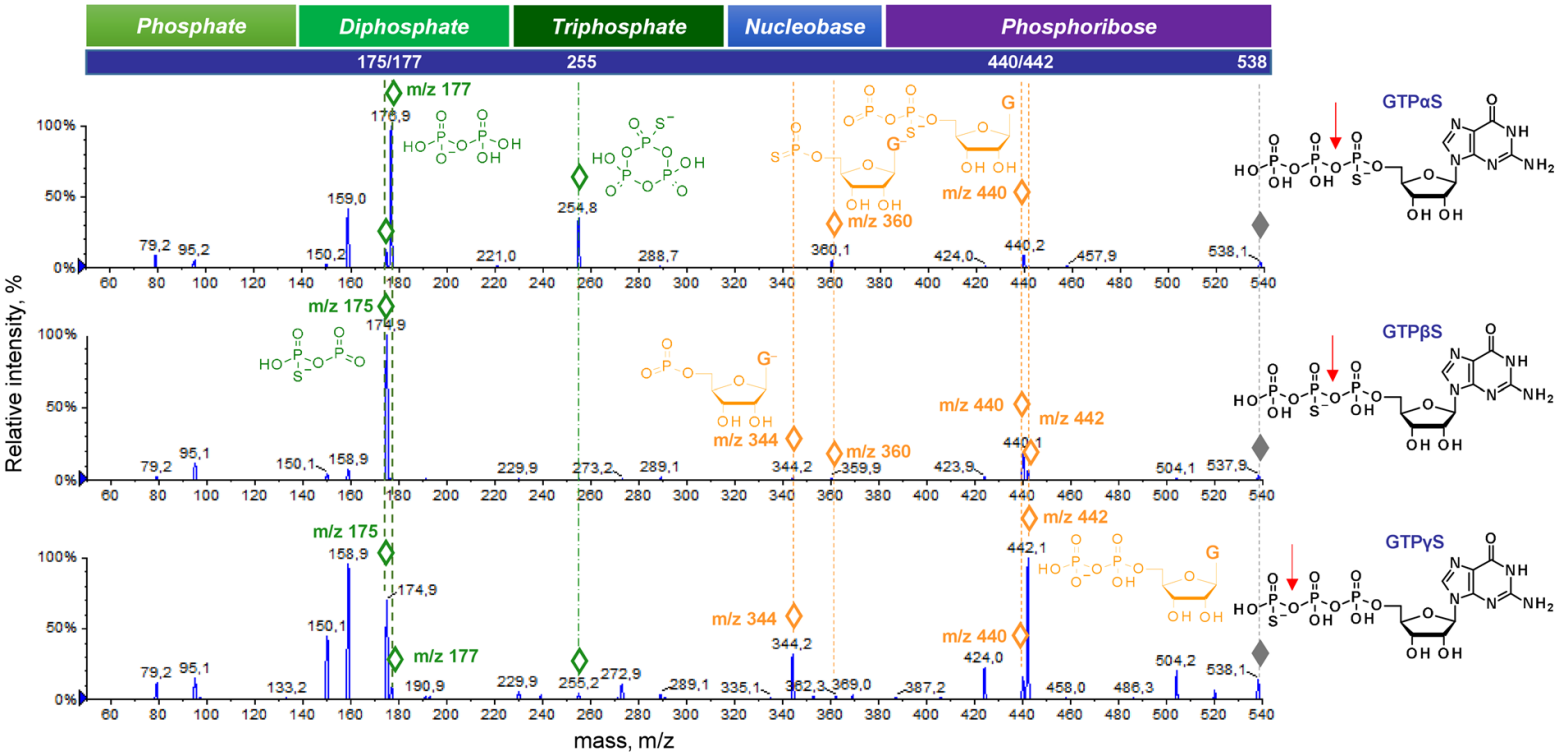
ESI(−)/MS/MS spectra of three GTPS isomers. In each spectrum, fragmentation ions crucial for the determination of the O-to-S substitution site (P_α_ *versus* P_β_ *versus* P_γ_) are indicated with diamonds (◊) and their proposed structures are depicted. Red arrow above each compound indicates pyrophosphate bond most susceptible to cleavage.

Next, we asked whether MS/MS analysis could provide insight into closely related isomers. As an example, we selected a set of GTP analogues carrying an O-to-S substitution at the α-, β-, or γ-position of the triphosphate chain (Fig. 6). We found that despite their structural similarities, the fragmentation patterns for the three compounds were distinct, and differences could be explained on the grounds of chemical stability. Phosphate-derived fragmentation ions of the highest intensity indicated that the fragmentation occurred primarily by the cleavage of the phosphate-thiophosphate anhydride bond, which is less stable than the unmodified pyrophosphate bond^37^.

### MS/MS analysis can be applied to rapid identification of nucleotide synthesis products

**Figure 7:**
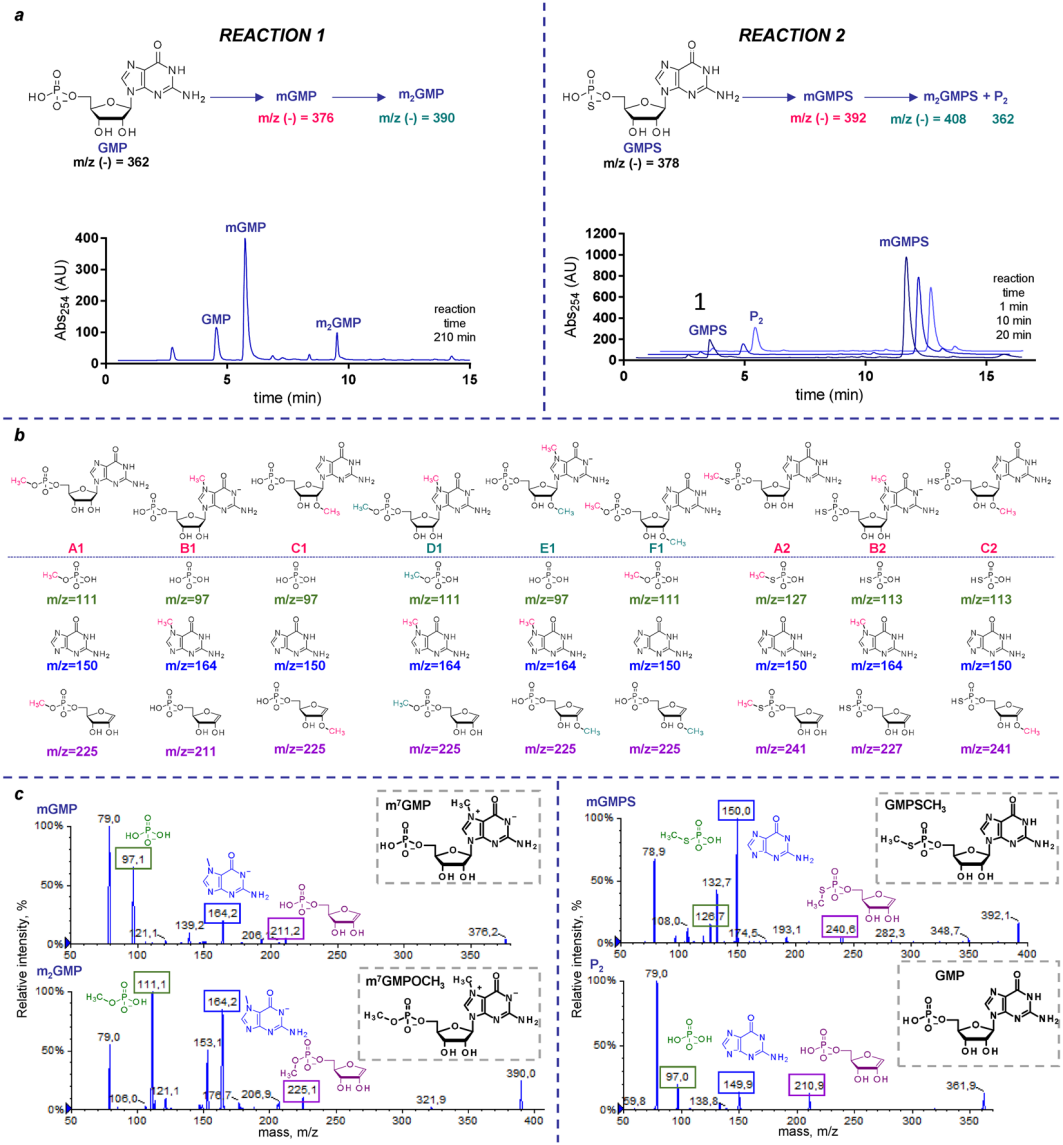
ESI(−)/MS/MS analysis of guanine nucleotide methylation products. (**a**) Scheme of the investigated reactions and representative RP HPLC chromatograms. (**b**) Possible reaction products and predicted fragments expected to be found in ESI(−)/MS/MS spectra for each of them. (**c**) Actual ESI(−)/MS/MS spectra and proposed structures of the products. The structures were independently confirmed by ^1^H and ^31^P NMR (Figs S10 and S11).

MS/MS analysis has unquestionable potential as a tool to solve biological problems, but as nucleotide chemists, we envisaged that the insights provided by MS/MS analysis could also efficiently support synthetic nucleotide chemistry. The synthesis and purification of modified nucleotides are challenging in many respects, making it difficult and time-consuming to monitor reactions using standard techniques such as ^31^P NMR. Therefore, we speculated that MS/MS fragmentation, when combined with knowledge of nucleotide fragmentation patterns, would be a complementary technique that could be helpful in the rapid analysis of nucleotides, especially those synthesized at a very small scale, allowing for the analysis of complex reaction mixtures and identification of by-products without isolation. We demonstrated this by analysing the products of the methylation reactions of two guanine nucleotides, guanosine 5′-phosphate (GMP) and guanosine 5′-thiophosphate (GMPS), by methyl iodide in DMSO (Fig. 7). The methylation of GMP (*m/z* 362) for 60 min yields two products, a single methylated main product (mGMP, *m/z* 376) and a double-methylated side-product (m_2_GMP, *m/z* 390), while the methylation of GMPS (*m/z* 378) almost instantaneously yields a single methylated product (*m/z* 392), which then quickly converts into an unknown product, P_2_ (*m/z* 362), over time and upon aqueous reaction work-up (Fig. 7a). Considering the nucleobase, phosphate, and ribose moieties as three potential methylation sites, we predicted the fragmentation ions that should be observed in the case of single or double methylation at any of them (Fig. 7b). Analysis of the MS/MS spectra of the actual compounds (Fig. 7c) clearly indicated that mGMP is nucleobase-methylated, m_2_GMP is nucleobase- and phosphate-methylated in agreement with the literature^38^, and mGMPS is exclusively phosphate-methylated. We also analysed the MS/MS spectrum of the side-product P_2_ (*m/z* 362), which was similar to the spectrum of GMP (Fig. 7c). The structures of all products (mGMP, m_2_GMP, mGMPS and P_2_) were confirmed by monitoring the reaction time-courses by ^1^H and ^31^P NMR (Figs S10 and S11).

## Conclusions

In this work, we analysed a unique library of over 150 small nucleotides and their phosphate-, ribose-, and nucleobase-modified analogues by ES(−)/MS/MS and collected the results in an electronic database (www.mstide-db.com). The findings of our work can be summarized by several general observations:

- The ESI/MS/MS spectra in negative mode, ESI(−), contained more signals and thereby more structural information than the spectra in positive mode, ESI(+).
- ESI(+)/MS/MS spectra of nucleotides contain fragmentation ions derived from the nucleobase only, with ions derived from ribose or phosphate not observed or observed at very low intensities. Therefore, ESI(+) mode has little utility for the identification of phosphate or ribose modifications present in nucleotides.
- ESI(−)/MS/MS spectra of nucleotides contain fragmentation ions of three different types: nucleobase-derived, (oligo)phosphate-derived, and (oligo)phosphoribose-derived.
- The intensity of a particular ion-type in the spectrum depends on the nucleotide structure and subtle differences in chemical stability; however, (oligo)phosphate-derived signals are usually of high intensity, nucleobase-derived signals are usually of high or moderate intensity, and (oligo)phosphoribose-derived signals are usually of moderate to low intensity.
- The ESI(−)/MS/MS spectra of ribose- and phosphate-unmodified NMPs contain a RbP signal at *m/z* 211 of low to moderate intensity (often together with a dehydrated form at *m/z* 193), together with phosphate signals (P and P-H_2_O) at *m/z* 97 and 79, respectively, of high to moderate intensity.
- The ESI(−)/MS/MS spectra of ribose- and phosphate-unmodified NDPs and NTPs contain a RbDP-H_2_O signal at *m/z* 273 of low to moderate intensity (often together with *m/z* 293 or 255) and diphosphate signals (DP and DP-H_2_O) at *m/z* 177 and 159, respectively, of high to moderate intensity.
- If a non-isobaric ribose modification is introduced into a nucleotide, only the *m/z* values of ribosophosphate-derived signals may change according to the mass difference.
- If a non-isobaric phosphate modification is introduced into a nucleotide, the *m/z* values of both phosphoribose-derived and phosphate-derived signals may change according to the mass difference.
- If a non-isobaric nucleobase modification is introduced into a nucleotide, only the *m/z* values of nucleobase-derived signals may change according to the mass difference.
- The presence of modifications influences fragmentation pathway contributions according to the chemical labilities of the introduced modifications, enabling the differentiation of even structurally similar isobaric and isomeric compounds.

We envisage that the results of our research, available in the msTide database, will be useful to researchers investigating nucleotide-based drug and prodrug metabolism, the quantification of naturally occurring modified nucleotides, and the discovery of new, non-canonical nucleotides in living organisms. We also demonstrated the potential for applying MS/MS analysis in synthetic nucleotide chemistry for the rapid identification of small-scale reaction products as a complementary method to other, less-sensitive techniques, such as NMR.

## Methods

### Chemicals

MS-grade reagents (methanol, acetonitrile, and ammonium acetate) and unmodified nucleotides such as AMP, GDP, ATP, and dCMP were purchased from Sigma-Aldrich. Nucleotide analogues were synthesized by published methods^37, 39–43^. A list of all nucleotides used in our study, including their structures and references for their synthesis, is given in the Supplementary Information (Supplementary Table S1).

If necessary, the compounds were purified by means of analytical HPLC or semi-preparative RP HPLC. Analytical HPLC (Series 1200; Agilent Technology) was performed on a Supelcosil LC-18 T HPLC column (25 cm × 4.6 mm, 5 μm) using a 0–50% linear gradient of solvent B in solvent A within 15 min and a flowrate of 1.3 mL/min. Solvent A was 0.05 M ammonium acetate pH 5.9, and solvent B was a 1:1 (v/v) mixture of solvent A and methanol. Semi-preparative HPLC was performed on the same apparatus equipped with a Discovery RP Amide C-16 HPLC column (25 cm × 21.2 mm, 5 μm, flow rate 5.0 mL/min) with a linear gradient of 0–100% acetonitrile in 0.05 M ammonium acetate buffer (pH 5.9) for 120 min with UV detection at 260 nm.

### Sample preparation

Compounds were dissolved in a water:methanol (1:1, v/v) mixture or a 0.05 M ammonium acetate pH 5.9:methanol (1:1, v/v) mixture to concentrations in the range of 40–400 μM.

### MS conditions

Analysis of nucleotides was performed on API 3200 and QTrap 3200 (AB Sciex) spectrometers with electrospray ionization (ESI) in negative-ion mode. Samples were analysed using direct infusion with a syringe pump (4.6 mm diameter) at flowrates of 10–100 μl/min. An ion spray voltage (IS) of −4500 V, curtain gas (CUR) of 25 psi, high collision gas (CAD), and ion source gas 1 (GS1) of 20 psi were applied. Other parameters were optimized for each compound; typical values included a declustering potential (DP) in the range of −30 to −50 V, entrance potential (EP) in the range of −3 to −10 V, and collision energy (CE) in the range of −30 to −55 V.

### Chemical synthesis

#### Procedure 1

Conversion of nucleosides into 5′ nucleotides and analogues.

#### Procedure 1a

Nucleoside 5′-phosphorylation^44^ (compounds: [^18^O,^18^O]2′-dAMP, AZTMP, 2′-F, 2′-dAMP, [^18^O,^18^O]AMP, [^18^O,^18^O]2′-F, 2′-dAMP, 3′-NH_2_GMP, [^18^O,^18^O]3′-NH_2_GMP; IDs: 335.1.1, 347.5.1, 349.1.1, 351.1.1, 353.1.1, 331.2.1, 362.3.1): Nucleoside (0.01 mmol) was dissolved/suspended in trimethylphosphate (0.5 ml) at 0 °C followed by the addition of POCl_3_ (0.03 mmol). Reaction progress was monitored by RP HPLC and quenched with 97% [^18^O]-H_2_O when conversion of the substrate was in the range of 60–90%. The desired nucleoside 5′-monophosphate was purified using RP HPLC under the conditions given above. The ESI(−)/MS analysis revealed that the isolated products contained [^18^O,^18^O]-labelled nucleoside 5′-monophosphate as the main product along with smaller amounts of single [^18^O]-labelled and unlabelled products.

#### Procedure 1b

Nucleoside 5′-amidophosphorylation (compounds: GMP(NH_2_)_2_, GMPNH_2_; IDs: 361.1.1, 362.1.1): Synthesis was carried out by the procedure described in 1a with the exception that the reaction quenching was carried out with 25% aqueous ammonia followed by pH adjustment to 7 with diluted acetic acid.

#### Procedure 1c

Nucleoside 5′-thiophosphorylation^45^ (compounds: [^18^O,^18^O]AMPS, [^18^O]AMPS; IDs: 367.1.1, 365.1.1): Synthesis was carried out by the procedure described in 1a with the exception that PSCl_3_ (0.03 mmol) and 2,6-lutidine (0.09 mmol) were used instead of POCl_3_.

#### Procedure 1d

Nucleoside 5′-bis(phosphonylation)^46^ (compounds: [^18^O]ApCH_2_p, 2′-F2′-dApCH_2_p; IDs: 427.7.1, 427.6.1): Synthesis was carried out by the procedure described in 1a with the exception that methylenebis(phosphonic dichloride) (0.015 mmol) was used instead of POCl_3_.

#### Procedure 2

P-Imidazolide hydrolysis (compounds: [^18^O]ApCH_2_p, [^18^O]GDP, and [^18^O]GTP; IDs: 427.7.1, 445.2.1., 525.2.1): An appropriate nucleotide P-imidazolide derivative was dissolved in 97% [^18^O]-H_2_O, and the reaction was adjusted to pH ~2 with concentrated HCl. The reaction progress was monitored by RP HPLC. When the hydrolysis was complete, the pH of the reaction mixture was adjusted to 5–6 with NaOH solution. The desired product, β-[^18^O]-labelled nucleoside 5′-diphosphate or 5′-bis(phosphonate), was purified using RP HPLC under the conditions given above.

#### Procedure 3

*N* ^7^-Methylation of nucleotides^47^ (compounds: m^7^GMPNH_2_, m_2_^7,2′-O^GMPOCH_3_, m^7^GMPSCH_3_; IDs: 376.1.1, 405.1.1, 407.2.1): An appropriate guanine nucleotide (0.01 mmol) was dissolved in DMSO, and methyl iodide (0.1 mmol) was added. Reactions were monitored by RP HPLC and quenched with water, washed with diethyl ether, and concentrated under vacuum. The desired products were purified by RP HPLC under the conditions given above.

#### Procedure 4

Metal ion-mediated coupling of P-imidazolides with nucleophiles (compounds: 2′F, 2′-dADP, 2′F, 2′-dATP, dGDPS, m^7^GppH; IDs: 429.2.1, 509.2.1, 443.8.1, 441.2.1): Nucleotide P-imidazolide derivative (0.1 mmol) was dissolved/suspended in DMF, and an appropriate nucleophile (i.e. triethylammonium salt of phosphate, thiophosphate, or H-phosphonate; 0.2–0.4 mmol) was added followed by anhydrous ZnCl_2_ (0.8 mmol). Reactions were quenched with aqueous EDTA (0.8 mmol), the pH was adjusted to 6 with solid NaHCO_3_, and products were purified by RP HPLC under the conditions given above.

*Procedure 5*. Nucleotide 2′,3′-*O,O*-carbonylation (compound: CO-GMPH; ID: 373.1.1): 2′,3′-O,O-carbonyl-guanosine 5′-H-phosphonate was obtained by treatment of guanosine 5′-H-phosphonate with carbonyl diimidazole.

### GMP and GMPS methylation experiments

GMP or GMPS (0.005 mmol) was dissolved in 100 µl DMSO and methyl iodide (0.04 mmol or 0.02 mmol, respectively) was added. Reactions were stirred at r.t. and progress monitored by RP HPLC (UV-detection at 254 nm) by analysing 1 μl aliquots diluted with 10 μl of water. The UV-absorbing peaks were manually collected and analysed by ESI(−) MS/MS (Fig. 7). For the purpose of NMR experiments, GMP or GMPS (0.03 mmol) were dissolved in 600 μl of DMSO-d_6_ and the reactions were performed in a 5 mm NMR tube in analogous manner as described above, except no stirring was applied. NMR spectra were recorded on BRUKER AVANCE III HD spectrometer equipped with 5 mm PABBO BB/ 19F-1H/ D Z-GRD5 probe, at 25 °C. ^1^H NMR spectra were recorded at 500.24 MHz and ^31^P NMR spectra at 202.49 MHz using standard pulse sequences (usually 16 scans for ^1^H and 32 scans for ^32^P). The ^1^H NMR and ^31^P NMR chemical shifts were reported in ppm and referenced to residual DMSO-d_6_ signal or external 20% phosphorus acid in D_2_O, respectively.

### Data availability

The raw fragmentation spectra were exported to txt files using Analyst 1.6.3 software. For technical reasons, only signals that reached the threshold of 1% relative intensity were included in the export process. The datasets generated during and/or analysed during the current study are available in the msTide database, www.msTide-db.com. The original datafiles are available from the corresponding author upon request.

## Electronic supplementary material

Supplementary Info 1
