# Supplementary material for: Analysis of mononucleotides by tandem mass spectrometry: investigation of fragmentation pathways for phosphate- and ribose-modified nucleotide analogues
Source: Sci Rep. 2017 Aug 21;7:8931. doi: 10.1038/s41598-017-09416-6 (PMC5567097; doi:10.1038/s41598-017-09416-6)
Supplement: Supplementary file 1 — Supplementary Info 1 [file 41598_2017_9416_MOESM1_ESM.pdf]

Analysis of mononucleotides by tandem mass spectrometry: investigation of fragmentation pathways for phosphate- and ribose-modified nucleotide analogues

Dominika Strzelecka,[1] Sebastian Chmielinski,[1,2] Sylwia Bednarek,[1,2] Jacek Jemielity,[2] Joanna Kowalska[1,*]

[1]Division of Biophysics, Institute of Experimental Physics, Faculty of Physics, University of Warsaw, Pasteura 5, 02-093 Warsaw, Poland

[2]Centre of New Technologies, University of Warsaw, Banacha 2c, 02-097 Warsaw, Poland

**E-mail: jkowalska@fuw.edu.pl*

Supplementary File

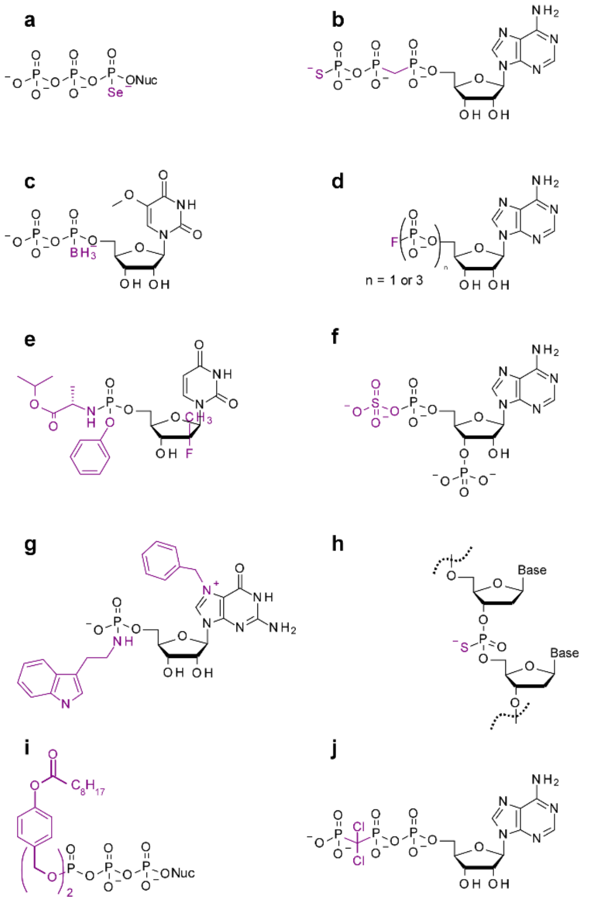

Fig. S1. **Examples of naturally occurring phosphate- and ribose-modified nucleotides or synthetic analogues with biological and therapeutic applications.** a) NTPαSe – polymerase substrates for nucleic acids modification[1]; b) ATPαS,β-γCH_2 – chelating agent with potential in Alzheimer's treatment[2]; c) 5-*O*-MeUDPαBH_3 – agonist of P2Y receptor[3]; d) NMPF, NDPF, and NTPF – unnatural substrates of HIT family enzymes[4]; e) sofosbuvir – anti-viral drug[5]; f) PAPS – natural compound, universal sulfotransferase cofactor[6]; g) 4E-i – prodrug form of translation inhibitor targeting eIF4E protein[7,8]; h) phosphorothioate DNA – synthetic modification used for therapeutic oligonucleotide (ON) stabilization, later discovered in bacteria[9]; i) ProPPNucleotides – cell-permeable NTP prodrugs[10]; j) ATPβ-γCCl_2 – metabolite of bisphosphonate anti-osteoporotic drug, clodronate[11-13]. Nuc = 5'-nucleosidyl moiety, Base = Adenine, Guanine, Cytosine, or Thymine.

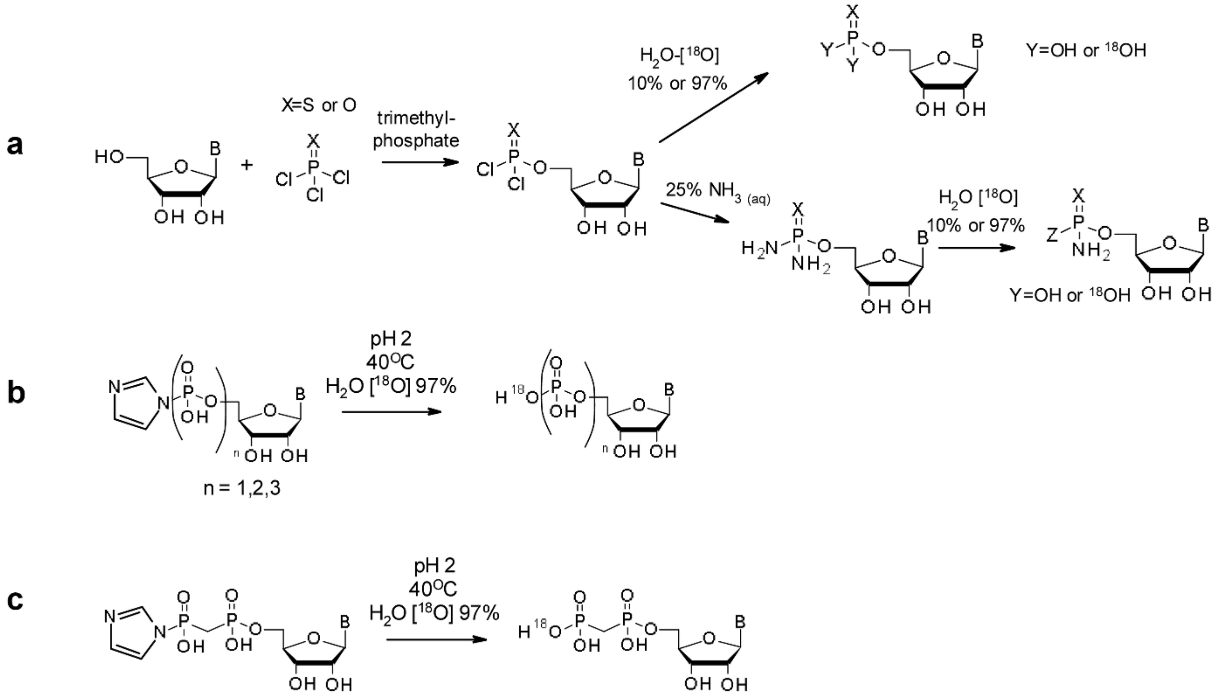

Fig. S2. **Syntheses of isotopically labelled nucleotides carried out in this work:** A - Nucleoside 5'-phosphorylation, nucleoside 5'-thiophosphorylation, nucleoside 5'-amidophosphorylation, B and C - P-Imidazolide hydrolysis.

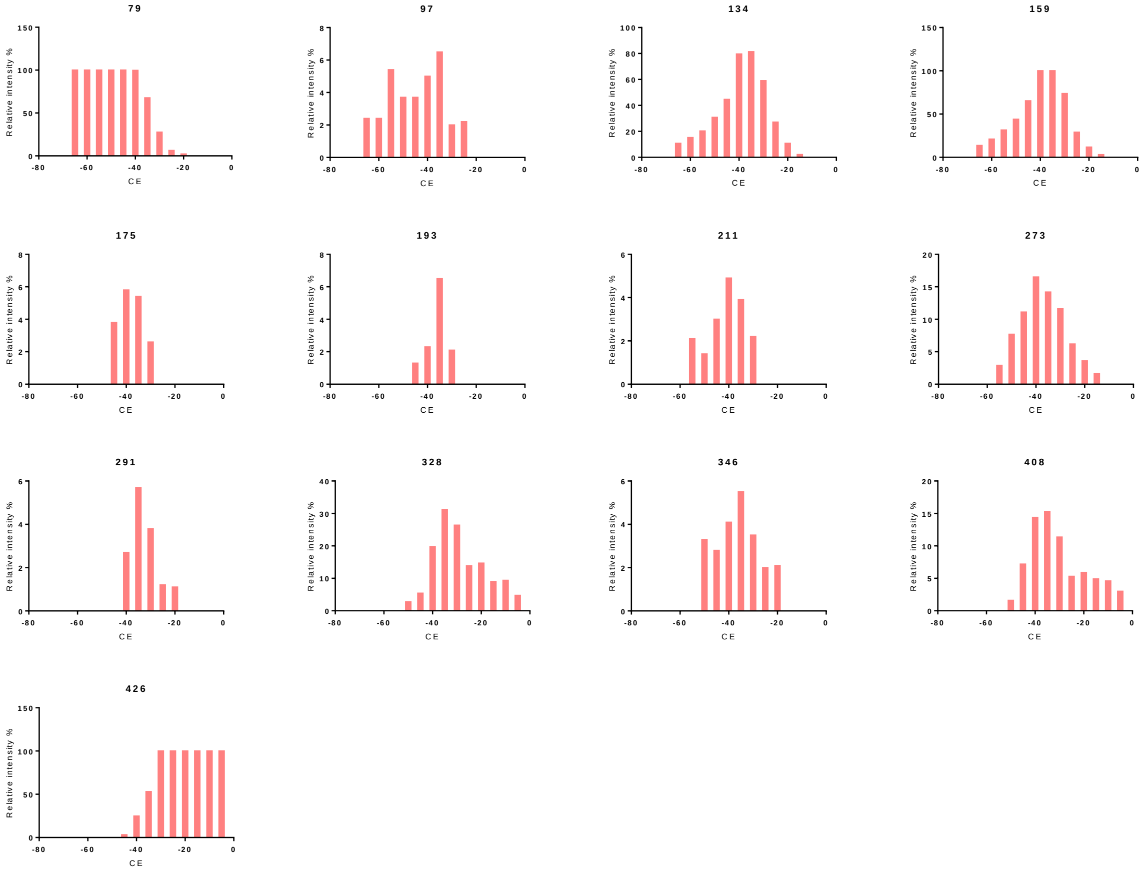

Fig. S3. **Fragment ion relative intensity distribution depending on Collision Energy (CE) for ADP.**

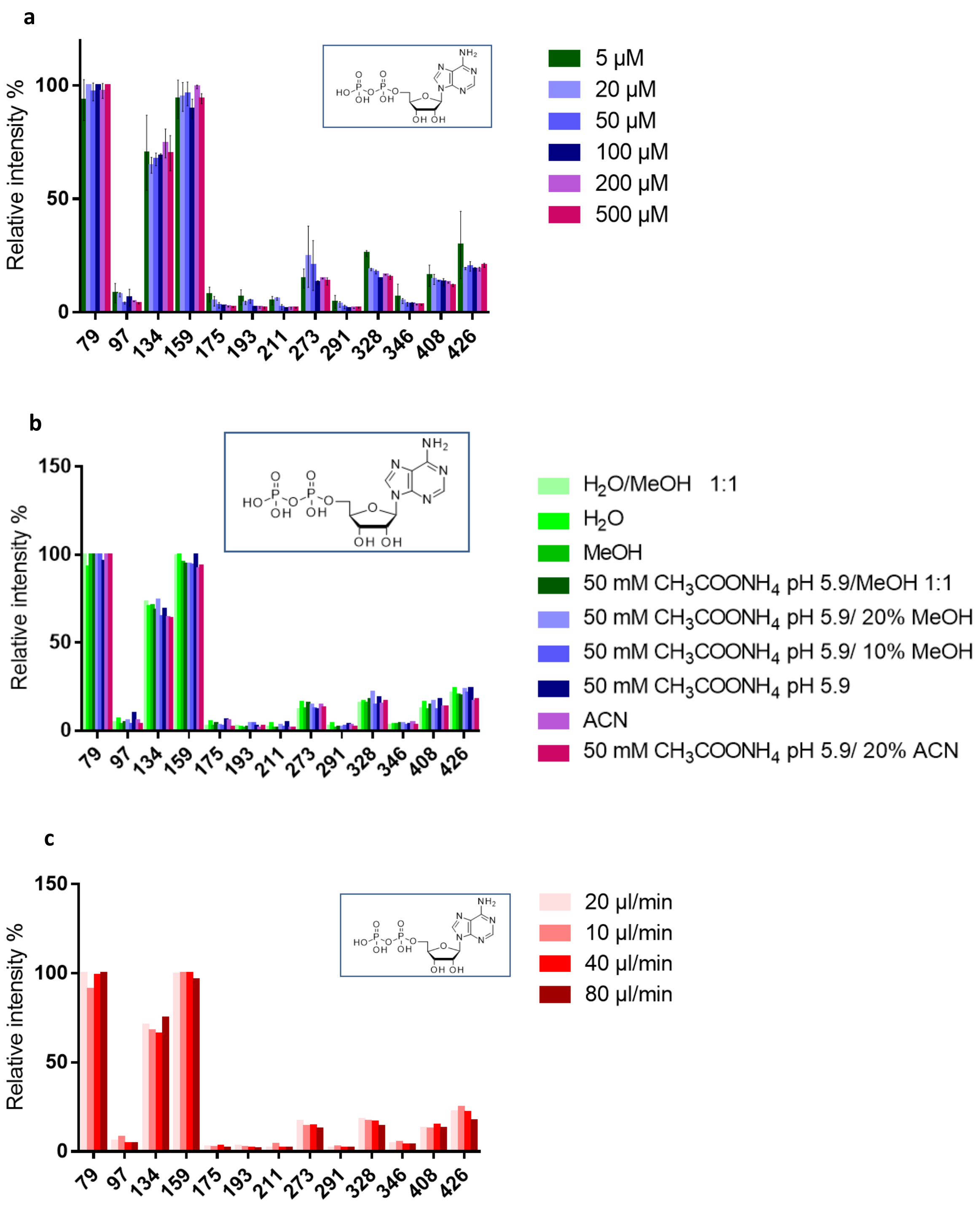

Fig. S4. **Relative ion intensities in ESI(-) MS/MS spectra** of: A) ADP dissolved in 50 mM CH_3COONH_4 pH 5.9/ 20% MeOH at various concentrations. The data shown are mean relative intensities from duplicate experiments +/- S.D. B) 50 μM ADP depending on solvent composition. C. 50 μM ADP in 50 mM CH_3COONH_4 pH 5.9/ 20% MeOH depending on infusion flow rate.

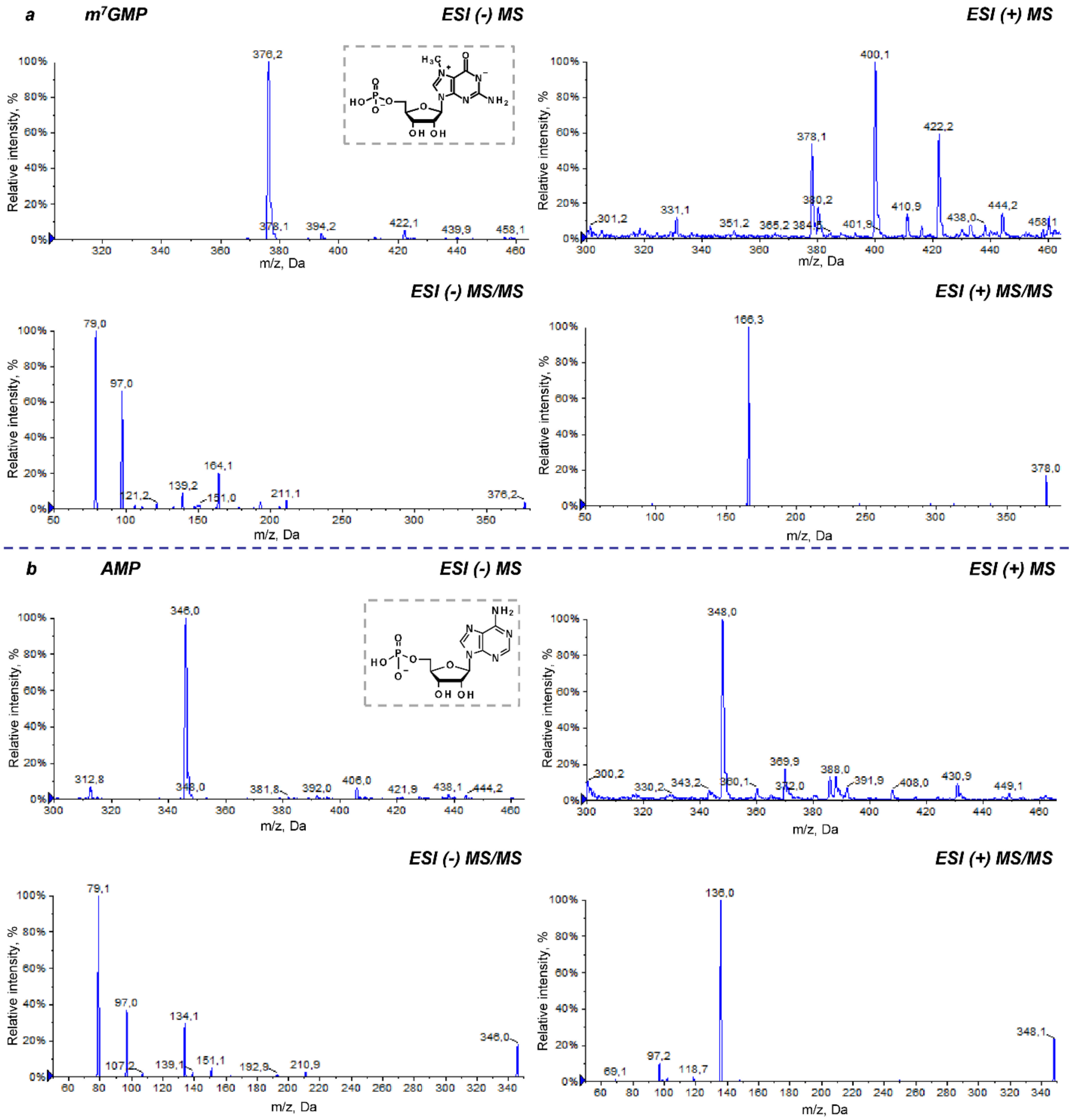

Fig. S5. **Comparison of MS and MS/MS spectra in positive and negative ion mode for m^{7}GMP (A) and AMP (B).**

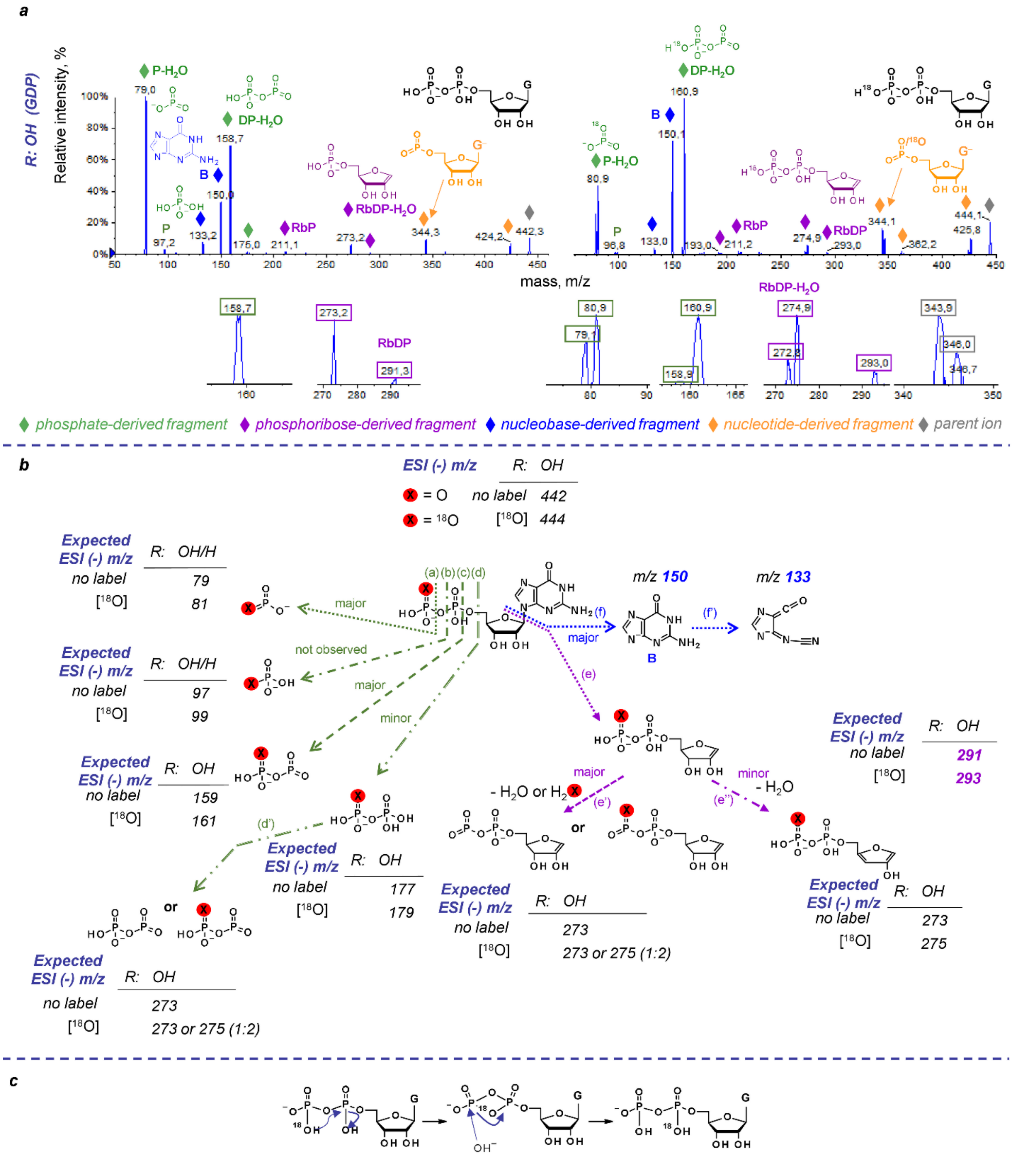

Fig. S6. **Negative-ion mode fragmentation of nucleoside 5'-diphosphates**. a) ESI(-)/MS/MS spectra of GDP and its β-[^{18}O]-labelled analogue. b) Proposed fragmentation pathways for NDPs, exemplified by the fragmentation of GDP. c) Possible mechanism for the transfer of [^{18}O] from the β-phosphate to α-phosphate, explaining the presence of [^{18}O]-labelled guanosine monophosphate derivatives in the MS/MS spectrum of β-[^{18}O]-GDP.

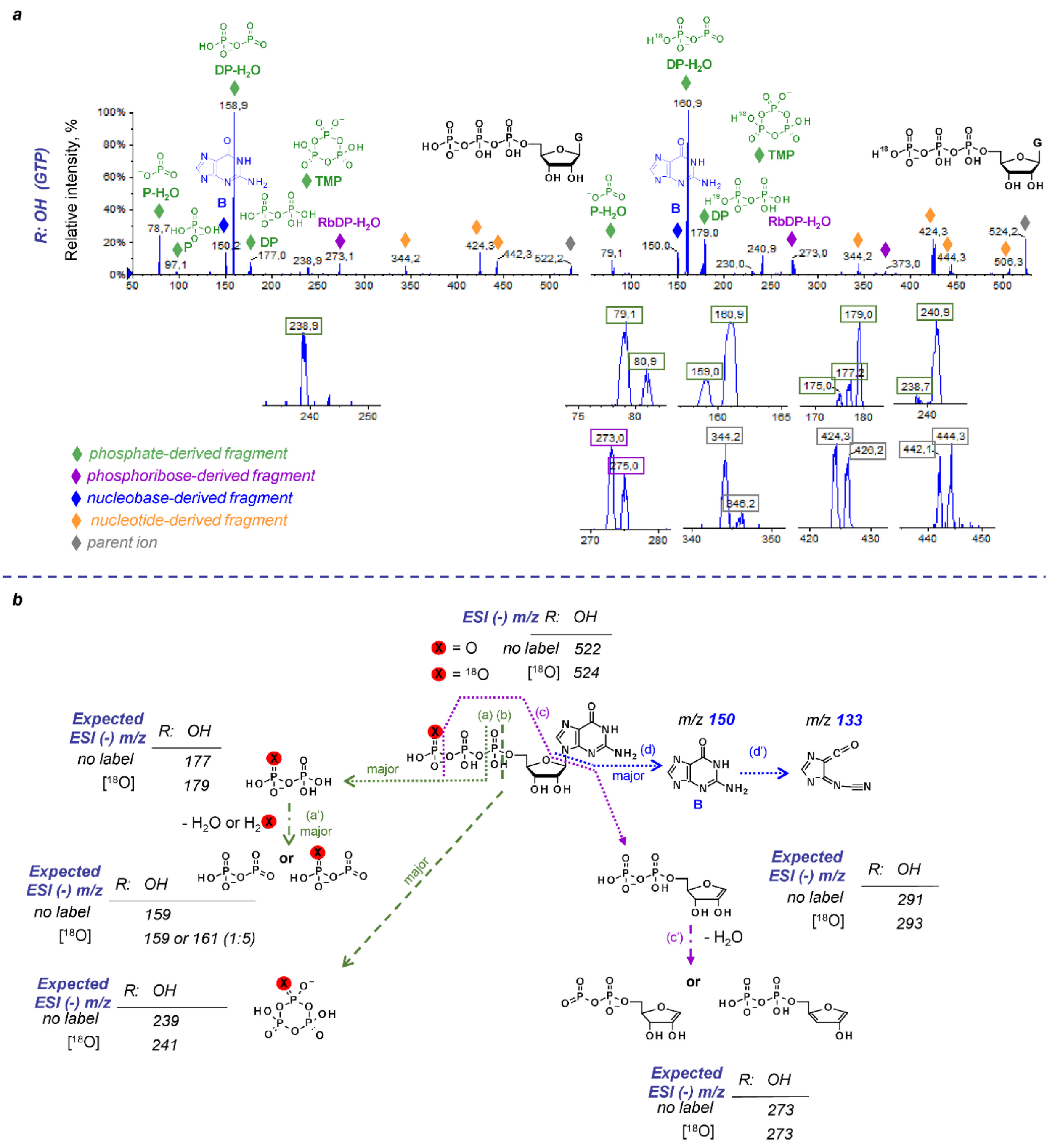

Fig. S7. **Negative-ion mode fragmentation of nucleoside 5'-triphosphates.** a) ESI(-)/MS/MS spectra of GTP and its γ-[^{18}O]-labelled analogue. b) Proposed fragmentation pathways for NTPs, exemplified by the fragmentation of GTP.

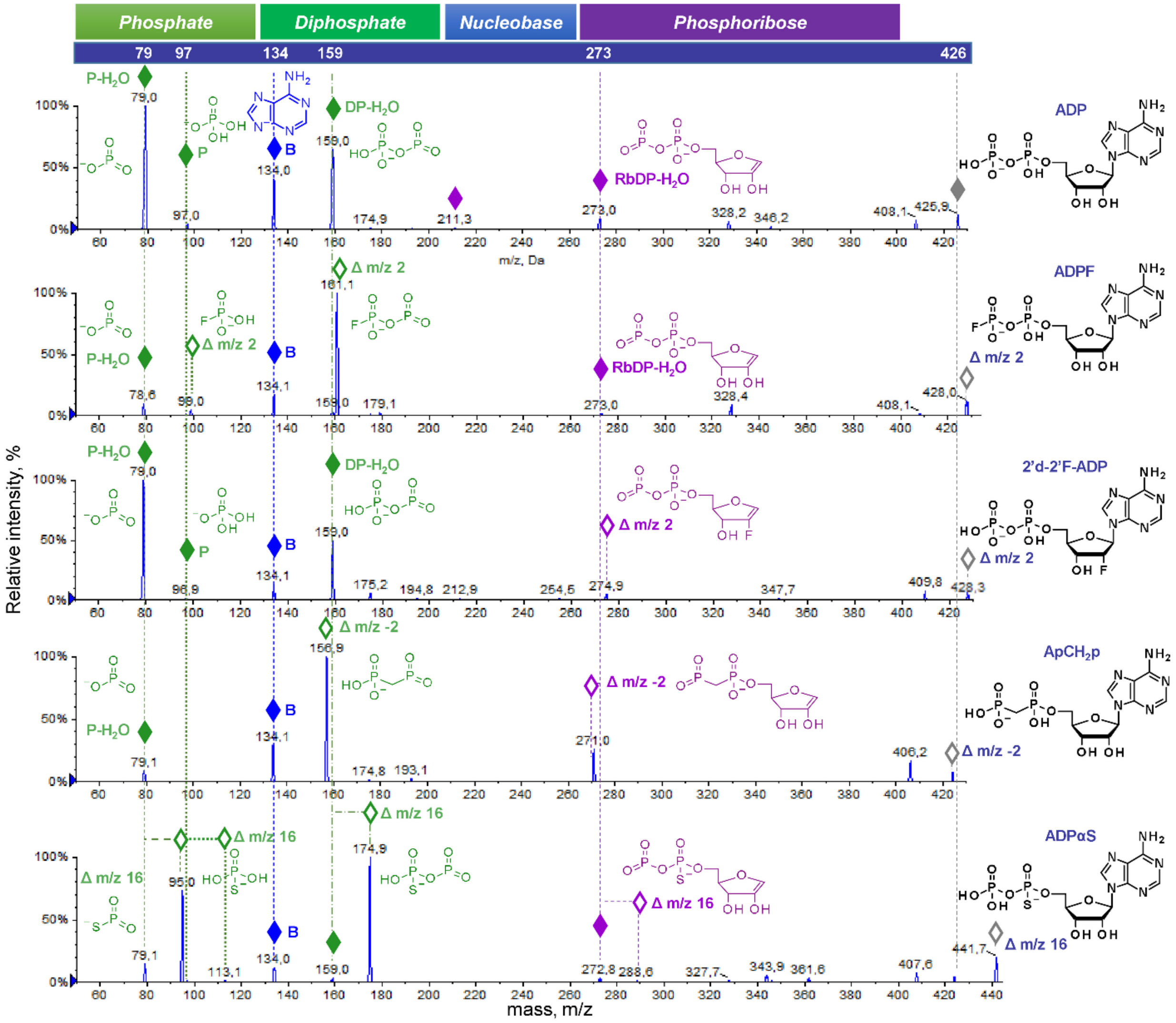

Fig. S8. **Analysis of ADP and its phosphate- and ribose-modified analogues by ESI(-)/MS/MS.** In each spectrum, fragmentation ions enabling determination of the substitution site (nucleobase *versus* phosphate *versus* ribose) are indicated with diamonds (◊) and their proposed structures are depicted.

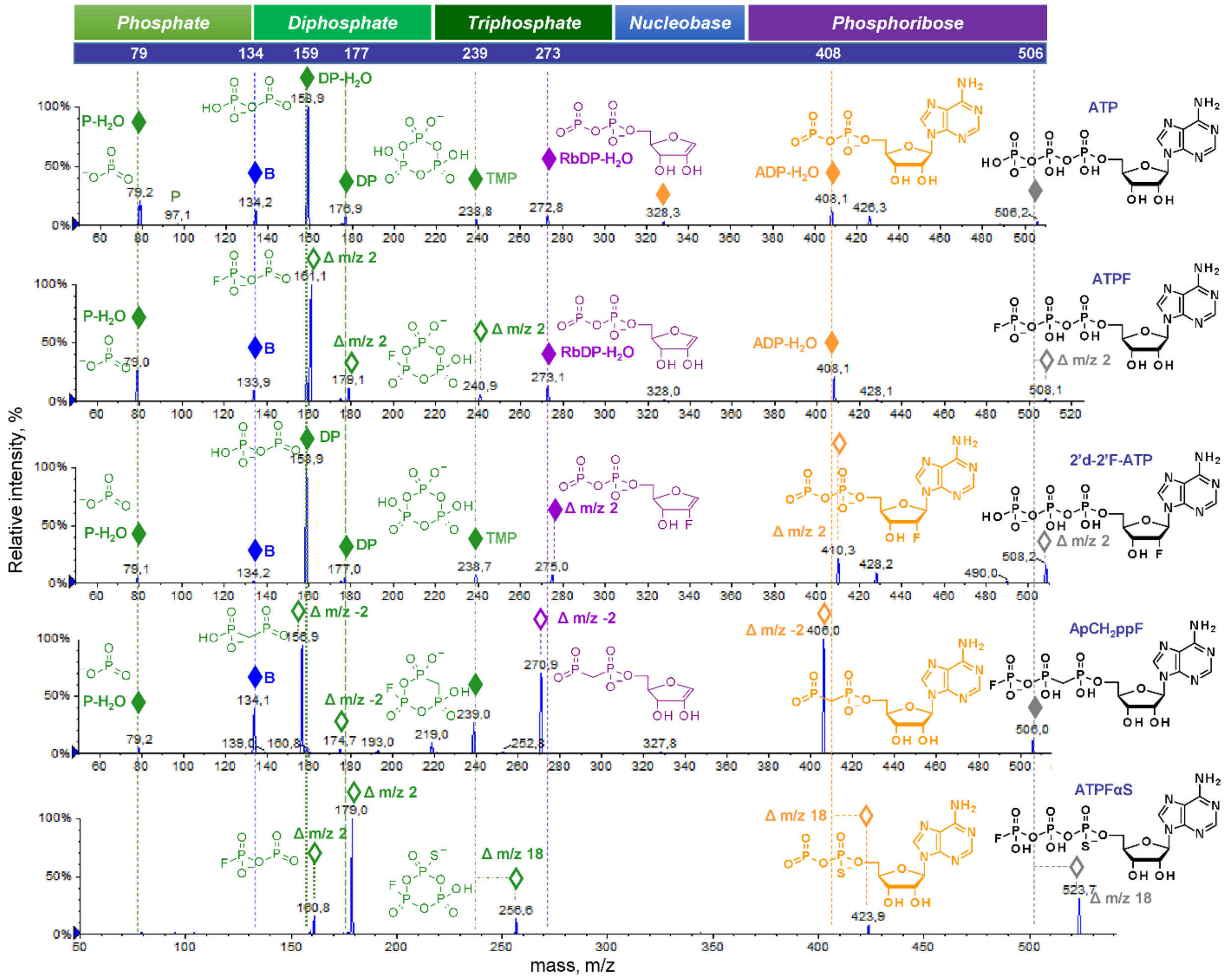

Fig. S9. **Analysis of ATP and its phosphate- and ribose-modified analogues by ESI(-)/MS/MS.** In each spectrum, fragmentation ions enabling determination of the substitution site (nucleobase *versus* phosphate *versus* ribose) are indicated with diamonds (◊) and their proposed structures are depicted.

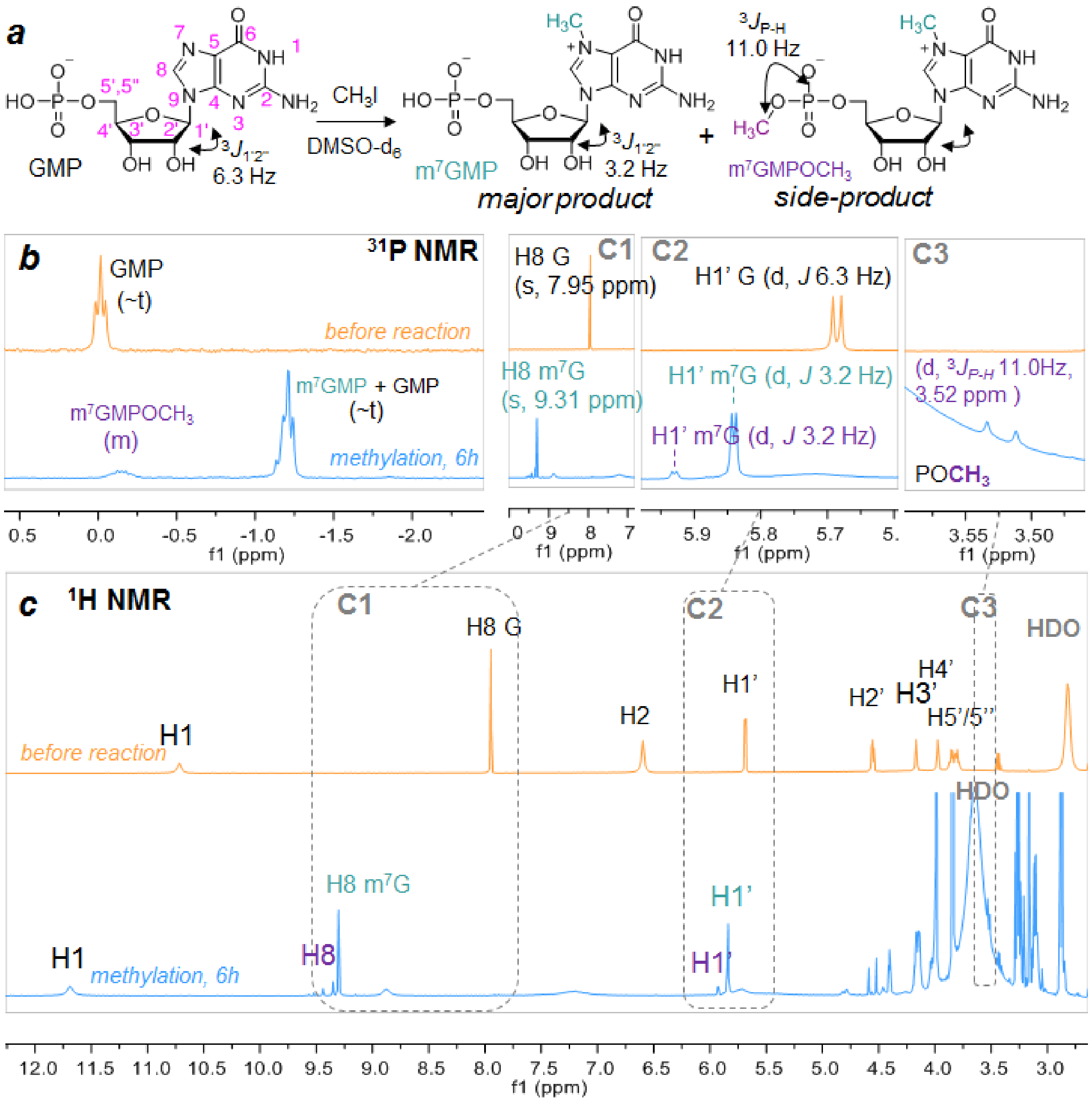

Fig. S10. **Analysis of GMP methylation reaction by ^{31}P and ^{1}H NMR.** A) Reaction scheme proposed based on HPLC and ESI (-) MS/MS analysis (Fig. 7, main manuscript); B) Analysis of reaction progress by ^{31}P NMR – indicated are signals crucial for structure confirmation; C) Analysis of reaction progress by ^{1}H NMR – indicated are signals crucial for structure confirmation.

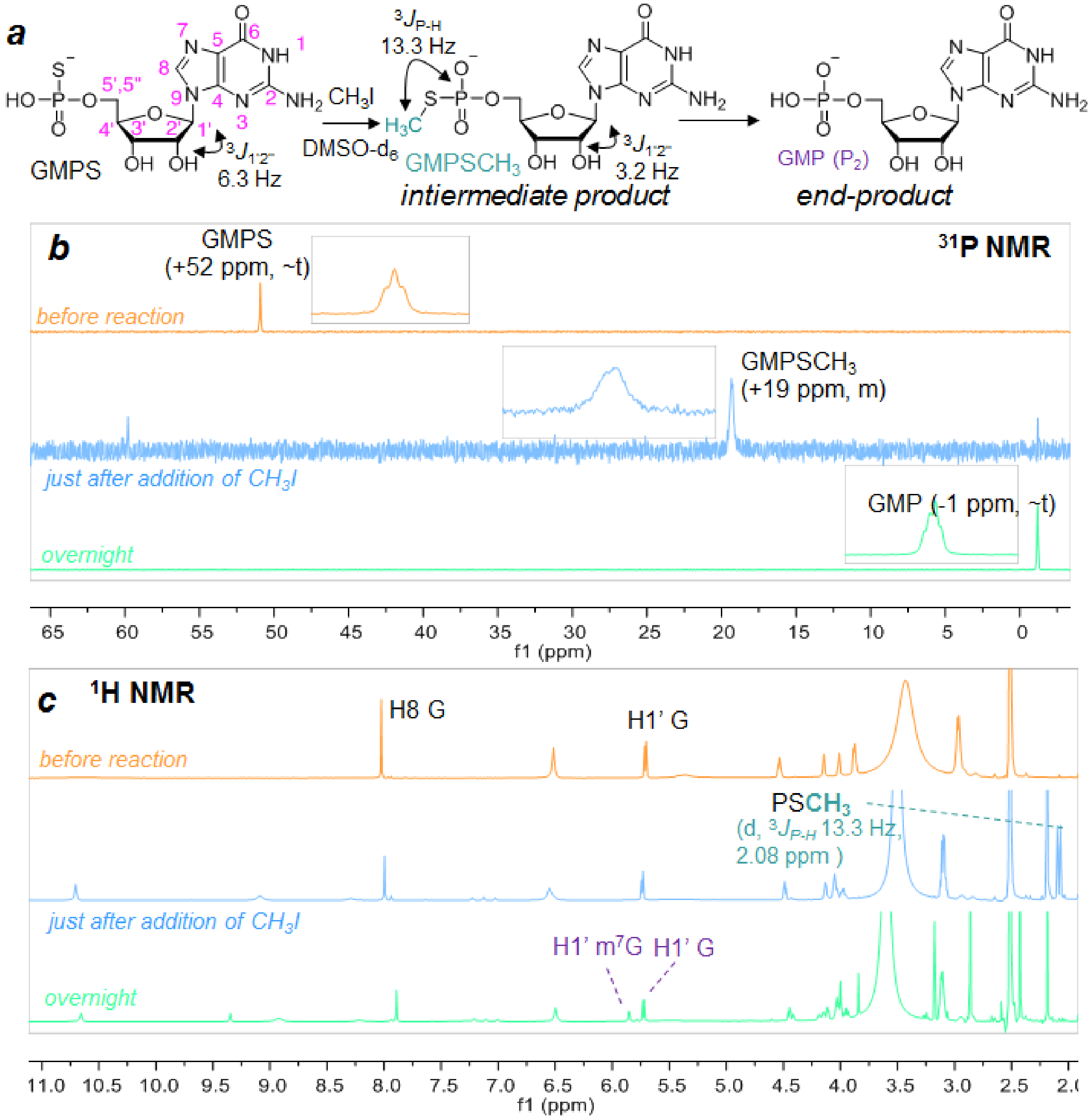

Fig. S11. **Analysis of GMPS methylation reaction by ^{31}P and ^{1}H NMR.** A) reaction scheme proposed based on HPLC and ESI (-) MS/MS analysis (Fig. 7, main manuscript); B) Analysis of reaction progress by ^{31}P NMR – indicated are signals crucial for structure confirmation; C) Analysis of reaction progress by ^{1}H NMR – indicated are signals crucial for structure confirmation.

Table S1. **Names and structures of investigated compounds.**

Nr	Abbreviation	Name	Structure	M	Flow rate µl/min	Concentration µM	Ref.[a]
1.	ddCMP	2′,3′-dideoxycytidine 5'-monophosphate	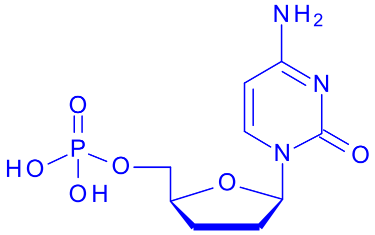	291	10	100	(YOSHIKAW. M et al., 1967)
2.	2'-dCMPF	2’-deoxycytidine 5′-fluoromonophosphate	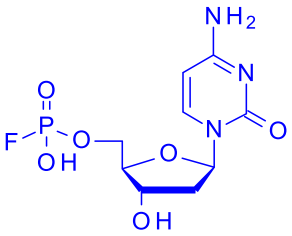	309	10	100	(Baranowski et al., 2015)
3.	CMP	Cytidine 5'-monophosphate	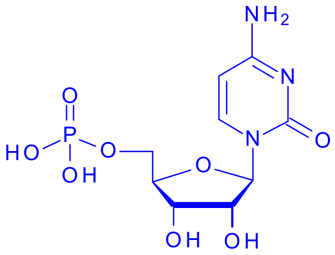	323	10	100	(YOSHIKAW. M et al., 1967)
4.	UMP	Uridine 5'-monophosphate	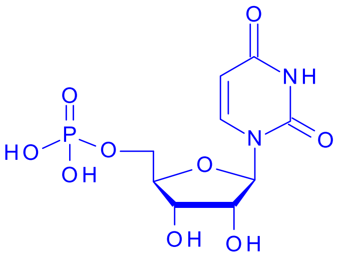	324	10	100	(YOSHIKAW. M et al., 1967)
5.	CMPF	Cytidine 5′-fluoromonophosphate	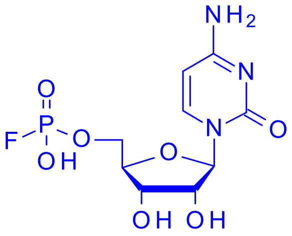	325	20	150	(Baranowski et al., 2015)
6.	UMPF	Uridine 5′-fluoromonophosphate	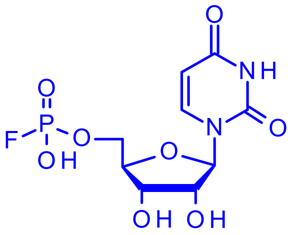	326	20	100	(Baranowski et al., 2015)

7.	3'-dAMP	3’-deoxyadenosine 5’-monophosphate	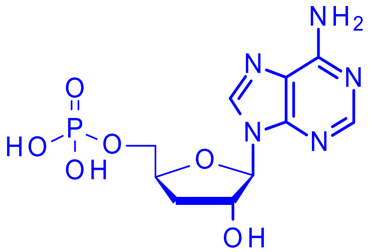	331	20	30	Commercial (Sigma Aldrich)
8.	2’-dAMP	2’-deoxyadenosine 5’-monophosphate	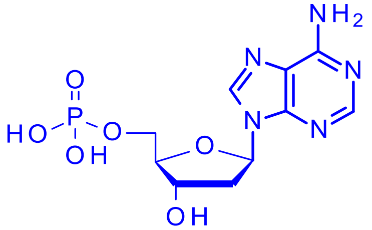	331	50	100	Commercial (Sigma Aldrich)
9.	$[^{18}O,^{18}O]$2’-dAMP	2’deoxyadenosine 5’-($[^{18}O,\ ^{18}O]$-monophosphate)	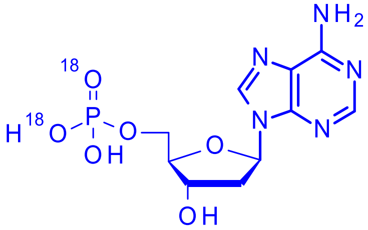	335	100	100	Experimental
10.	UMPS	Uridine 5′-thiomonophosphate	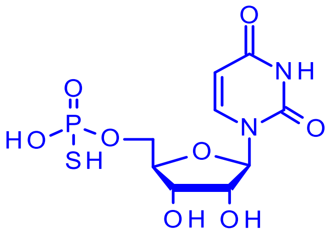	340	100	100	(Kowalska et al., 2008)
11.	AMPαBH_3	Adenosine 5′-boranomonophosphate	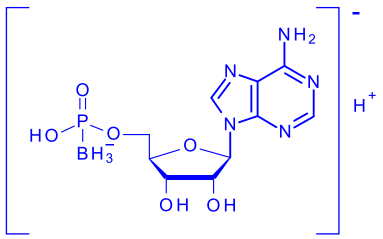	345	10	100	(Kowalska et al., 2014)
12.	$AMPNH_2$	Adenosine 5′-amidophosphate	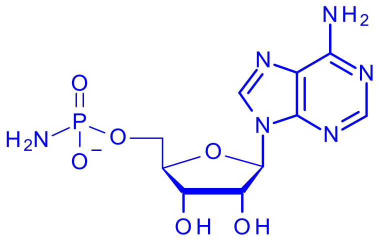	346	10	150	Experimental

13.	5’-AMP, AMP	Adenosine 5’-monophosphate	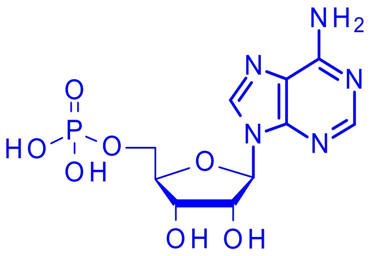	347	20	50	Commercial (Sigma-Aldrich)
14.	3'-AMP	Adenosine 3’-monophosphate	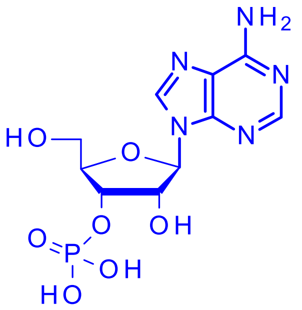	347	100	100	Commercial (Sigma Aldrich)
15.	AMPF αBH_3	Adenosine 5′-fluoroboranomonophosphate	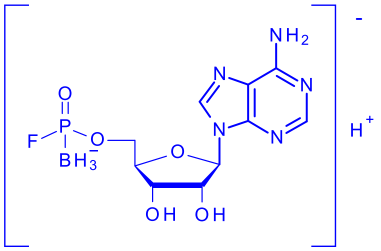	347	20	50	(Baranowski et al., 2015)
16.	GMPH	Guanosine 5’-H-phosphonate	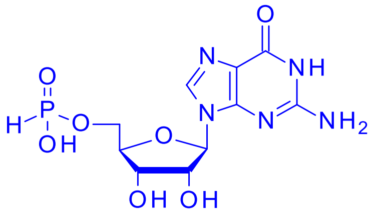	347	20	50	(Strenkowska et al., 2012)
17.	AZTMP	3′-azido-3′-deoxythymidine 5'-monophosphate	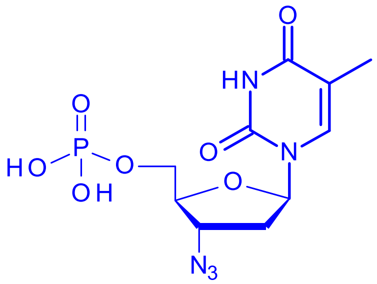	347	10	50	(YOSHIKAW. M et al., 1967)
18.	2'-F, 2’-dAMP	2’-fluoro-2’-deoxyadenosine 5’-monophosphate	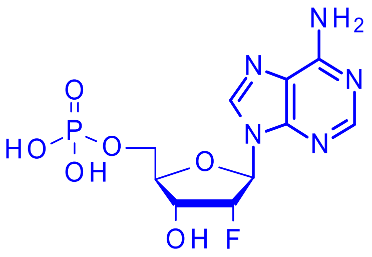	349	50	100	Experimental

19.	AMPF	Adenosine 5′-fluoromonophosphate	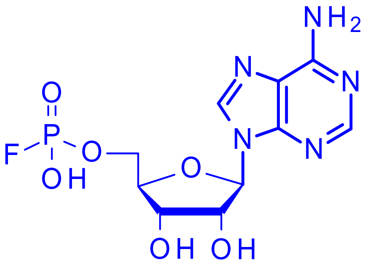	349	100	50	(Baranowski et al., 2015)
20.	[18O] AMP	Adenosine 5’-([18O]-monophosphate)	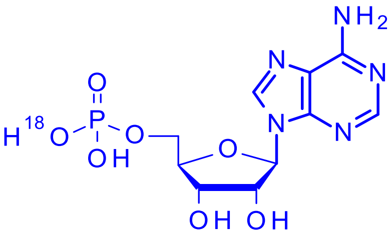	349	100	100	Experimental
21.	[18O,18O]AMP	Adenosine 5’- ([18O, 18O]-monophosphate)	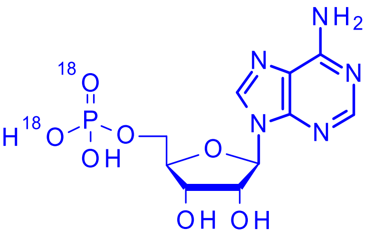	351	100	100	Experimental
22.	[18O,18O]2'-F, 2’-dAMP	2’-fluoro-2’-deoxyadenosine ([18O, 18O]-monophosphate)	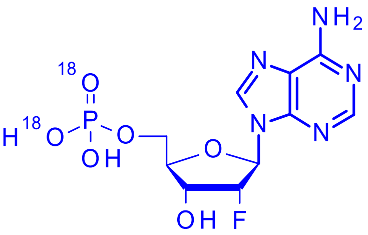	353	50	100	Experimental
23.	$GMP(NH_2)_2$	Guanosine 5′-phosphorodiamidate	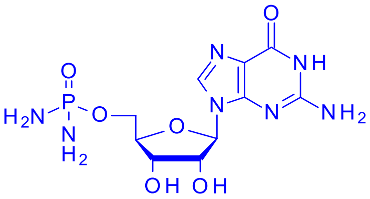	361	10	50	Experimental
24.	$GMPBH_3$	Guanosine 5′-boranophosphate	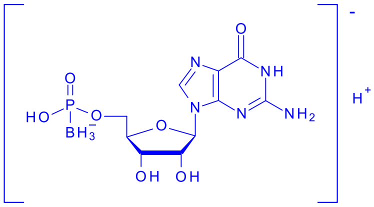	361	10	100	(Kowalska et al., 2014)
25.	$GMPNH_2$	Guanosine 5′-amidophosphate	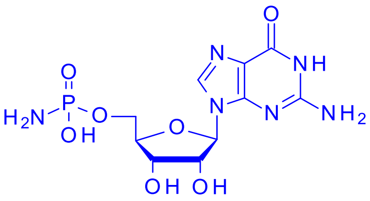	362	100	50	Experimental

26.	2’-NH_2, 2’-dGMP	2'-amino-2'-deoxyguanosine 5'-monophosphate	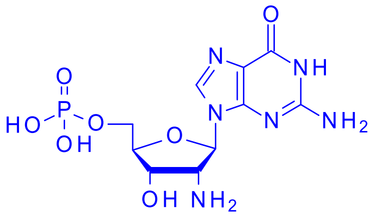	362	70	30	(Jemielity et al., 2012)
27.	3’NH2GMP	3'-amino-3'-deoxyguanosine 5'-monophosphate	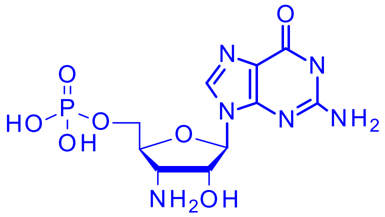	362	50	50	Experimental
28.	AMPS	Adenosine 5′-thiomonophosphate	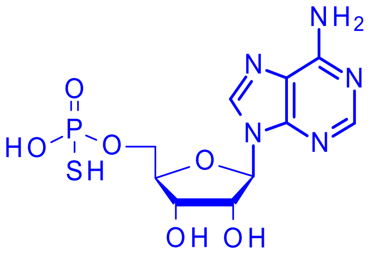	363	20	50	(Kowalska et al., 2008)
29.	GMP	Guanosine 5’-monophosphate	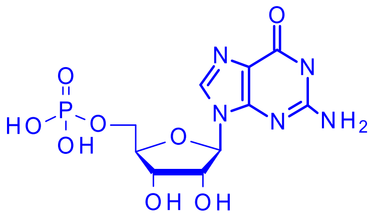	363	100	100	Commercial (Sigma-Aldrich)
30.	[^{18}O]AMPS	adenosine 5′-([^{18}O]thiomonophosphate)	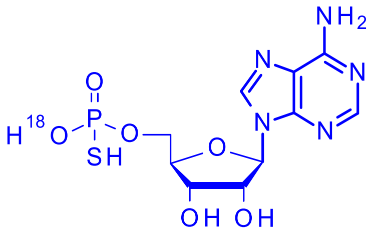	365	100	50	Experimental
31.	GMPF	Guanosine 5′-fluoromonophosphate	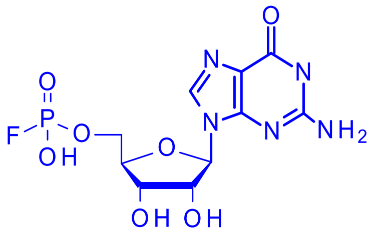	365	10	100	(Baranowski et al., 2015)

32.	[^{18}O,^{18}O]3'NH2 GMP	3'-amino-3'-deoxyguanosine 5'-([^{18}O,^{18}O]-monophosphate)	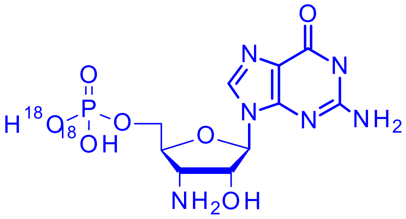	366	70	30	Experimental
33.	[[^{18}O,^{18}O] AMPS	Adenosine 5′-tiomonophosphate ^{18}O isotope	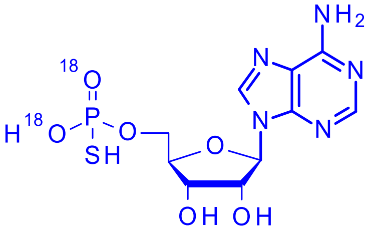	367	100	100	Experimental
34.	CO-GMPH	2’,3’-O,O-carbonyl-guanosine 5’-H-phosphonate	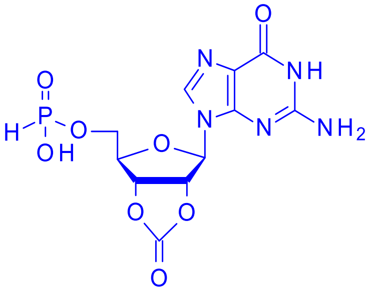	373	20	50	Experimental
35.	m^{7}GMPNH$_2$	*N*7-methylguanosine 5′-amidophosphate	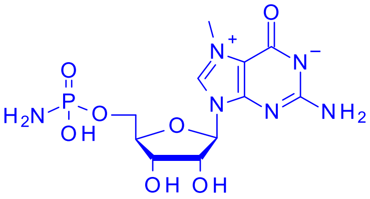	376	30	50	Experimental
36.	2'-NH$_2$, 2’-d m^{7}GMP	2'-amino-2'-deoxy-7-methylguanosine 5'-monophosphate	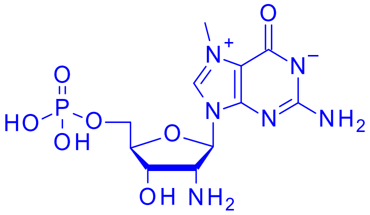	376	50	50	(Jemielity et al., 2012)
37.	m^{7}GMP	N7-methylguanosine 5′-monophosphate	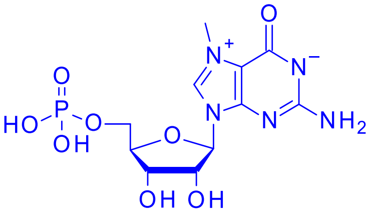	377	10	30	(Jemielity et al., 2003)

38.	$m^{2'-O}$GMP	2’-*O*-methylguanosine 5’-monophosphate	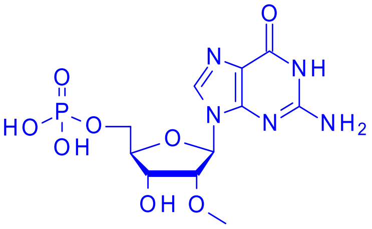	377	100	50	(Jemielity et al., 2003)
39.	3’-OMeGMP	3’-O-methylguanosine 5’-monophosphate	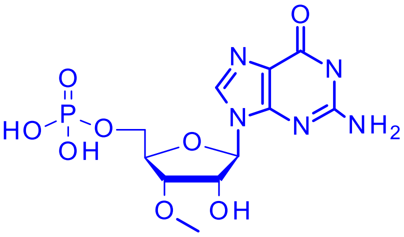	377	100	50	(Stepinski et al., 2001)
40.	GMPS	Guanosine 5′-thiomonophosphate	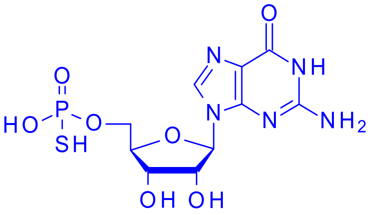	379	20	100	(Kowalska et al., 2008)
41.	GMSP	Guanosine 5’-phosphorothiolate	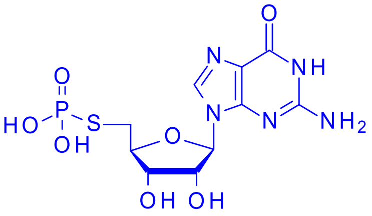	379	10	100	Wojtczak et. al. in preparation
42.	2'-dCDP	2’deoxycytidine 5′-diphosphate	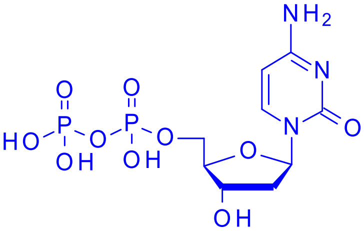	387	10	100	Commercial (Sigma Aldrich)
43.	$m_2^{7,2'-O}$GMP	*N*7,2’-O-dimethylguanosine	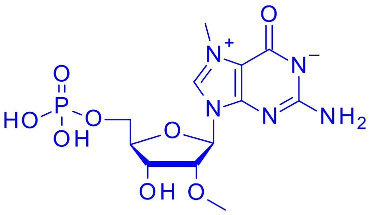	391	10	50	(Jemielity et al., 2003)
44.	m^7GMPOCH_3	*N*7-methylguanosine 5′-(*O*-methyl)monophosphate	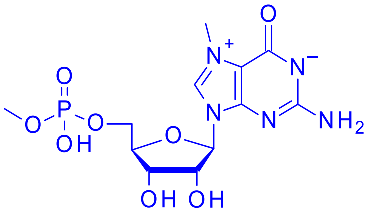	391	100	100	(Jemielity et al., 2003)

45.	$m^7GMPSNH_2$ D1	N7-methylguanosine 5'-phosphorothioamidate	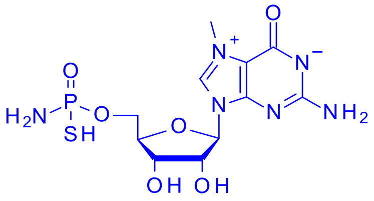	392	10	30	Kopcial et al. in preparation
46.	$GMPSCH_3$	Guanosine 5′-O-(S-methyl)thiomonophosphate	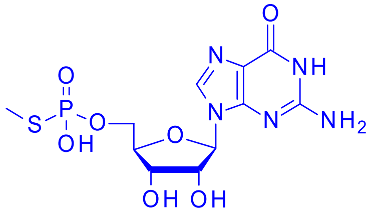	393	10	100	(Kowalska et al., 2009)
47.	m^7GMPS	*N*7-methylguanosine 5′-thiomonophosphate	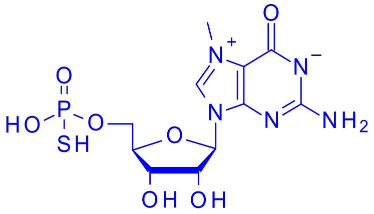	393	10	50	(Kowalska et al., 2008)
48.	$GMPNH_2CH_2CCH$	N-propargyl guanosine 5'-phosphoroamidate	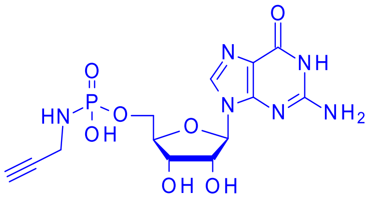	400	10	100	(Walczak et al., 2017)
49.	iPr-GMP	2',3'-*O,O*-isopropylideneguanosine 5'-monophosphate	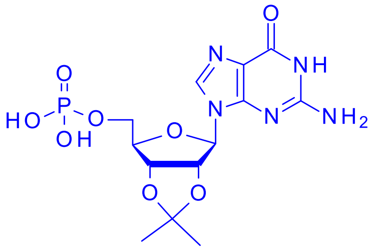	403	30	100	(Warminski et al., 2013)
50.	CDP	Cytidine 5′-diphosphate	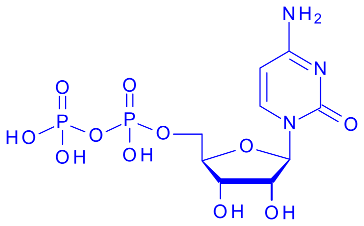	403	20	50	Commercial (Sigma Aldrich)
51.	UDP	Uridine 5′-diphosphate	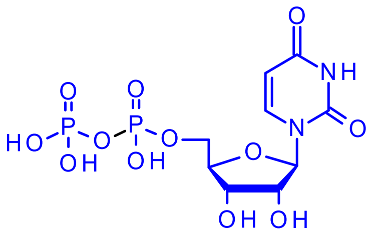	404	10	100	Commercial (Sigma Aldrich)

52.	m$^{2\ 7,2'-O}$GMPOCH$_3$	N7,2'-O-dimethylguanosine 5′-(O-methyl)monophosphate	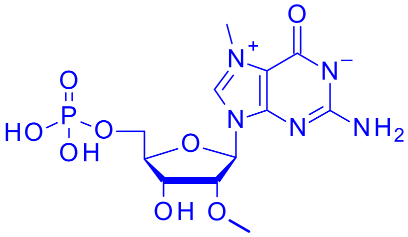	405	20	50	Experimental
53.	AMP-N6-etSH	N6-(2-mercaptoethyl)adenosine 5'-monophosphate	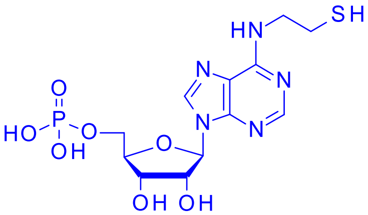	407	20	100	(Szczepaniak et al., 2012)
54.	m7GMPSCH3	N7-methylguanosine 5′-(S-methyl)monothiophosphate	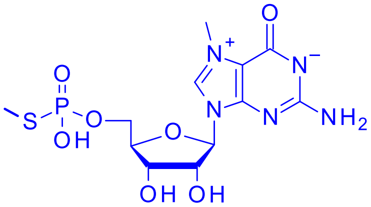	407	20	50	Experimental
55.	cPAP	2',3'-cyclophosphoadenosine 5'-phosphate	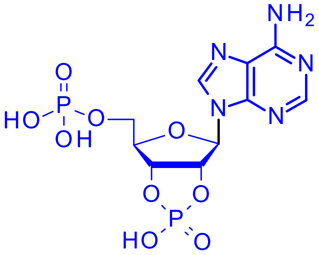	409	10	50	(Kowalska et al., 2012)
56.	2'-*O*-pentynyl AMP	2'-O- (5-pentynyl)adenosine 5'-monophosphate	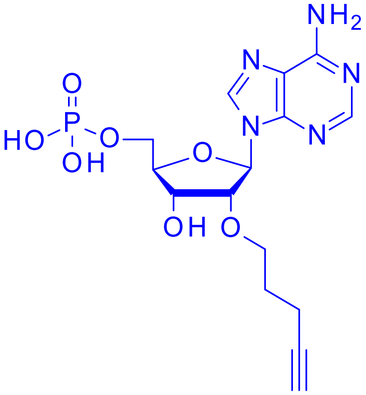	413	100	100	(Jawalekar et al., 2008)
57.	GMP-Im	Guanosine 5'-phosphorimidazolide	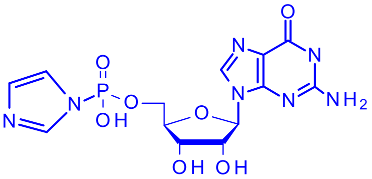	413	20	50	(Jemielity et al., 2003)

58.	$m^7GMPNH_2CH_2CCH$	N-propargyl N7-methylguanosine 5'-phosphoroamidate	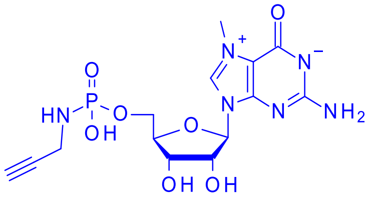	414	50	50	(Walczak et al., 2017)
59.	AMP-N6-BDA	N6-(4-aminobutyl)adenosine 5'-monophosphate	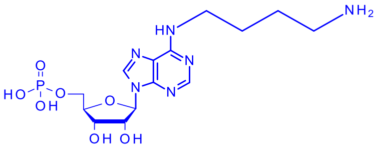	418	20	100	(Szczepaniak et al., 2012)
60.	$ADP\alpha BH_3$ D1	Adenosine 5'-O-(1-boranodiphosphate)	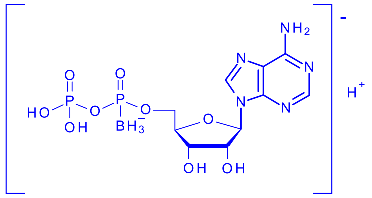	425	10	100	(Strenkowska et al., 2012)
61.	$ADP\alpha BH_3$ D2	Adenosine 5'-O-(1-boranodiphosphate)	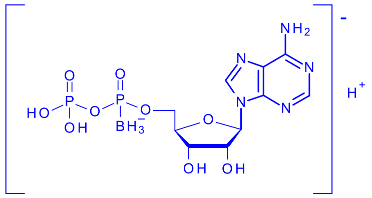	425	10	100	(Strenkowska et al., 2012)
62.	$ApCH_2p$	Adenosine 5'-O-methylenediphosphate	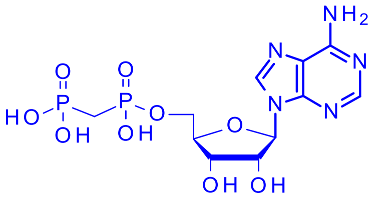	425	10	50	(Kalek et al., 2005)
63.	ApNHp	Adenosine 5'-O-imidodiphosphate	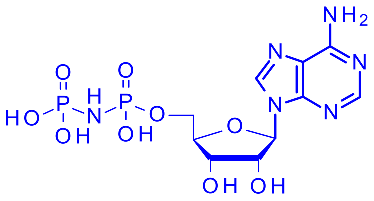	426	20	100	(Rydzik et al., 2012)
64.	m^7GMP-Im	N7-methylguanosine 5′-phosphorimidazolide	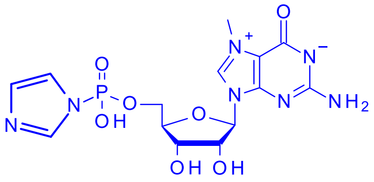	427	10	50	(Jemielity et al., 2003)
65.	ADP	Adenosine 5'-diphosphate	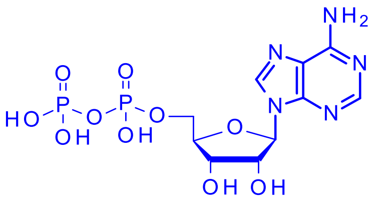	427	100	50	Commercial (Sigma Aldrich)

66.	APS	Adenosine 5'-phosphosulfate	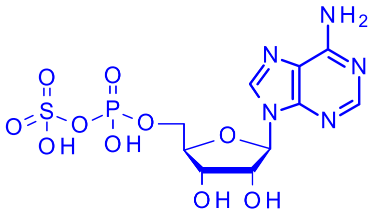	427	100	50	(Kowalska et al., 2012)
67.	ApCH2pF	Adenosine 5′-(2-Fluoro-1,2-methylenediphosphate)	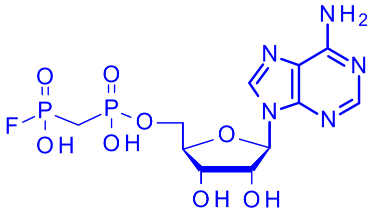	427	100	100	(Baranowski et al., 2015)
68.	2'-F 2'-dApCH$_2$p	2'-fluoro-2'-deoxyadenosine 5′-bisphosphonate	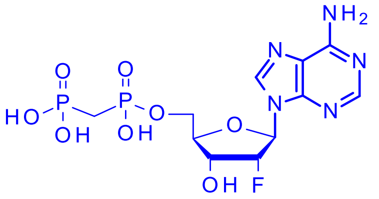	427	30	50	Experimental
69.	[^{18}O] ApCH2p	Adenosine 5'-O-(β[^{18}O]methylenediphosphate)	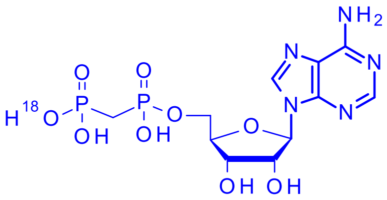	427	10	100	Experimental
70.	ADPF	Adenosine 5′-(2-fluorodiphosphate)	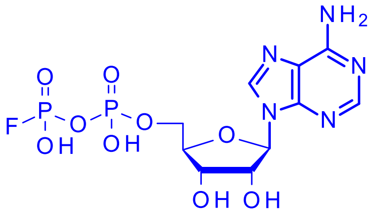	429	20	50	(Baranowski et al., 2015)
71.	2'-F-2'-d ADP	2'-fluoro-2'-deoxyadenosine 5′-(3-fluorodiphosphate)	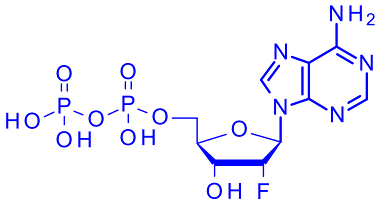	429	20	100	Experimental
72.	$GMPNHC_2H_4N_3$	N-(2-azdioethyl) guanosine 5'-phosphoroamidate	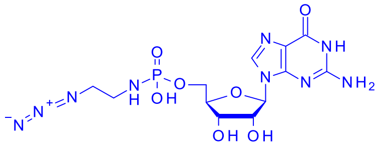	431	20	100	(Walczak et al., 2017)
73.	$GMPNH_2(CH_2)_4N_3$	N-(4-aminobutyl) guanosine 5'-phosphoroamidate	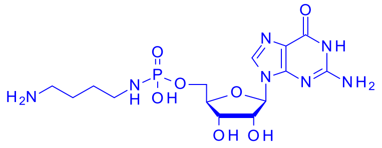	433	50	70	(Walczak et al., 2017)

74.	GpCH$_2$p	Guanosine 5’-O-methylenediphosphate	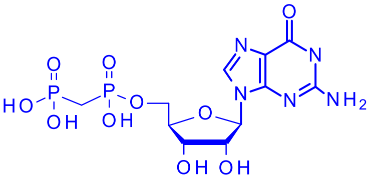	441	10	100	(Kalek et al., 2006)
75.	m^{7}GppH	7-methylguanosine 5’-diphosphate	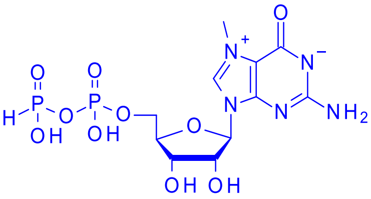	441	20	100	Experimental
76.	GpNHp	Guanosine 5’-O-imidodiphosphate	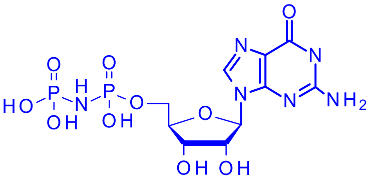	442	100	50	(TOMASZ et al., 1988)
77.	ADPαS	Adenosine 5’-(1-thiodiphosphate)	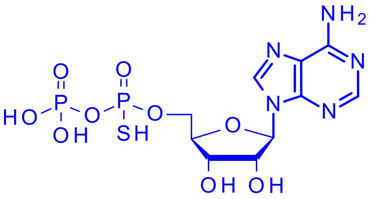	443	100	50	(Strenkowska et al., 2012)
78.	ADPβS	Adenosine 5’-(2-thiodiphosphate)	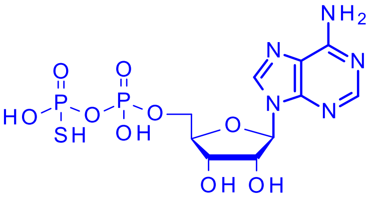	443	10	100	(Kowalska et al., 2007)
79.	GDP	Guanosine 5’-diphosphate	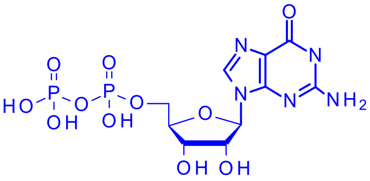	443	100	180	Commercial (Sigma Aldrich)
80.	GpBH$_3$pF_D1	Guanosine 5′-(2-Fluoro-1-boranodiphosphate)	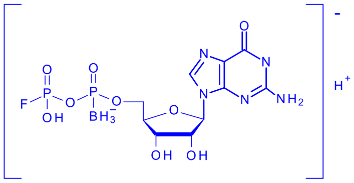	443	10	100	(Baranowski et al., 2015)
81.	GpBH$_3$pF_D2	Guanosine 5′-(2-Fluoro-1-boranodiphosphate)	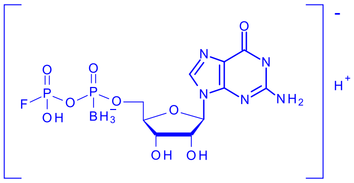	443	50	100	(Baranowski et al., 2015)

82.	GpCH2pF	Guanosine 5′-(2-Fluoro-1,2-methylenediphosphate)	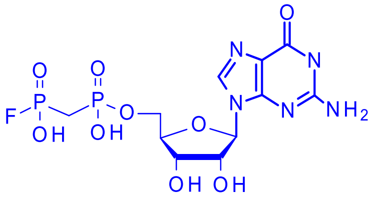	443	10	50	(Baranowski et al., 2015)
83.	dGDPS	2-deoxyguanosine 5’-(2-thiodiphosphate)	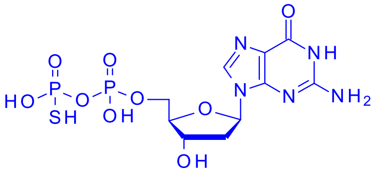	443	30	50	Experimental + (Kowalska et al., 2007)
84.	GpNHpF	Guanosine 5′-(2-Fluoro-1,2-imidodiphosphate)	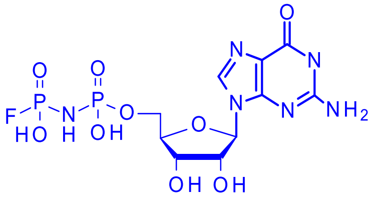	444	100	30	(Baranowski et al., 2015)
85.	GDPF	Guanosine 5′-Fluorodiphosphate	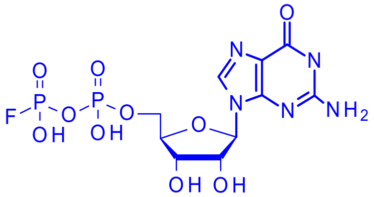	445	50	100	(Baranowski et al., 2015)
86.	$[^{18}O]$GDP	Guanosine 5′-(β$[^{18}O]$-diphosphate)	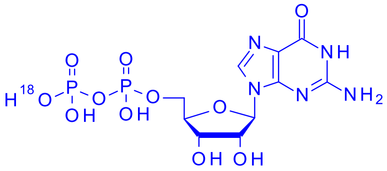	445	10	100	Experimental
87.	AMP-N6-HMDA	N6-(6-aminohexyl)adenosine 5'-monophosphate	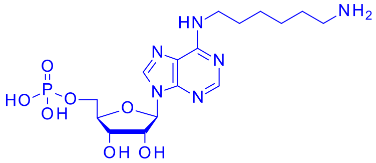	446	10	50	(Szczepaniak et al., 2012)
88.	GppC$_2$H	β-C-(2-ethynyl) guanosine diphosphate	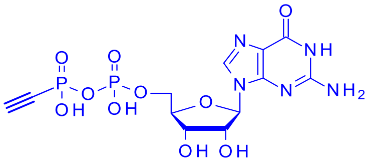	451	20	50	(Wanat et al., 2015)
89.	bn^{7}GMP	N7-benzylguanosine 5’-monophosphate	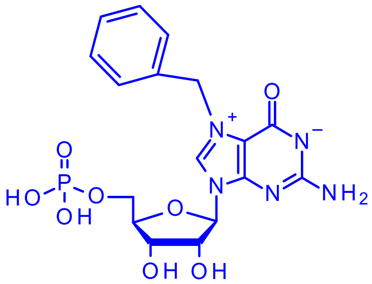	453	20	30	(Grudzien et al., 2004)

90.	$m^7GDP\alpha BH_3$	7-methylguanosine 5 - (1-boranodiphosphate)	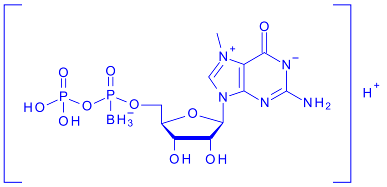	455	10	100	(Kowalska et al., 2014)
91.	m^7GpCH_2p	7-methylguanosine 5′-methylenebis(phosphonate)	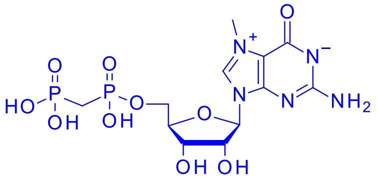	455	20	50	(Kalek et al., 2005)
92.	m^7GDP	7-methylguanosine 5’-diphosphate	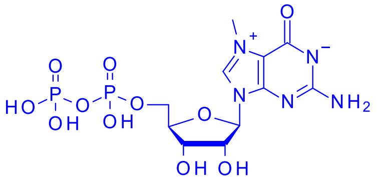	457	10	30	(Jemielity et al., 2003)
93.	m^7GPS	7-methylguanosine 5’-phosphosulfate	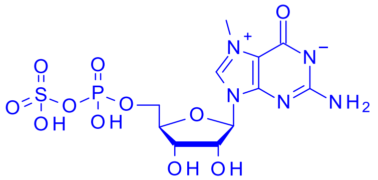	457	100	100	(Kowalska et al., 2012)
94.	$m^7GpNHpF$	7-methylguanosine 5′-(2-fluoro-1,2-imidodiphosphate)	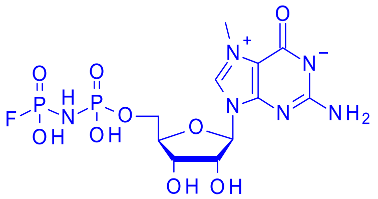	458	10	30	(Baranowski et al., 2015)
95.	cPAP-Im	2’,3’-cyclophosphoadenosine 5’-phosphorimidazolide	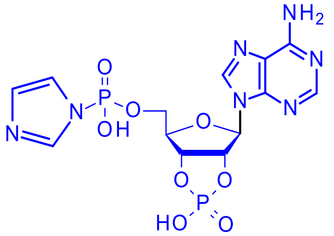	459	10	100	(Kowalska et al., 2012)
96.	GDPβS	Guanosine 5′-(2-thiodiphosphate)	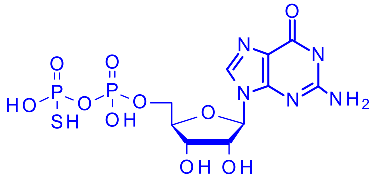	459	20	50	(Kowalska et al., 2007)
97.	m^7GDPF	7-methylguanosine 5′-fluorodiphosphate	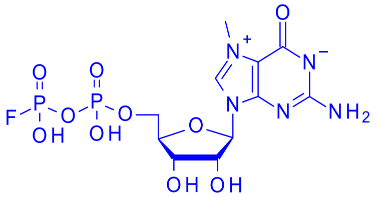	459	10	50	(Baranowski et al., 2015)

98.	GpSpF_D1	Guanosine 5′-(2-Fluoro-1-thiodiphosphate)	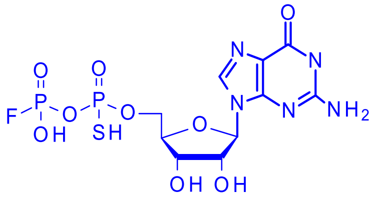	461	100	100	(Baranowski et al., 2015)
99.	GpSpF_D2	Guanosine 5′-(2-Fluoro-1-thiodiphosphate)	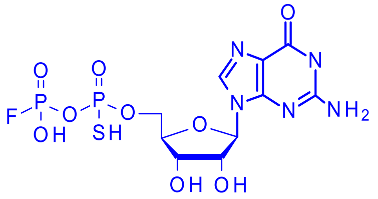	461	100	100	(Baranowski et al., 2015)
100	$GppC_3H_3$	β-C-(2-propargyl) guanosine diphosphate	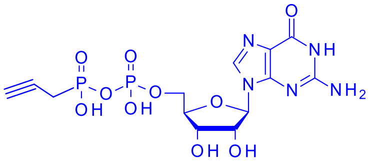	465	10	100	(Wanat et al., 2015)
101	2'-dCTP	2'deoxycytidine 5′-triphosphate	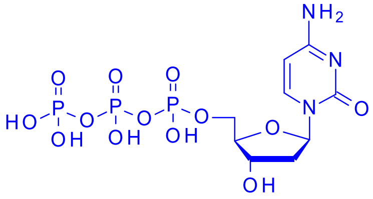	467	20	150	Commercial (Sigma Aldrich)
102	DMGpNHp	*N,N*-dimethylguanosine 5'-imidodiphosphate	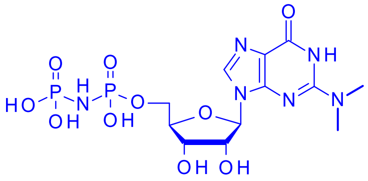	470	10	30	(Zytek et al., 2014)
103	2'-*O*-MCE-N_3 AMP-	2'-O-(*N*-(2-Azidoethyl)carbamoyl) methyladenosine 5'-monophosphate	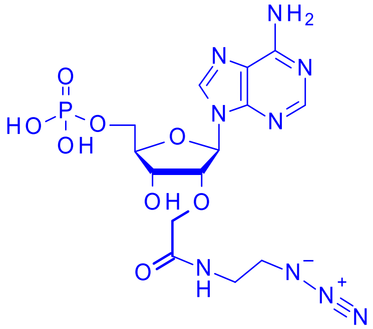	473	20	50	(Wojtczak et al., 2016)
104	m^7GDPαS D1	N 7 -methylguanosine 5'-O-(1-thiodiphosphate)	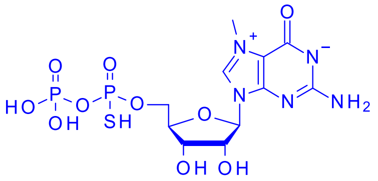	473	10	50	(Strenkowska et al., 2012)

105	m^{7}GDPαS D2	N 7 -methylguanosine 5'-O-(1-thiodiphosphate)	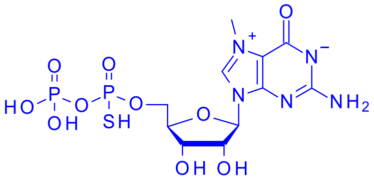	473	10	50	(Strenkowska et al., 2012)
106	ApBH$_3$pIm	Adenosine 5 -(1-borano-2-imidazoyl-diphosphate),	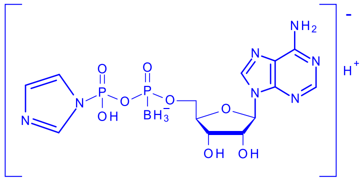	475	100	100	(Kowalska et al., 2014)
107	GppC$_4$H$_5$	guanosine 5'- [2-C-(3-butynyl)diphosphate]	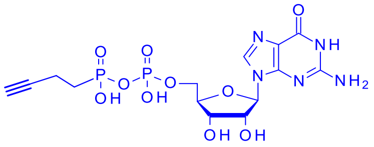	479	20	100	(Wanat et al., 2015)
108	m7GppC3H3	β-C-(2-propargyl) 7-methylguanosine diphosphate	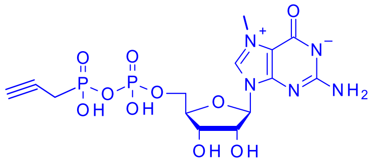	479	10	100	(Walczak et al., 2017)
109	AppEtCN	Adenosine 5′-[(2-O-cyanoethyl)diphosphate]	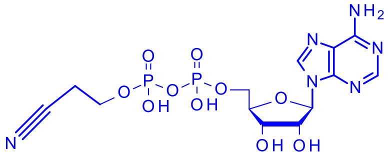	480	50	50	(Strenkowska et al., 2012)
110	GppNHC3H3	Guanosine 5′-(N2-propargyl-2-amino)diphosphate	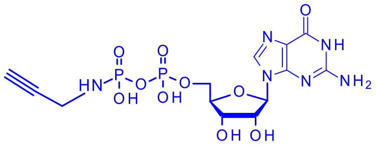	480	20	50	(Walczak et al., 2017)
111	GppOC3H3	Guanosine 5′-(2-propargyl)diphosphate	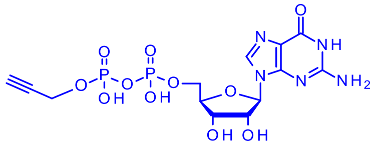	481	20	50	(Walczak et al., 2017)
112	AMP-NH-etS-S-etNH2	N6-cystamine-adenosine 5'-monophosphate	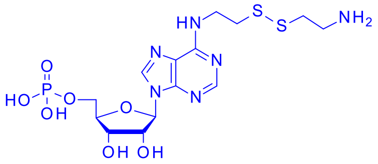	482	20	70	(Szczepaniak et al., 2012)
113	AMPIm-6-GABA	N6-(4-carboxybutyl)-adenosine 5'-monophosphate	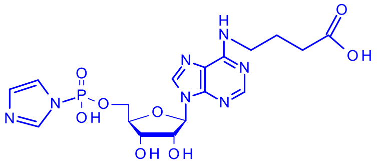	483	50	100	(Szczepaniak et al., 2012)

114	CTP	Cytidine 5'-triphosphate	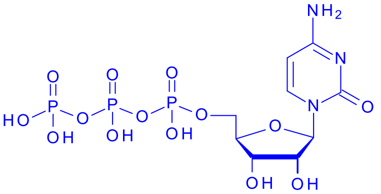	483	20	100	Commercial (Sigma Aldrich)
115	UTP	Uridine 5′-triphosphate	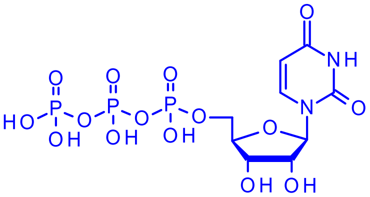	484	20	100	Commercial (Sigma Aldrich)
116	$m^{7,2}$GDPβS	N7,2'-O-dimethylguanosine 5′-(2-thiodiphosphate)	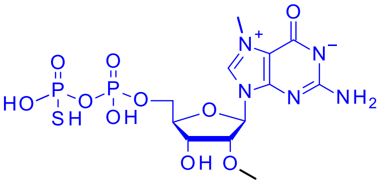	487	100	30	(Kowalska et al., 2007)
117	cPAPS	2’,3’-cyclophosphoadenosine 5’-phosphosulfate	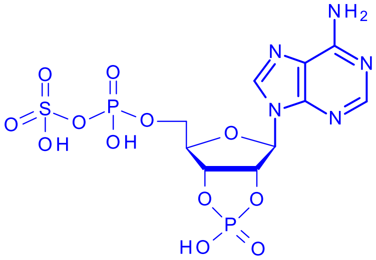	489	100	100	(Kowalska et al., 2012)
118	ADPβSe	Adenosine 5′-(2-selenodiphosphate)	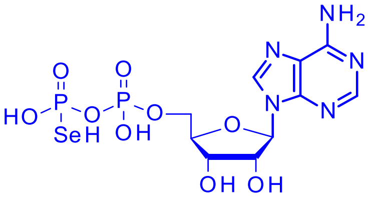	491	10	50	(Kowalska et al., 2009)
119	GpNHpIm	Guanosine 5′-(2-Imidazoyl-1,2-imidodiphosphate)	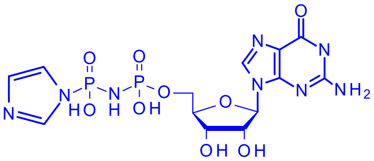	492	30	30	(Baranowski et al., 2015)
120	m7GppC4H5	β-C-(3-butynyl) 7-methylguanosine diphosphate	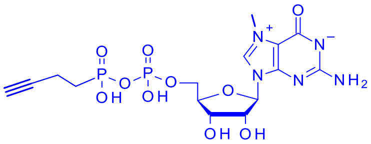	493	10	100	(Walczak et al., 2017)
121	m7GppNHC3H3	7-methylguanosine 5′-(N2-propargyl-2-amino)diphosphate	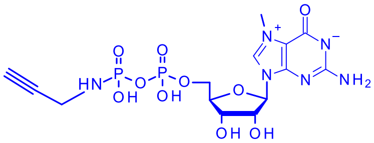	494	20	30	(Walczak et al., 2017)

122	GDP-EtCN	Guanosine 5′-[(2-O-cyanoethyl)diphosphate]	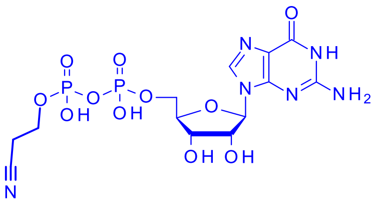	496	20	100	(Strenkowska et al., 2012)
123	ATPβBH3	Adenosine 5’-O-(2-boranotriphosphate)	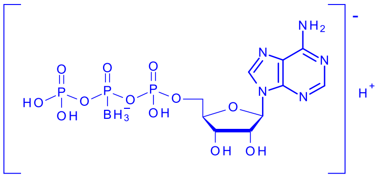	505	100	100	(Strenkowska et al., 2012)
124	ApCH2pp	Adenosine 5’-O-(1,2-methylenetriphoshate)	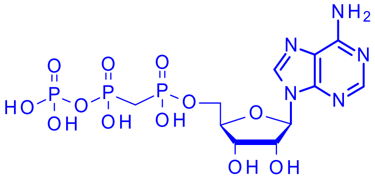	505	20	100	(Spelta et al., 2003)
125	AppCH2p	Adenosine 5’-O-(2,3-methylenetriphoshate)	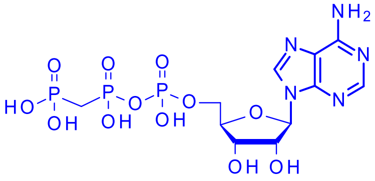	505	100	50	(Guranowski et al., 2006)
126	GDPβSe	Guanosine 5′-(2-selenodiphosphate)	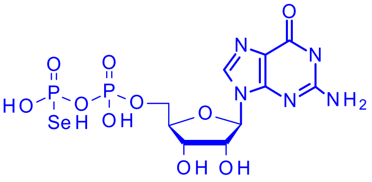	507	10	100	(Kowalska et al., 2009)
127	ATP	Adenosine 5’-triphosphate	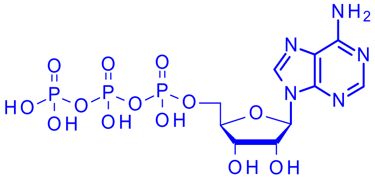	507	20	100	Commercial (Sigma Aldrich)
128	ApCH$_2$ppF	Adenosine 5′-(3-Fluoro-1,2-methylenetriphosphate)	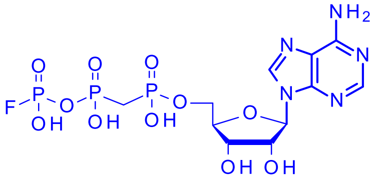	507	20	100	(Baranowski et al., 2015)
129	APPS	adenosine 5'-diphosphosulfate	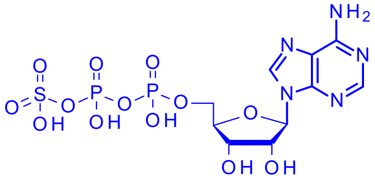	507	20	100	(Kowalska et al., 2012)

130	ATPF	Adenosine 5′-(3-Fluorotriphosphate)	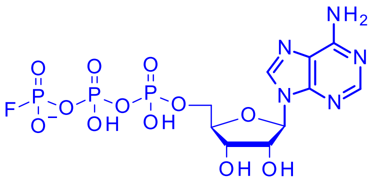	509	20	50	(Baranowski et al., 2015)
131	2’-F-2’-d ATP	2’-fluoro-2’-deoxyadenosine 5′-(3-Fluorotriphosphate)	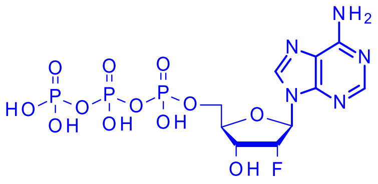	509	100	100	Experimental
132	cPADPF	2′,3′-Cyclic-phosphoadenosine 5′-(2-Fluorodiphosphate	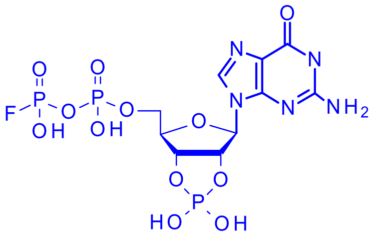	509	100	100	(Baranowski et al., 2015)
133	m7GMPTA	7-methylguanosine 5’-(tryptaminophosphate)	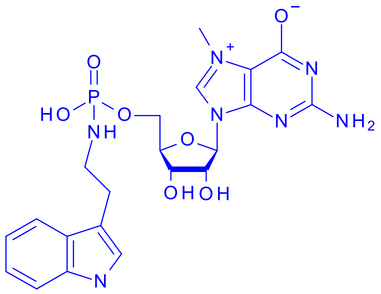	519	20	50	(Guranowski et al., 2011)
134	GTPβBH_3	Guanosine 5’-O-(2-boranotriphosphate)	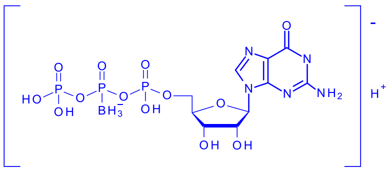	521	10	50	(Strenkowska et al., 2012)
135	GpCH_2pp	Guanosine 5’-O-(1,2-methylenetriphoshate)	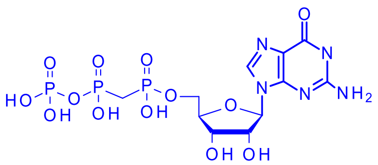	521	10	50	(Strenkowska et al., 2012)
136	GppCH_2p	Guanosine 5’-O-(2,3-methylenetriphoshate)	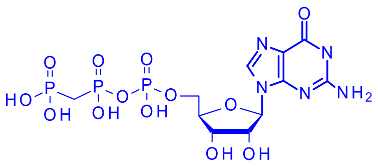	521	10	150	(Rydzik et al., 2009)

137	GTP	Guanosine 5′-triphosphate	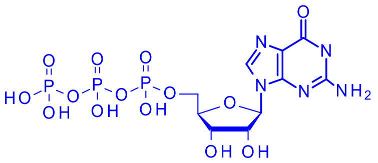	523	100	100	Commercial (Sigma Aldrich)
138	$GpCH_2ppF$	Guanosine 5′-(2-Fluoro-1,2-methylenediphosphate)	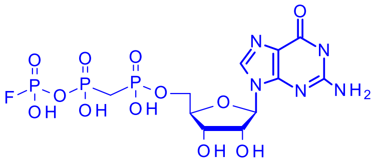	523	20	50	(Baranowski et al., 2015)
139	$GpBH_3ppF$	Guanosine 5′-(2-Fluoro-1-boranodiphosphate)	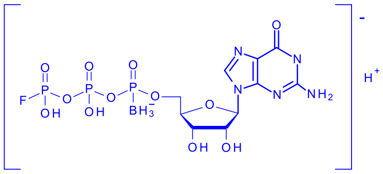	523	10	100	(Baranowski et al., 2015)
140	3’ (2’) Mant-GMP	2’-/3’-Methylanthraniloyl guanosine 5’-monophosphate	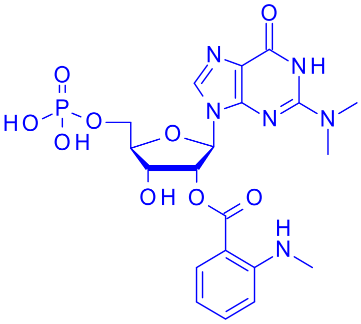	524	100	100	(Ziemniak et al., 2013)
141	GpNHppF	Guanosine 5′-(3-Fluoroimidotriphosphate)	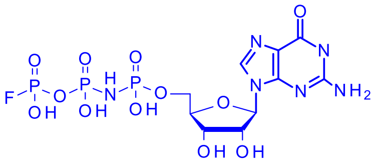	524	20	50	(Baranowski et al., 2015)
142	ApSppF	Adenosine 5′-(3-Fluoro-1-thiotriphosphate)	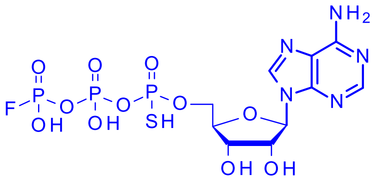	525	100	50	(Baranowski et al., 2015)
143	[^{18}O]GTP	Guanosine 5′-(γ[^{18}O]triphosphate)	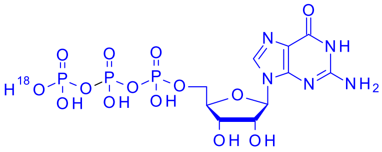	525	10	100	Experimental
144	m7GppCH2p	7-methylguanosine 5’-O-(2,3-methylenetriphoshate)	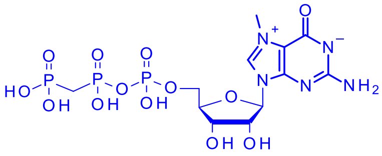	535	10	100	(Guranowski et al., 2006)

145	m^7GTP	7-methylguanosine 5'-triphosphate	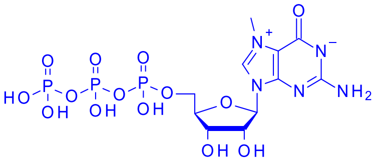	537	50	100	(Jemielity et al., 2003)
146	GpCH2ppS	Guanosine 5'-(1,2-methylene-3-thiotriphosphate)	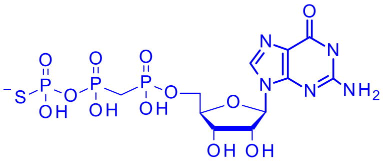	537	20	30	(Strenkowska et al., 2012)
147	GTPαS	Guanosine 5'-(1-thiotriphosphate)	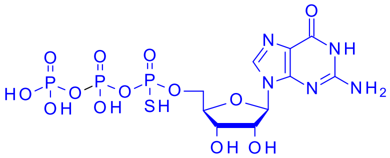	539	10	80	(Strenkowska et al., 2010)
148	GTPβS	Guanosine 5'-(2-thiotriphosphate)	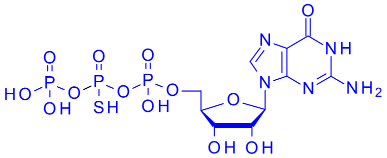	539	10	80	(Strenkowska et al., 2012)
149	GTPγS	Guanosine 5'-(3-thiotriphosphate)	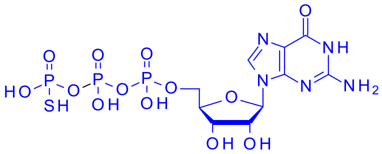	539	10	80	(Kowalska et al., 2007)
150	GpppC3H3	Guanosine 5'- [β-C-(2-propargyl)triphosphate]	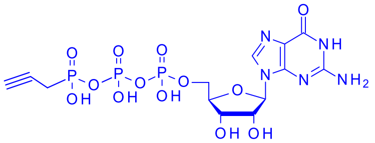	545	20	100	(Wanat et al., 2015)
151	AMP-N6-TTDA	13-amino-4,7,10-Trioxa-decane-N6-adenosine 5'-monophosphate	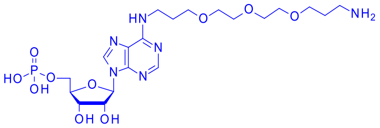	550	10	50	(Szczepaniak et al., 2012)
152	$m^7GTP\gamma S$	7-methylguanosine 5'-(3-thiotriphosphate)	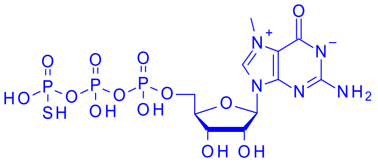	553	100	30	(Kowalska et al., 2007)
153	m7GTPαS_D1	7-methylguanosine 5'-(1-thiotriphosphate)	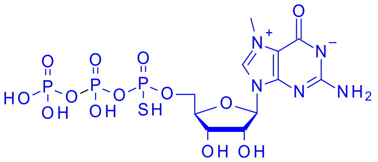	553	10	100	(Kowalska et al., 2009)
154	ADP-5-Rib	Adenosine Diphosphate 5-D-Ribofuranose	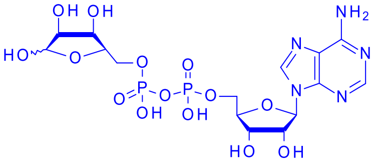	559	10	100	(Dabrowski-Tumanski et al., 2013)

155	GpppSC3H3	S-(2-propargyl) guanosine 5’-(3-thiotriphosphate)	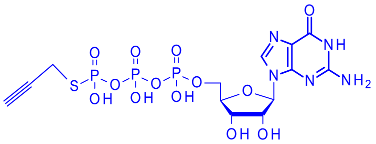	577	20	70	(Walczak et al., 2017)
156	Ant-m7 GDP	2’-/3’- Anthraniloyl 7-methylguanosine 5’-monophosphate	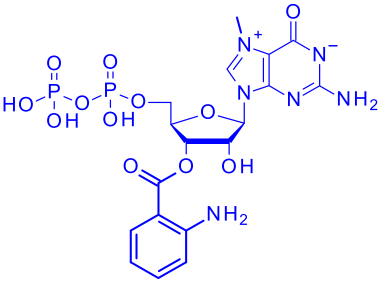	579	100	100	(Ziemniak et al., 2013)
157	GTPβSe	Guanosine 5’-O-(2-selenotriphosphate)	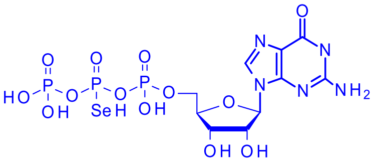	587	100	30	(Strenkowska et al., 2012)
158	ADP-6-Glc	Adenosine Diphosphate 6-D-Glucopyranose	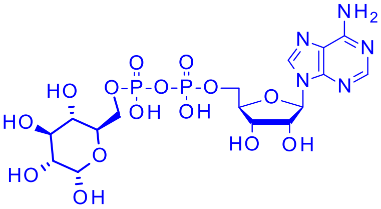	589	50	30	(Dabrowski-Tumanski et al., 2013)
159	GDP-1-β-Fuc	Guanosine Diphosphate 1-β-L-Fucopyranose	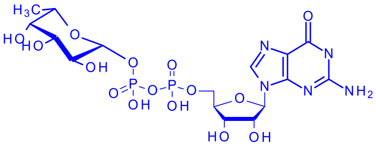	589	10	30	(Dabrowski-Tumanski et al., 2013)
160	GpCH2ppSEt CN	S-(2-cyanoethyl) guanosine 5’-(1,2-methylene-3-thio-triphosphate)	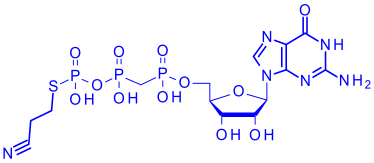	590	100	100	(Strenkowska et al., 2012)
161	$m^7GpppSC_3H_3$	S-(2-propargyl) 7-methylguanosine 5’-(3-thiotriphosphate)	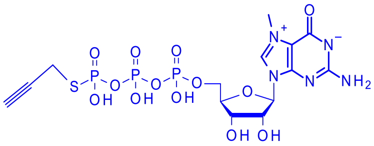	591	10	100	(Walczak et al., 2017)
162	$GpppNHC_2H_4N_3$	N-(2-propargyl) guanosine 5’-(3-aminotriphosphate)	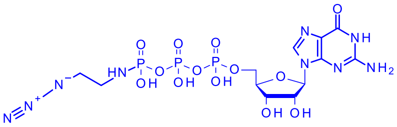	591	20	100	(Walczak et al., 2017)
163	m7GDPβTA	7-methylguanosine 5’-(2-tryptaminodiphosphate)	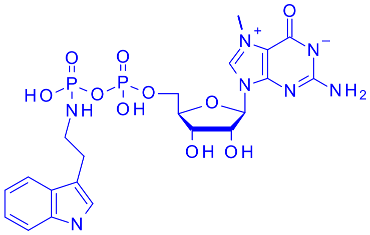	599	20	50	(Guranowski et al., 2011)

164	GpppCH2p	Guanosine 5’-O-(3,4-methylenetetraphoshate)	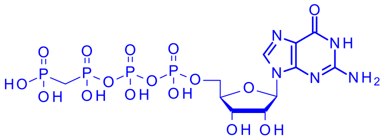	601	10	70	(Guranowski et al., 2006)
165	GDP-1-α-Glc	Guanosine 5-Diphosphate 1-α-D-Glucopyranose	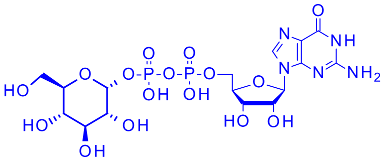	605	20	100	(Dabrowski-Tumanski et al., 2013)
166	AppSepEtCN	adenosine 5'-[2-seleno-3-(2-O-cyanoetylo)triphosphate]	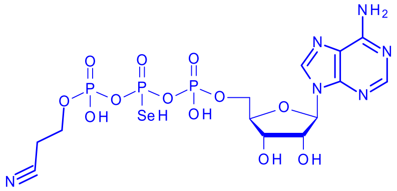	624	10	100	(Strenkowska et al., 2012)
167	GDPβS–AcPy	S-(1-acetylpyrenyl) guanosine 5’-(2-thiodiphosphate)	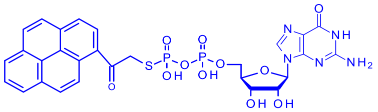	701	50	50	(Kasprzyk et al., 2016)
168	m^7 GDPβS–AcPy	S-(1-acetylpyrenyl) 7-methylguanosine 5’-(2-thiodiphosphate)	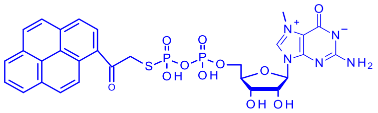	715	10	100	(Kasprzyk et al., 2016)

[a]Baranowski, M., A. Nowicka, A. Rydzik, M. Warminski, R. Kasprzyk, B. Wojtczak, J. Wojcik, T. Claridge, J. Kowalska, and J. Jemielity, 2015, Synthesis of Fluorophosphate Nucleotide Analogues and Their Characterization as Tools for F-19 NMR Studies: Journal of Organic Chemistry, v. 80, p. 3982-3997.

Dabrowski-Tumanski, P., J. Kowalska, and J. Jemielity, 2013, Efficient and Rapid Synthesis of Nucleoside Diphosphate Sugars from Nucleoside Phosphorimidazolides: European Journal of Organic Chemistry, p. 2147-2154.

Grudzien, E., J. Stepinski, M. Jankowska-Anyszka, R. Stolarski, E. Darzynkiewicz, and R. Rhoads, 2004, Novel cap analogs for in vitro synthesis of mRNAs with high translational efficiency: Rna-a Publication of the Rna Society, v. 10, p. 1479-1487.

Guranowski, A., E. Starzynska, M. Pietrowska-Borek, J. Jemielity, J. Kowalska, E. Darzynkiewicz, M. Thompson, and G. Blackburn, 2006, Methylene analogues of adenosine 5 '-tetraphosphate - Their chemical synthesis and recognition by human and plant mononucleoside tetraphosphatases and dinucleoside tetraphosphatases: Febs Journal, v. 273, p. 829-838.

Guranowski, A., A. Wojdyla, A. Rydzik, J. Stepinski, and J. Jemielity, 2011, Plant nucleoside 5 '-phosphoramidate hydrolase; simple purification from yellow lupin (Lupinus luteus) seeds and properties of homogeneous enzyme: Acta Biochimica Polonica, v. 58, p. 131-136.

Jawalekar, A., N. Meeuwenoord, J. Cremers, H. Overkleeft, G. van der Marel, F. Rutjes, and F. van Delft, 2008, Conjugation of nucleosides and oligonucleotides by [3+2] cycloaddition: Journal of Organic Chemistry, v. 73, p. 287-290.

Jemielity, J., M. Lukaszewicz, J. Kowalska, J. Czarnecki, J. Zuberek, and E. Darzynkiewicz, 2012, Synthesis of biotin labelled cap analogue - incorporable into mRNA transcripts and promoting cap-dependent translation: Organic & Biomolecular Chemistry, v. 10, p. 8570-8574.

Jemielity, J., J. Stepinski, M. Jaremko, D. Haber, R. Stolarski, R. Rhoads, and E. Darzynkiewicz, 2003, Synthesis of novel mRNA 5 ' cap-analogues: Dinucleoside P-1, P-3-tri-, P-1, P-4-tetra-, and P-1, P-5-pentaphosphates: Nucleosides Nucleotides & Nucleic Acids, v. 22, p. 691-694.

Kalek, M., J. Jemielity, Z. Darzynkiewicz, E. Bojarska, J. Stepinski, R. Stolarski, R. Davis, and E. Darzynkiewicz, 2006, Enzymatically stable 5 ' mRNA cap analogs: Synthesis and binding studies with human DcpS decapping enzyme: Bioorganic & Medicinal Chemistry, v. 14, p. 3223-3230.

Kalek, M., J. Jemielity, J. Stepinski, R. Stolarski, and E. Darzynkiewicz, 2005, A direct method for the synthesis of nucleoside 5'-methylenebis(phosphonate)s from nucleosides: Tetrahedron Letters, v. 46, p. 2417-2421.

Kasprzyk, R., J. Kowalska, Z. Wieczorek, M. Szabelski, R. Stolarski, and J. Jemielity, 2016, Acetylpyrene-labelled 7-methylguanine nucleotides: unusual fluorescence properties and application to decapping scavenger activity monitoring: Organic & Biomolecular Chemistry, v. 14, p. 3863-3868.

Kowalska, J., M. Lewdorowicz, E. Darzynkiewicz, and J. Jemielity, 2007, A simple and rapid synthesis of nucleotide analogues containing a phosphorothioate moiety at the terminal position of the phosphate chain: Tetrahedron Letters, v. 48, p. 5475-5479.

Kowalska, J., M. Lewdorowicz, J. Zuberek, E. Grudzien-Nogalska, E. Bojarska, J. Stepinski, R. E. Rhoads, E. Darzynkiewicz, R. E. Davis, and J. Jemielity, 2008, Synthesis and characterization of mRNA cap analogs containing phosphorothioate substitutions that bind tightly to eIF4E and are resistant to the decapping pyrophosphatase DcpS: RNA, v. 14, p. 1119-1131.

Kowalska, J., M. Lukaszewicz, J. Zuberek, M. Ziemniak, E. Darzynkiewicz, and J. Jemielity, 2009, Phosphorothioate analogs of m(7)GTP are enzymatically stable inhibitors of cap-dependent translation: Bioorganic & Medicinal Chemistry Letters, v. 19, p. 1921-1925.

Kowalska, J., A. Osowniak, J. Zuberek, and J. Jemielity, 2012, Synthesis of nucleoside phosphosulfates: Bioorganic & Medicinal Chemistry Letters, v. 22, p. 3661-3664.

Kowalska, J., A. Wypijewska del Nogal, Z. Darzynkiewicz, J. Buck, C. Nicola, A. Kuhn, M. Lukaszewicz, J. Zuberek, M. Strenkowska, M. Ziemniak, M. Maciejczyk, E. Bojarska, R. Rhoads, E. Darzynkiewicz, U. Sahin, and J. Jemielity, 2014, Synthesis, properties, and biological activity of boranophosphate analogs of the mRNA cap: versatile tools for manipulation of therapeutically relevant cap-dependent processes: Nucleic Acids Research, v. 42, p. 10245-10264.

Rydzik, A., M. Kulis, M. Lukaszewicz, J. Kowalska, J. Zuberek, Z. Darzynkiewicz, E. Darzynkiewicz, and J. Jemielity, 2012, Synthesis and properties of mRNA cap analogs containing imidodiphosphate moiety-fairly mimicking natural cap structure, yet resistant to enzymatic hydrolysis: Bioorganic & Medicinal Chemistry, v. 20, p. 1699-1710.

Rydzik, A., M. Lukaszewicz, J. Zuberek, J. Kowalska, Z. Darzynkiewicz, E. Darzynkiewicz, and J. Jemielity, 2009, Synthetic dinucleotide mRNA cap analogs with tetraphosphate 5 ',5 ' bridge containing methylenebis(phosphonate) modification: Organic & Biomolecular Chemistry, v. 7, p. 4763-4776.

Spelta, V., A. Mekhalfia, D. Rejman, M. Thompson, G. Blackburn, and R. North, 2003, ATP analogues with modified phosphate chains and their selectivity for rat P2X(2) and P2X(2/3) receptors: British Journal of Pharmacology, v. 140, p. 1027-1034.

Stepinski, J., C. Waddell, R. Stolarski, E. Darzynkiewicz, and R. Rhoads, 2001, Synthesis and properties of mRNAs containing the novel "anti-reverse" cap analogs 7-methyl(3 '-O-methyl)GpppG and 7-methyl(3 '-deoxy)GpppG: Rna-a Publication of the Rna Society, v. 7, p. 1486-1495.

Strenkowska, M., J. Kowalska, M. Lukaszewicz, J. Zuberek, W. Su, R. Rhoads, E. Darzynkiewicz, and J. Jemielity, 2010, Towards mRNA with superior translational activity: synthesis and properties of ARCA tetraphosphates with single phosphorothioate modifications: New Journal of Chemistry, v. 34, p. 993-1007.

Strenkowska, M., P. Wanat, M. Ziemniak, J. Jemielity, and J. Kowalska, 2012, Preparation of Synthetically Challenging Nucleotides Using Cyanoethyl P-Imidazolides and Microwaves: Organic Letters, v. 14, p. 4782-4785.

Szczepaniak, S., J. Zuberek, E. Darzynkiewicz, J. Kufel, and J. Jemielity, 2012, Affinity resins containing enzymatically resistant mRNA cap analogs-a new tool for the analysis of cap-binding proteins: Rna, v. 18, p. 1421-1432.

TOMASZ, J., M. VAGHEFI, P. RATSEP, R. WILLIS, and R. ROBINS, 1988, NUCLEOSIDE IMIDODIPHOSPHATES SYNTHESIS AND BIOLOGICAL-ACTIVITIES: Nucleic Acids Research, v. 16, p. 8645-8664.

Walczak, S., A. Nowicka, D. Kubacka, K. Fac, P. Wanat, S. Mroczek, J. Kowalska, and J. Jemielity, 2017, A novel route for preparing 5 ' cap mimics and capped RNAs: phosphate-modified cap analogues obtained via click chemistry: Chemical Science, v. 8, p. 260-267.

Wanat, P., S. Walczak, B. Wojtczak, M. Nowakowska, J. Jemielity, and J. Kowalska, 2015, Ethynyl, 2-Propynyl, and 3-Butynyl C-Phosphonate Analogues of Nucleoside Di- and Triphosphates: Synthesis and Reactivity in CuAAC: Organic Letters, v. 17, p. 3062-3065.

Warminski, M., J. Kowalska, J. Buck, J. Zuberek, M. Lukaszewicz, C. Nicola, A. Kuhn, U. Sahin, E. Darzynkiewicz, and J. Jemielity, 2013, The synthesis of isopropylidene mRNA cap analogs modified with phosphorothioate moiety and their evaluation as promoters of mRNA translation: Bioorganic & Medicinal Chemistry Letters, v. 23, p. 3753-3758.

Wojtczak, B., M. Warminski, J. Kowalska, M. Lukaszewicz, M. Honcharenko, C. Smith, R. Stromberg, E. Darzynkiewicz, and J. Jemielity, 2016, Clickable trimethylguanosine cap analogs modified within the triphosphate bridge: synthesis, conjugation to RNA and susceptibility to degradation: Rsc Advances, v. 6, p. 8326-8337.

YOSHIKAW.M, T. KATO, and TAKENISH.T, 1967, A NOVEL METHOD FOR PHOSPHORYLATION OF NUCLEOSIDES TO 5'-NUCLEOTIDES: Tetrahedron Letters, p. 5065-&.

Ziemniak, M., M. Szabelski, M. Lukaszewicz, A. Nowicka, E. Darzynkiewicz, R. Rhoads, Z. Wieczorek, and J. Jemielity, 2013, Synthesis and evaluation of fluorescent cap analogues for mRNA labelling: Rsc Advances, v. 3, p. 20943-20958.

Zytek, M., J. Kowalska, M. Lukaszewicz, B. Wojtczak, J. Zuberek, A. Ferenc-Mrozek, E. Darzynkiewicz, A. Niedzwiecka, and J. Jemielity, 2014, Towards novel efficient and stable nuclear import signals: synthesis and properties of trimethylguanosine cap analogs modified within the 5 ',5 '-triphosphate bridge: Organic & Biomolecular Chemistry, v. 12, p. 9184-9199.

Appendix A - AMP and ADP spectra on different equipment types

<table>
<tr><th>Equipment</th><th colspan="2">ADP
30 µM
(MetOH:H_2O 1:1, 5% NH_3)</th><th colspan="2">AMP
30 µM
(MetOH:H_2O 1:1, 5% NH_3)</th></tr>
<tr><td>QexactiveThermo</td><td rowspan="5">ESI negative ion mode</td><td rowspan="5">ESI positive ion mode</td><td rowspan="5">ESI negative ion mode</td><td rowspan="5">ESI positive ion mode</td></tr>
<tr><td>QTOF Waters</td></tr>
<tr><td>QTRAP 3200 Sciex Q1</td></tr>
<tr><td>QTRAP 3200 Sciex EPI (Enhance Product Ion)</td></tr>
<tr><td>API 3200 AB Sciex Q1</td></tr>
</table>

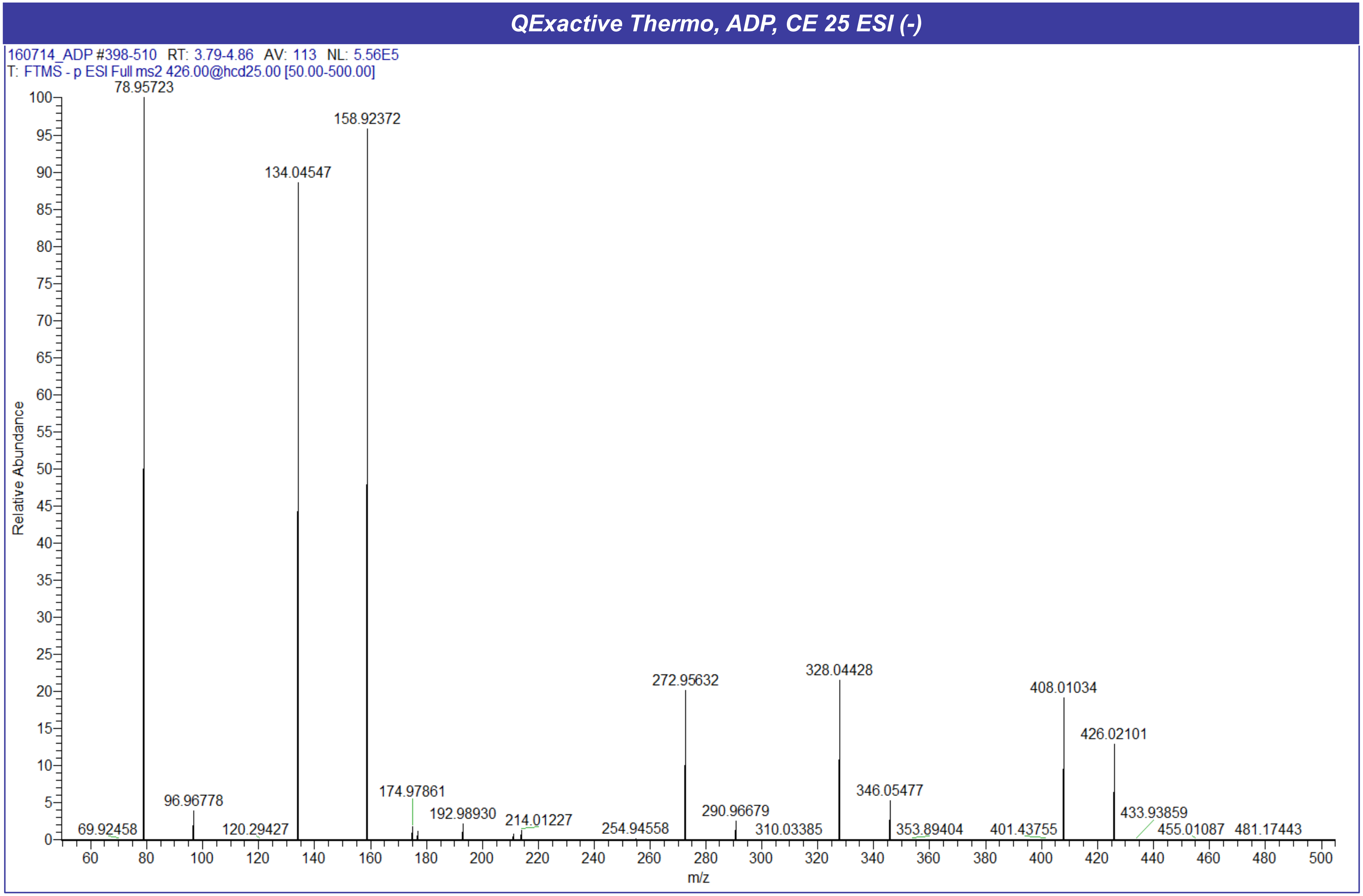
QExactive Thermo, ADP, CE 25 ESI (-)
160714_ADP #398-510 RT: 3.79-4.86 AV: 113 NL: 5.56E5
T: FTMS - p ESI Full ms2 426.00@hcd25.00 [50.00-500.00]
Relative Abundance
m/z
78.95723
158.92372
134.04547
272.95632
328.04428
408.01034
426.02101
69.92458
96.96778
120.29427
174.97861
192.98930
214.01227
254.94558
290.96679
310.03385
346.05477
353.89404
401.43755
433.93859
455.01087
481.17443

QTOF Waters, ADP, CE 20 ESI (-)

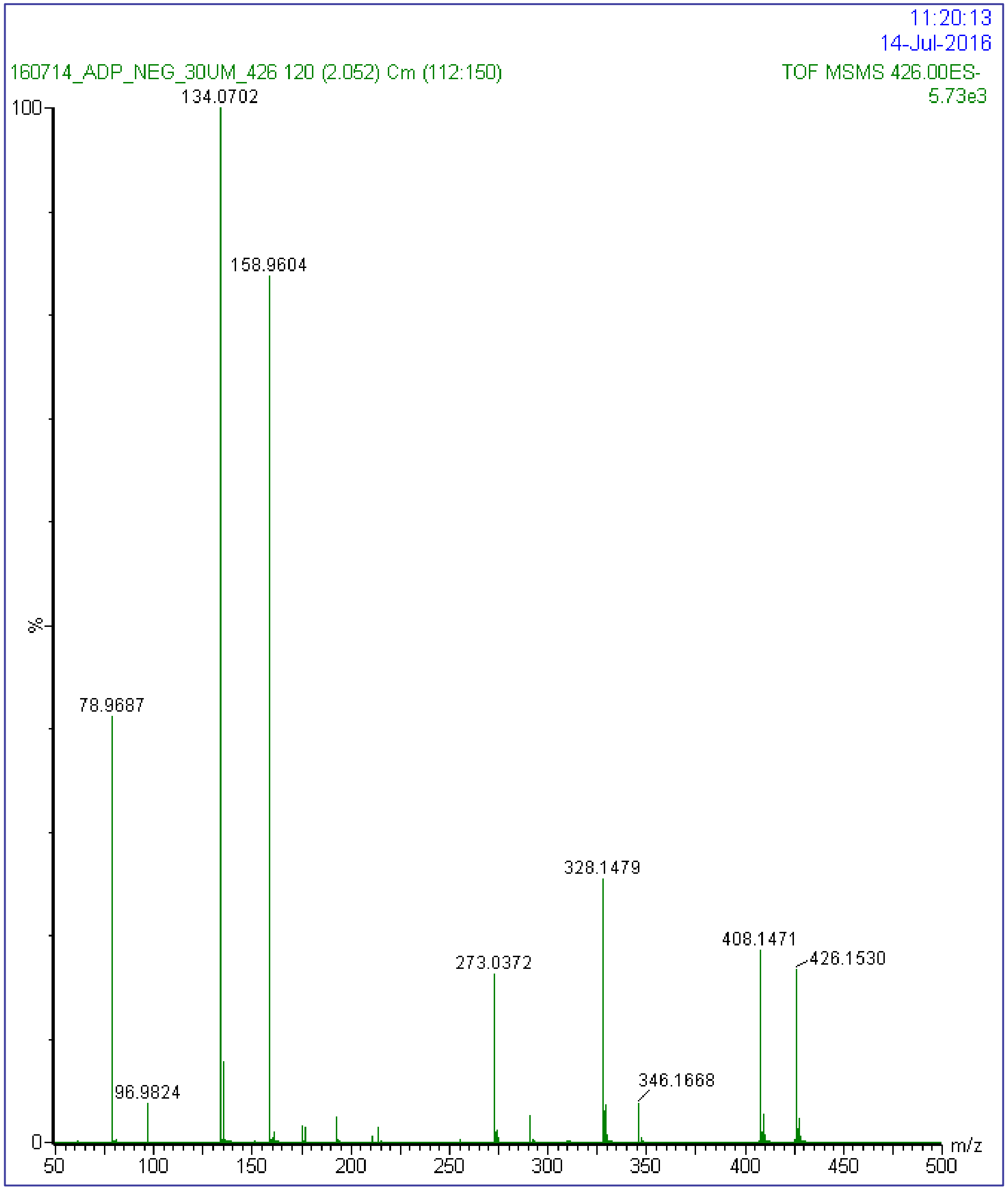

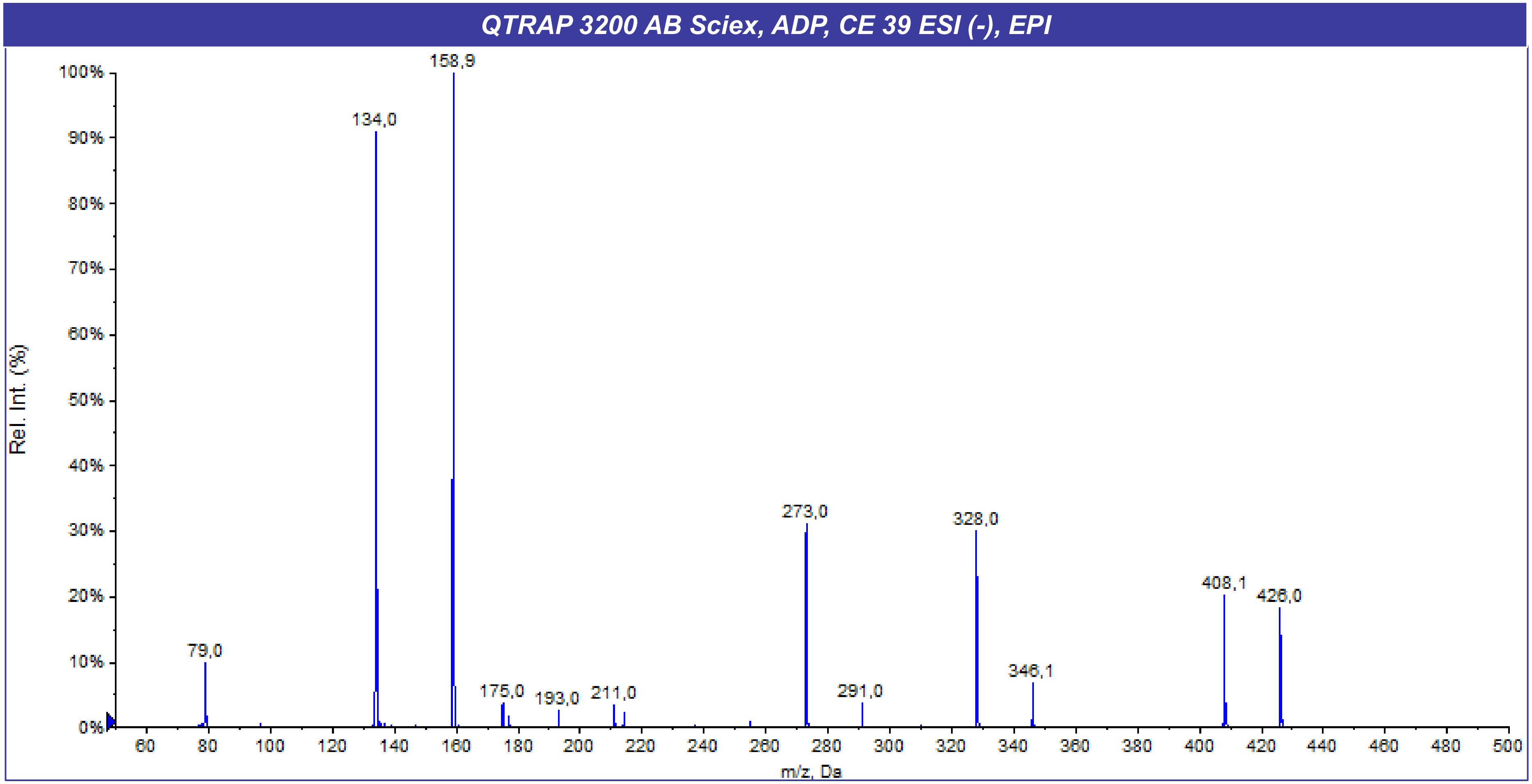

QTRAP 3200 AB Sciex, ADP, CE 39 ESI (-), EPI
Rel. Int. (%)
100%
90%
80%
70%
60%
50%
40%
30%
20%
10%
0%
158,9
134,0
79,0
175,0
193,0
211,0
273,0
291,0
328,0
346,1
408,1
426,0
60
80
100
120
140
160
180
200
220
240
260
280
300
320
340
360
380
400
420
440
460
480
500
m/z, Da

QTRAP 3200 AB Sciex, ADP, CE 40 ESI (-), Q1/Q3

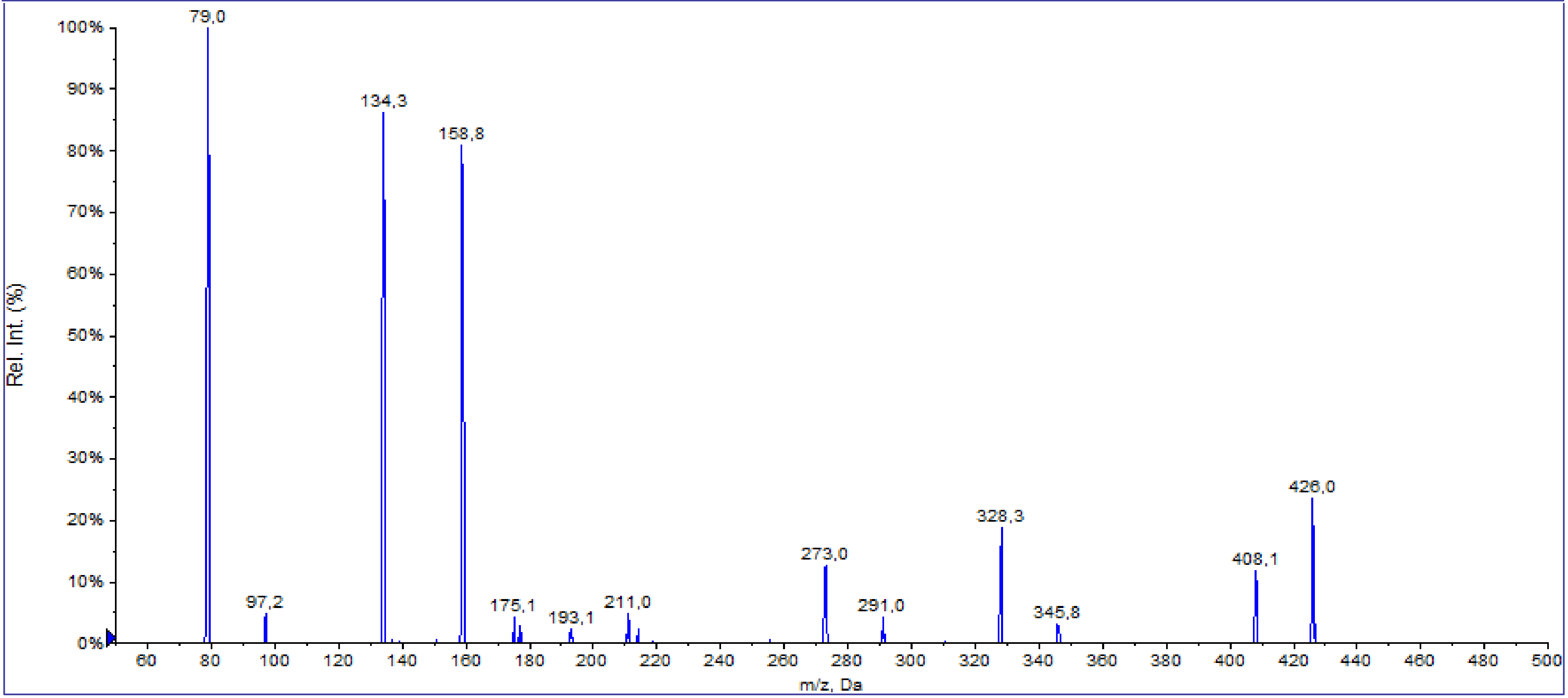

API 3200 AB Sciex, ADP, CE 45 ESI (-)

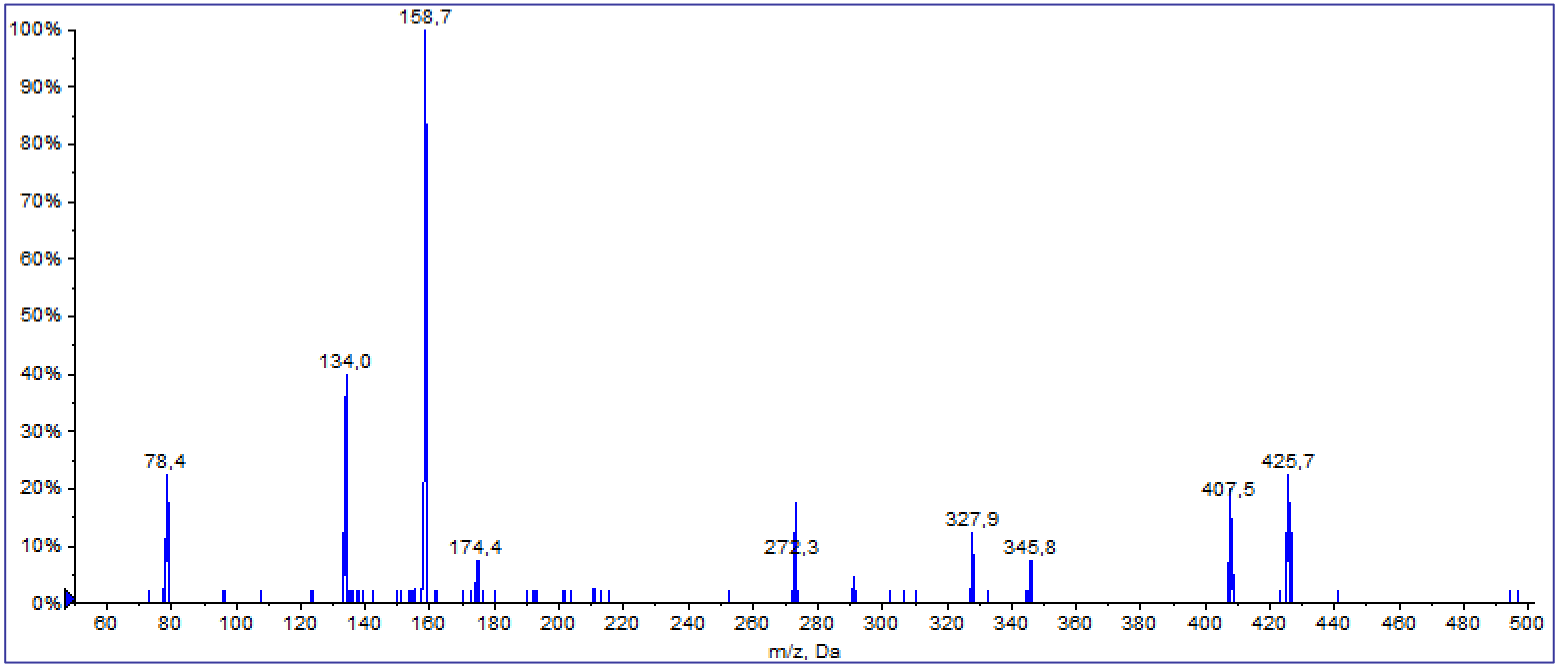

QExactive Thermo, ADP, CE 16 ESI (+)

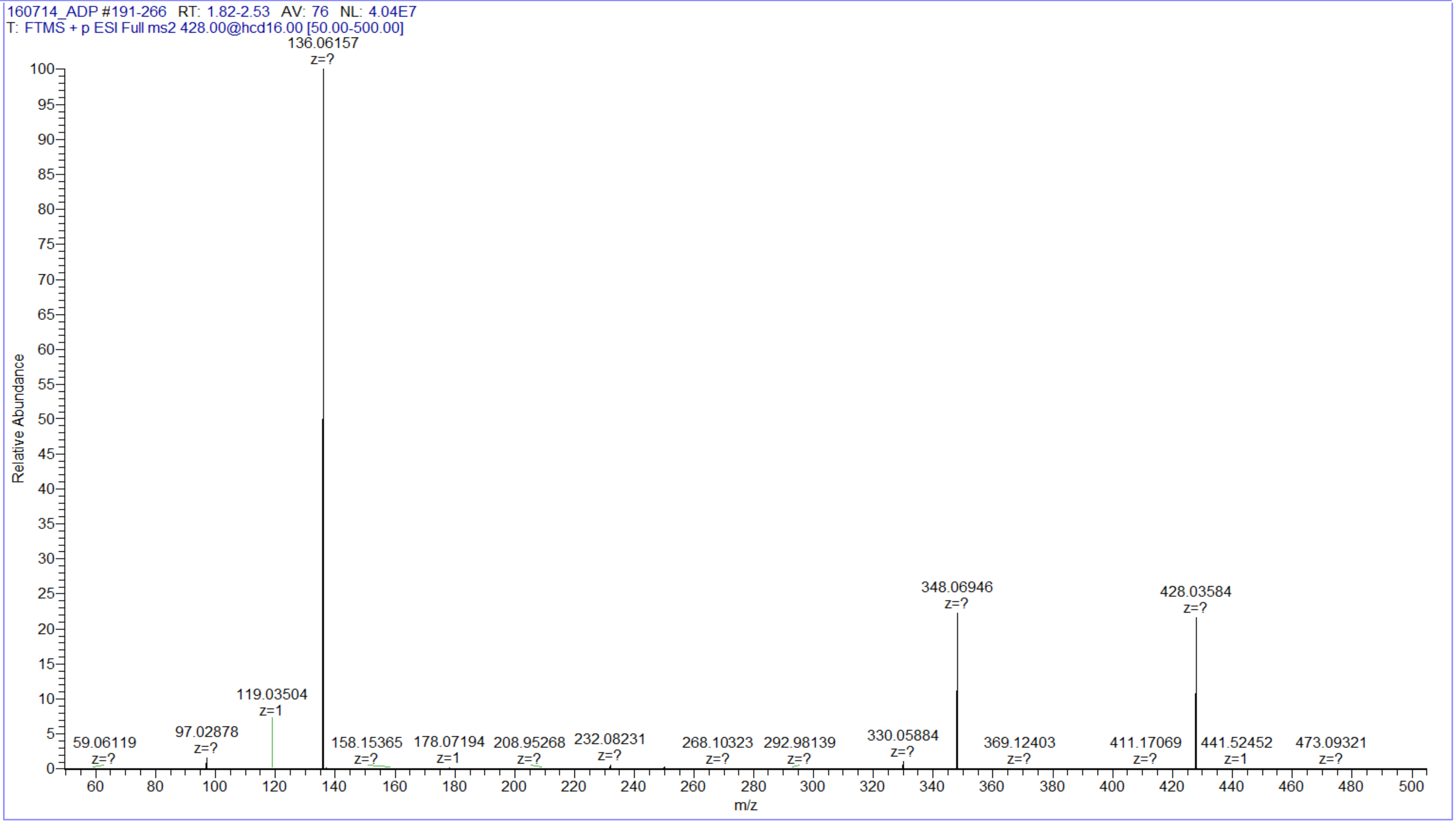

QTOF Waters, ADP, CE 13 ESI (+)

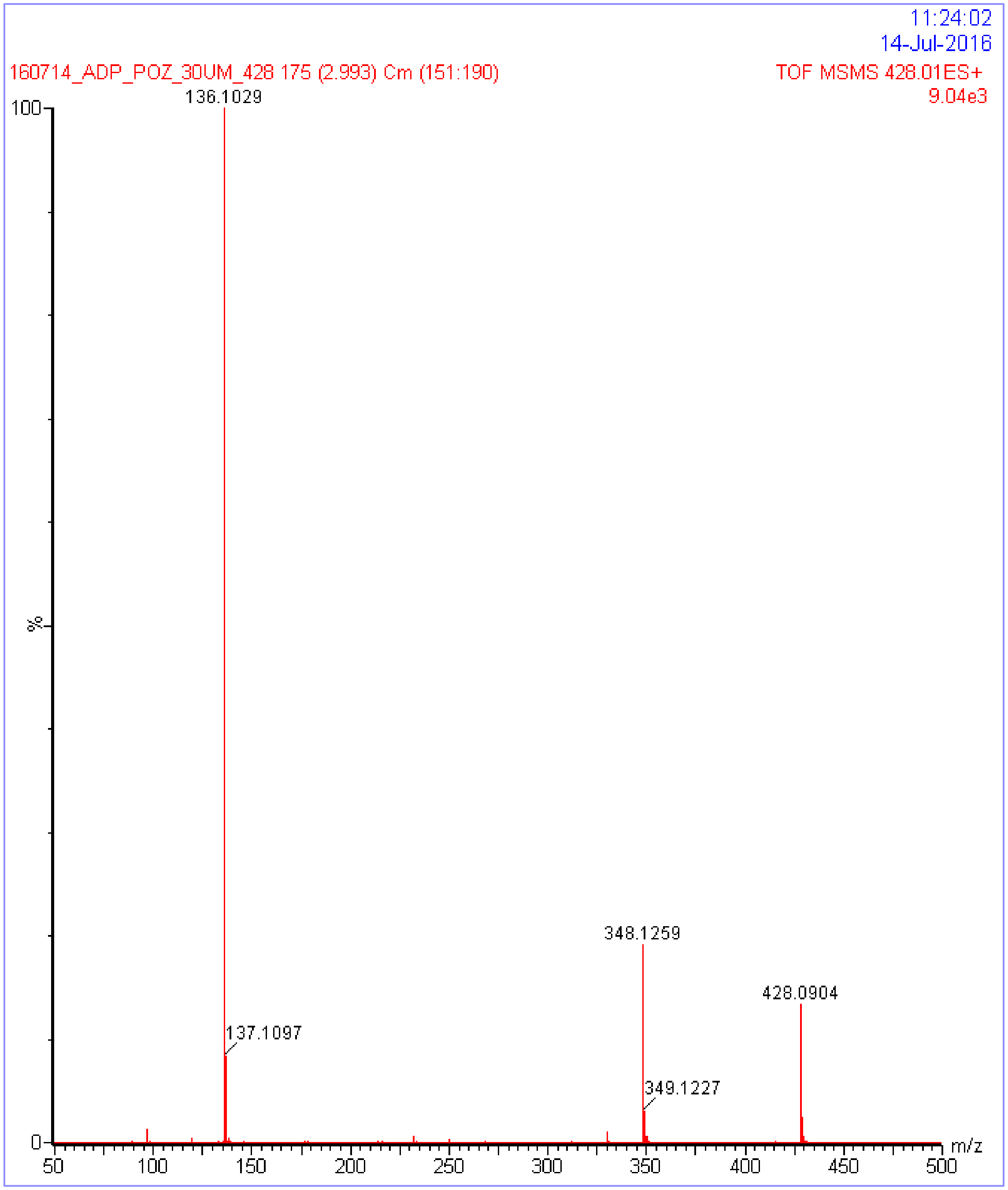

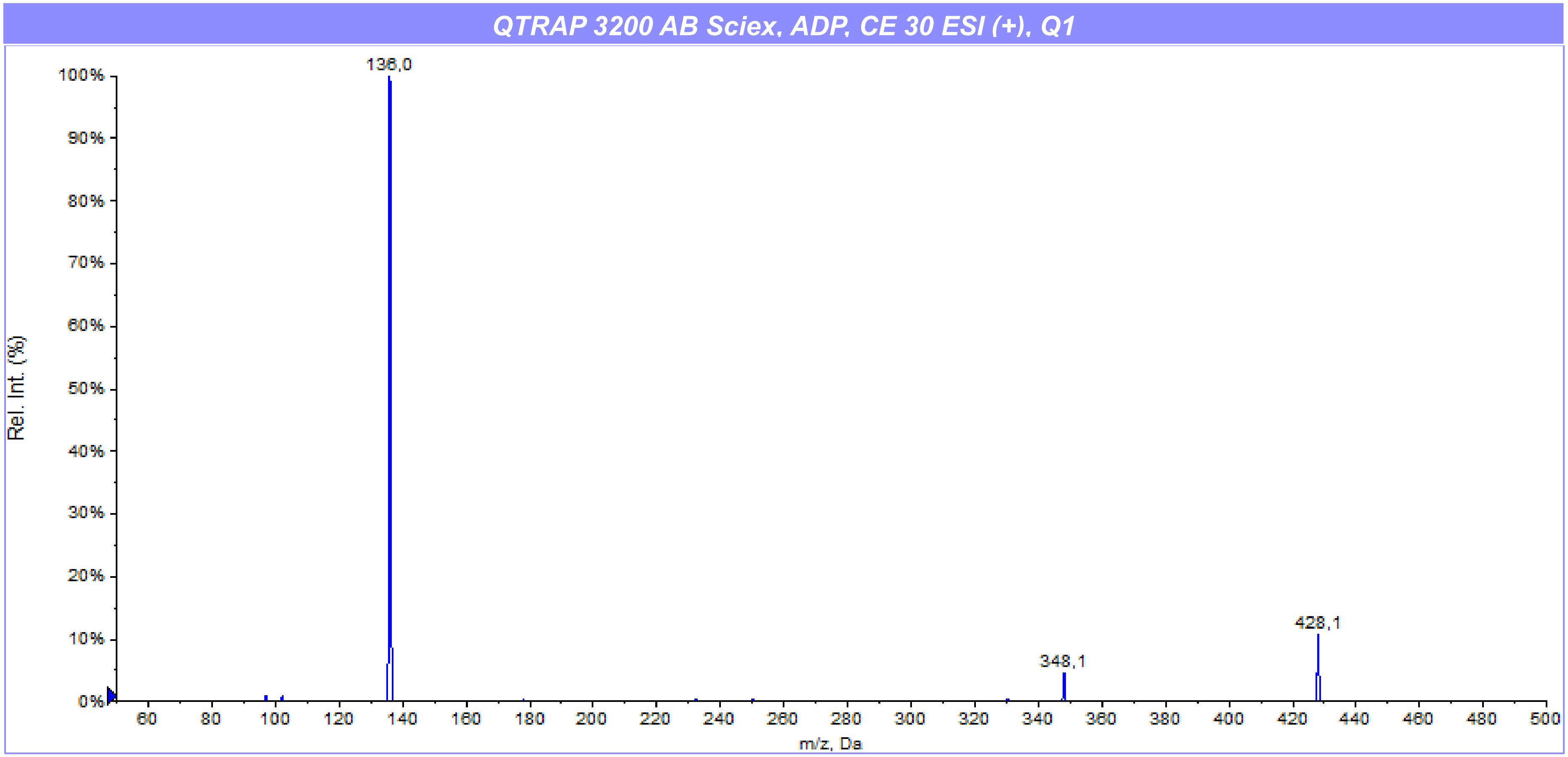
QTRAP 3200 AB Sciex, ADP, CE 30 ESI (+), Q1
Rel. Int. (%)
100%
90%
80%
70%
60%
50%
40%
30%
20%
10%
0%
136,0
348,1
428,1
60
80
100
120
140
160
180
200
220
240
260
280
300
320
340
360
380
400
420
440
460
480
500
m/z, Da

QTRAP 3200 AB Sciex. ADP. CE 30 ESI (+). EPI

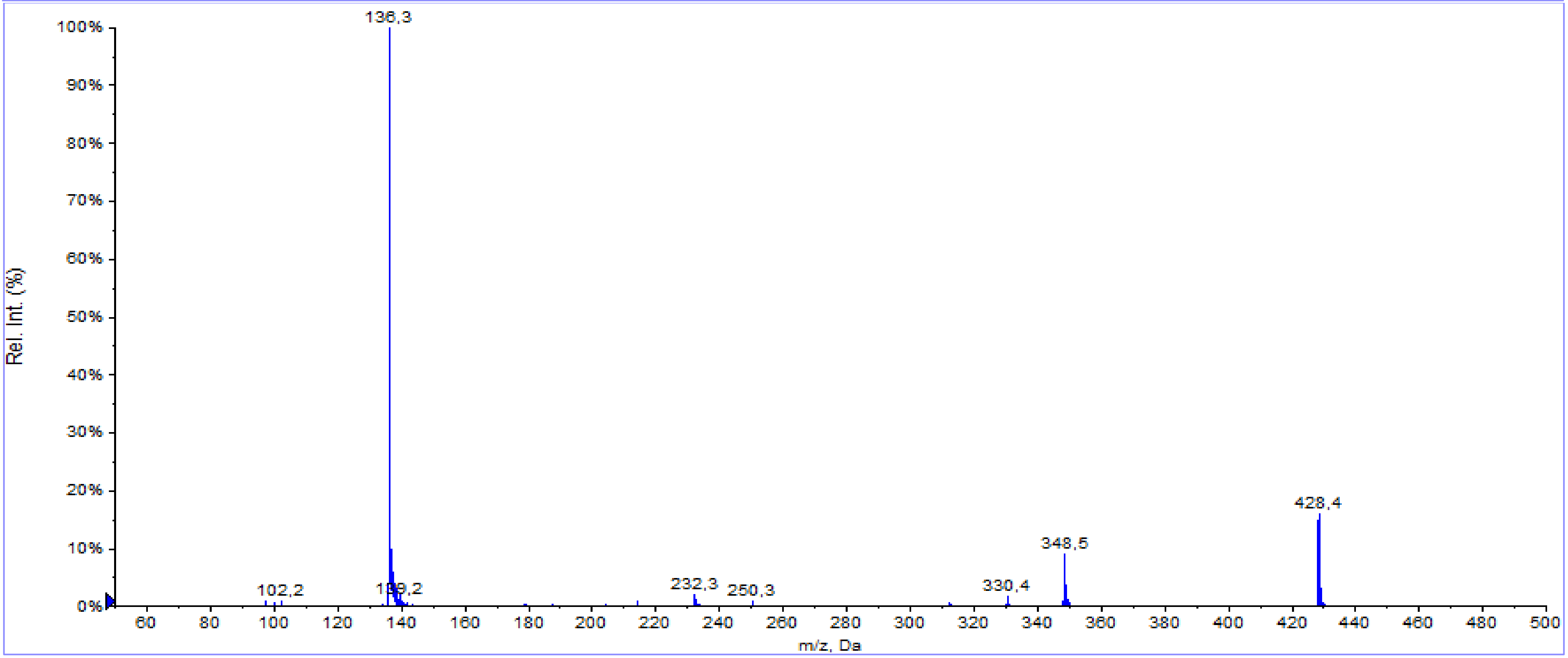

API 3200 AB Sciex, ADP, CE 30 ESI (+)

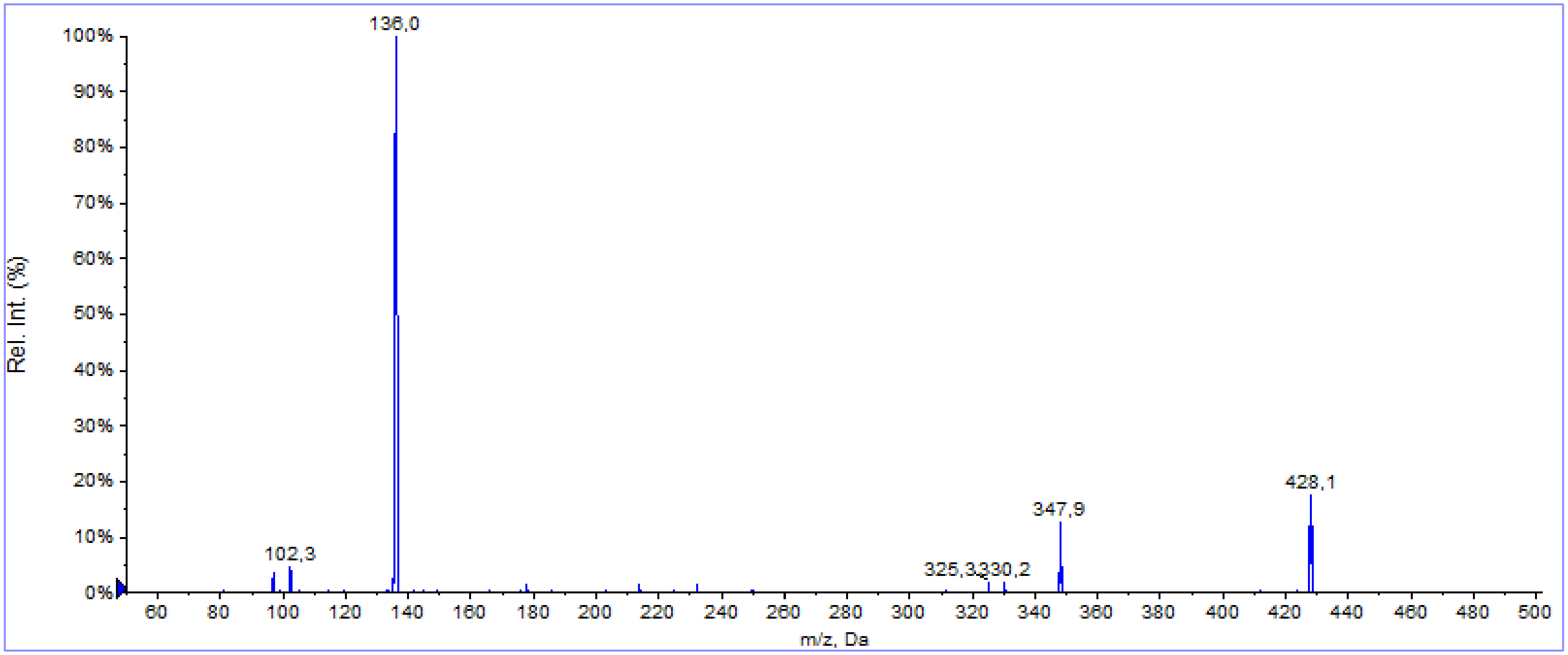

QExactive Thermo, AMP, CE 20 ESI (-)

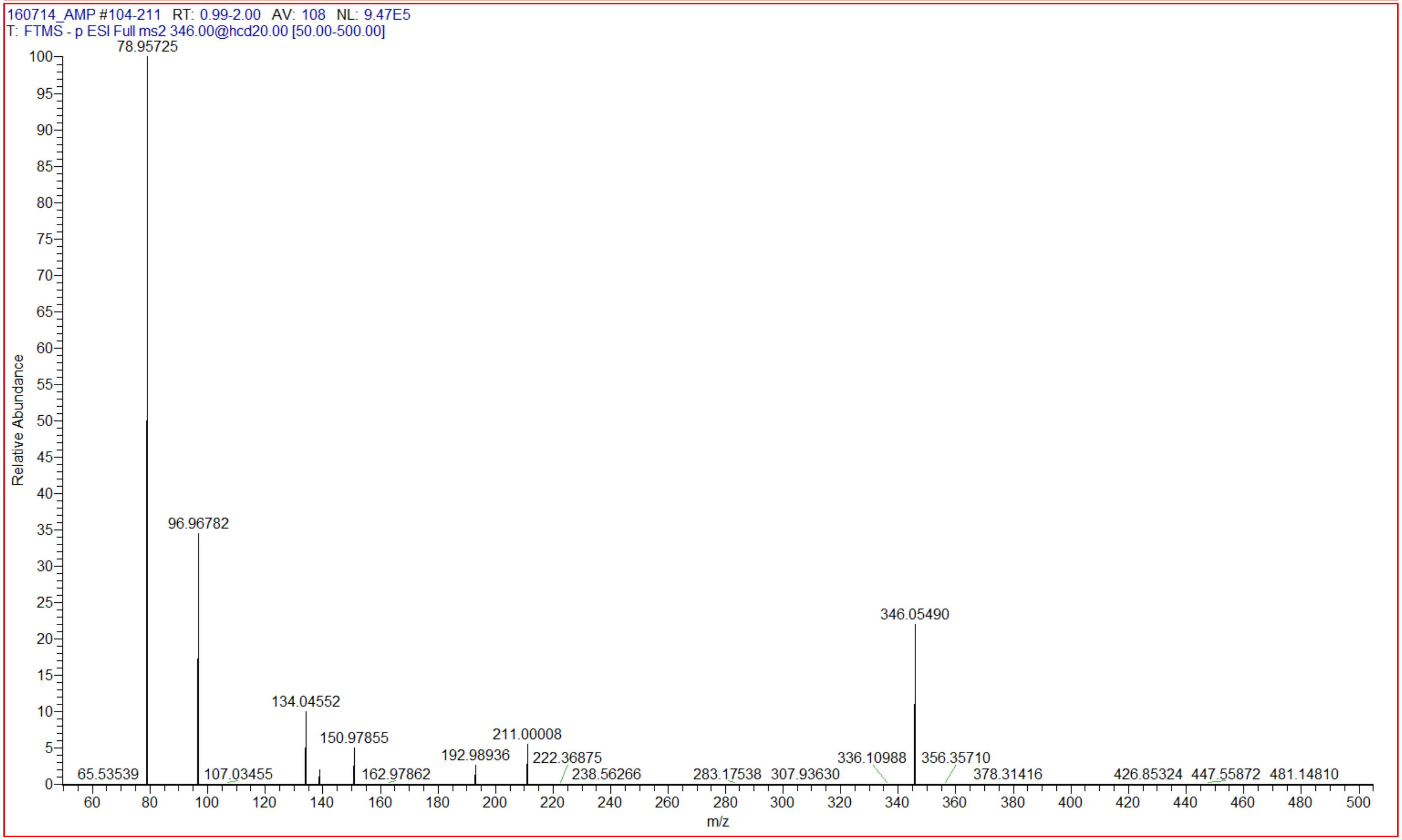

QTOF Waters, AMP, CE 20 ESI (-)

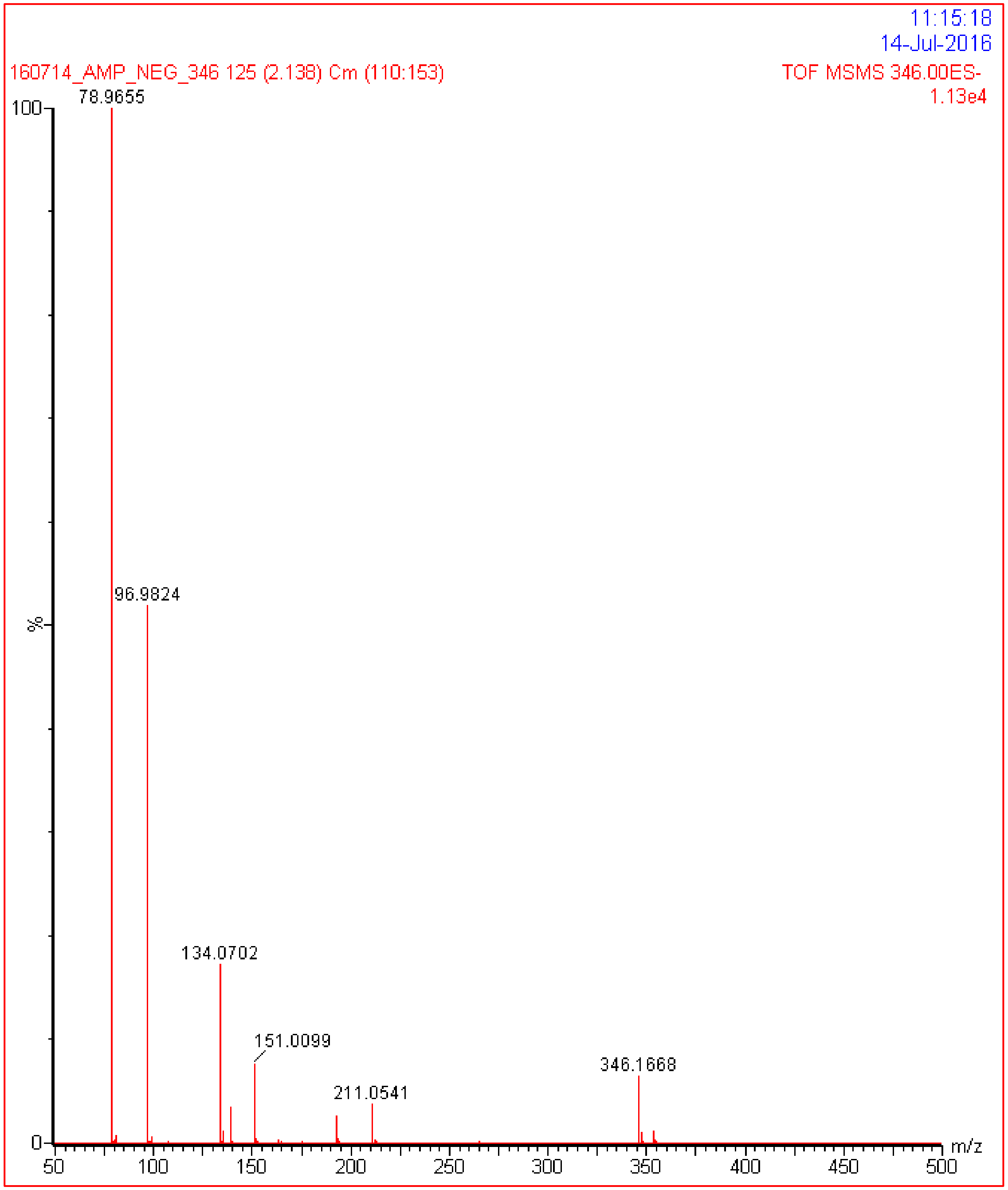

QTRAP 3200AB Sciex, AMP, CE 39 ESI (-), Q1

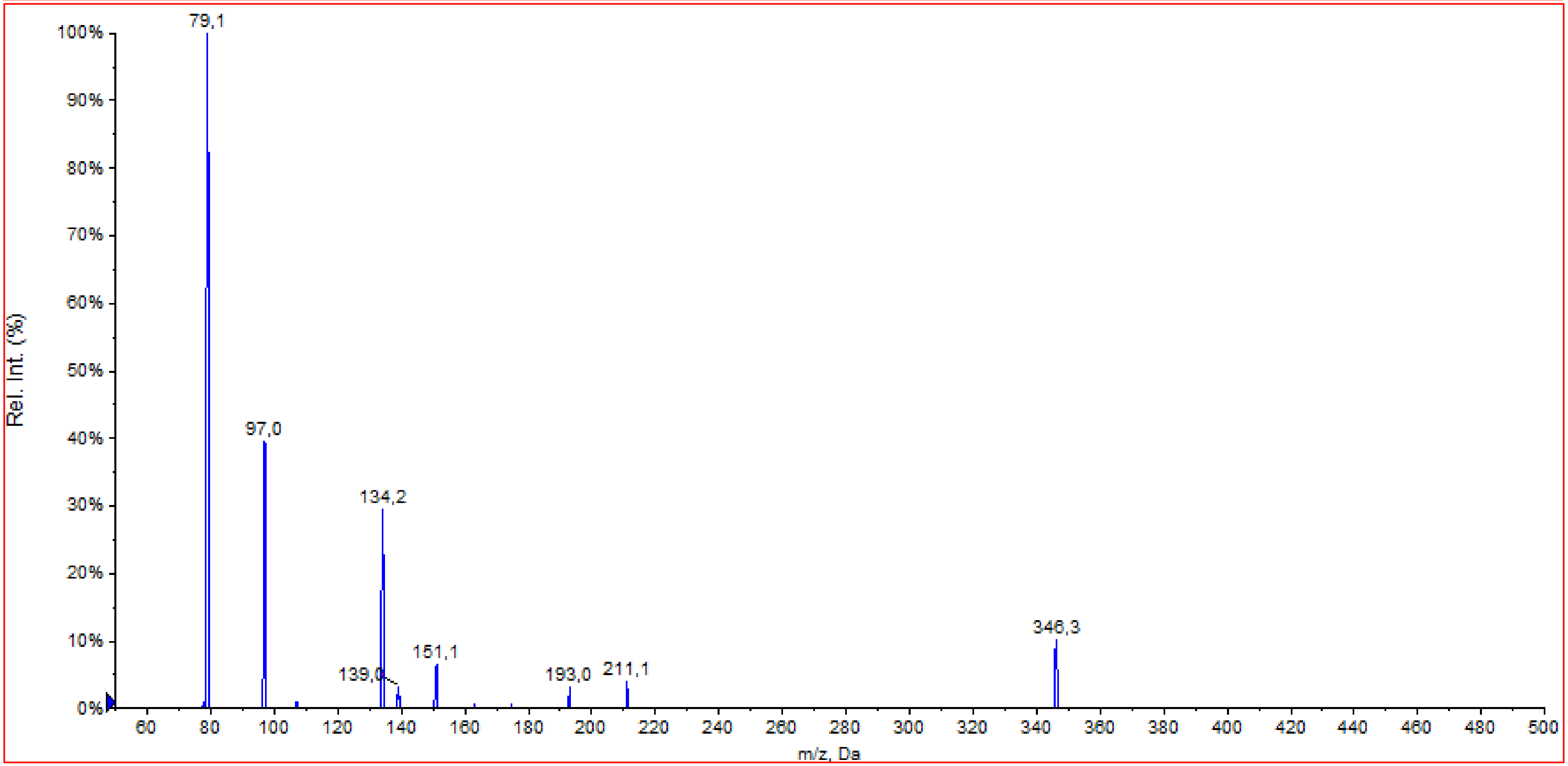

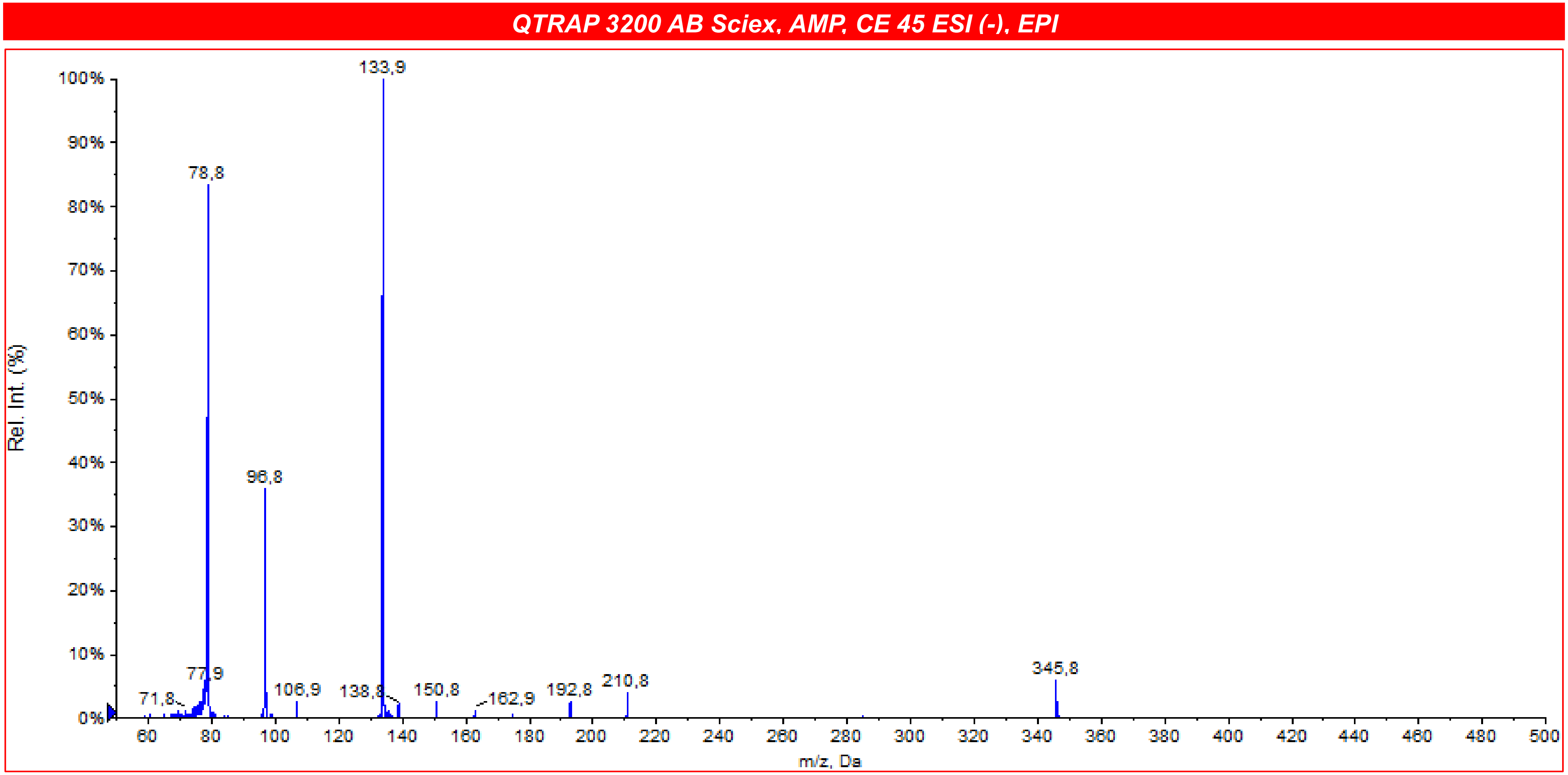
QTRAP 3200 AB Sciex, AMP, CE 45 ESI (-), EPI
Rel. Int. (%)
100%
90%
80%
70%
60%
50%
40%
30%
20%
10%
0%
133,9
78,8
96,8
77,9
71,8
106,9
138,8
150,8
162,9
192,8
210,8
345,8
60
80
100
120
140
160
180
200
220
240
260
280
300
320
340
360
380
400
420
440
460
480
500
m/z, Da

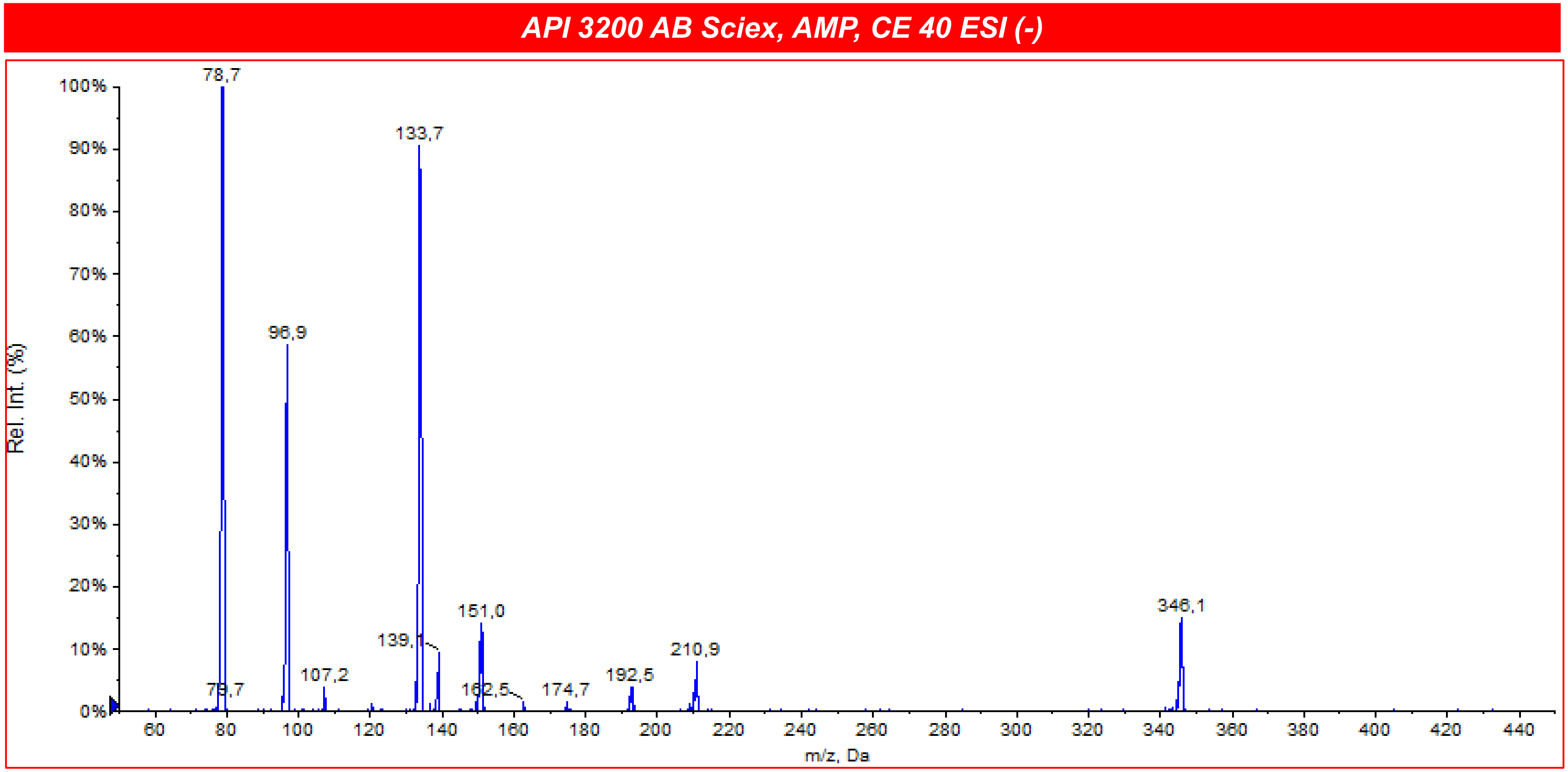
API 3200 AB Sciex, AMP, CE 40 ESI (-)
Rel. Int. (%)
100%
90%
80%
70%
60%
50%
40%
30%
20%
10%
0%
78,7
96,9
133,7
151,0
139,1
346,1
210,9
107,2
192,5
79,7
162,5
174,7
60
80
100
120
140
160
180
200
220
240
260
280
300
320
340
360
380
400
420
440
m/z, Da

QExactive Thermo, AMP, CE 15 ESI (+)

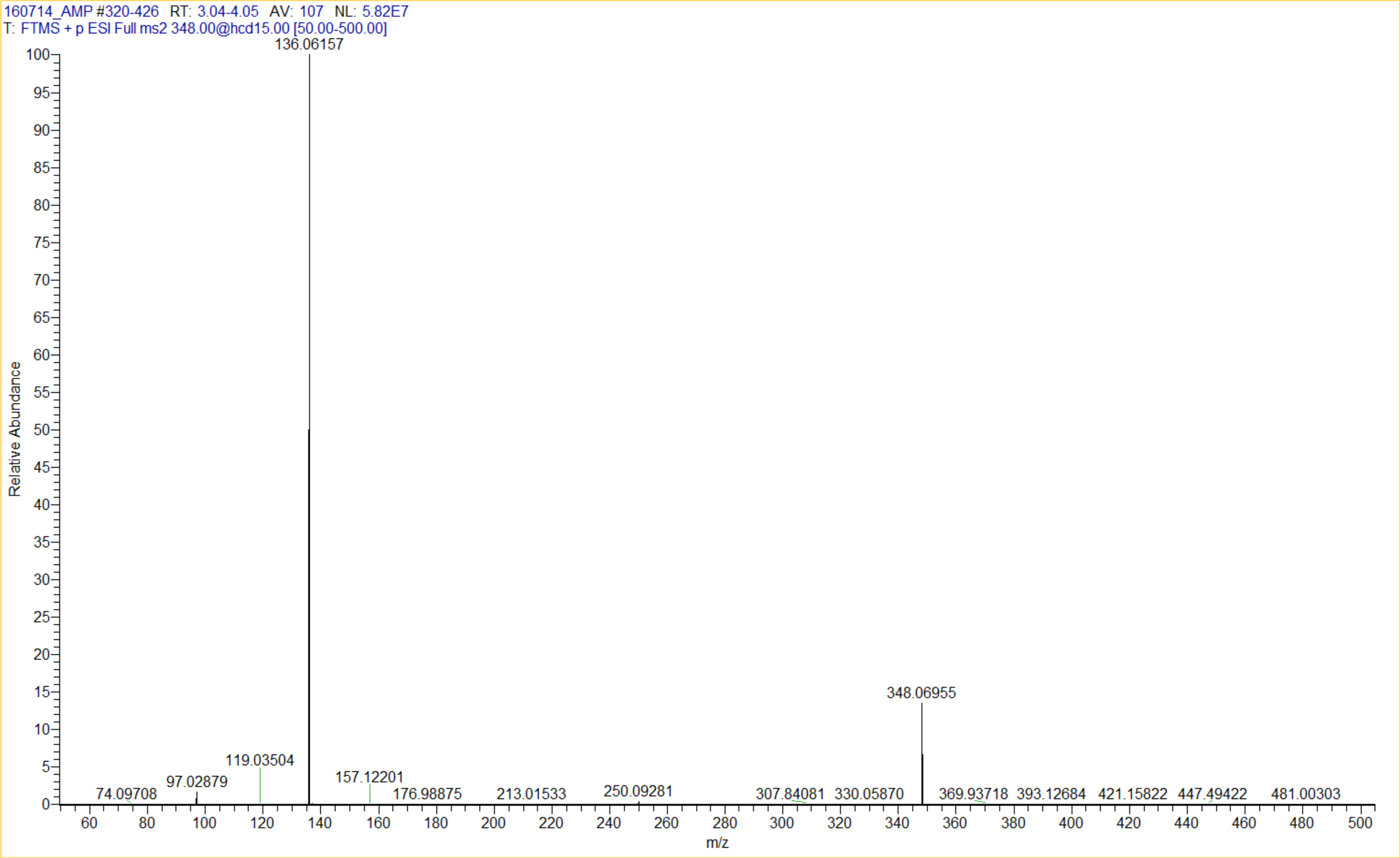

QTOF Waters, AMP, CE 12 ESI (+)

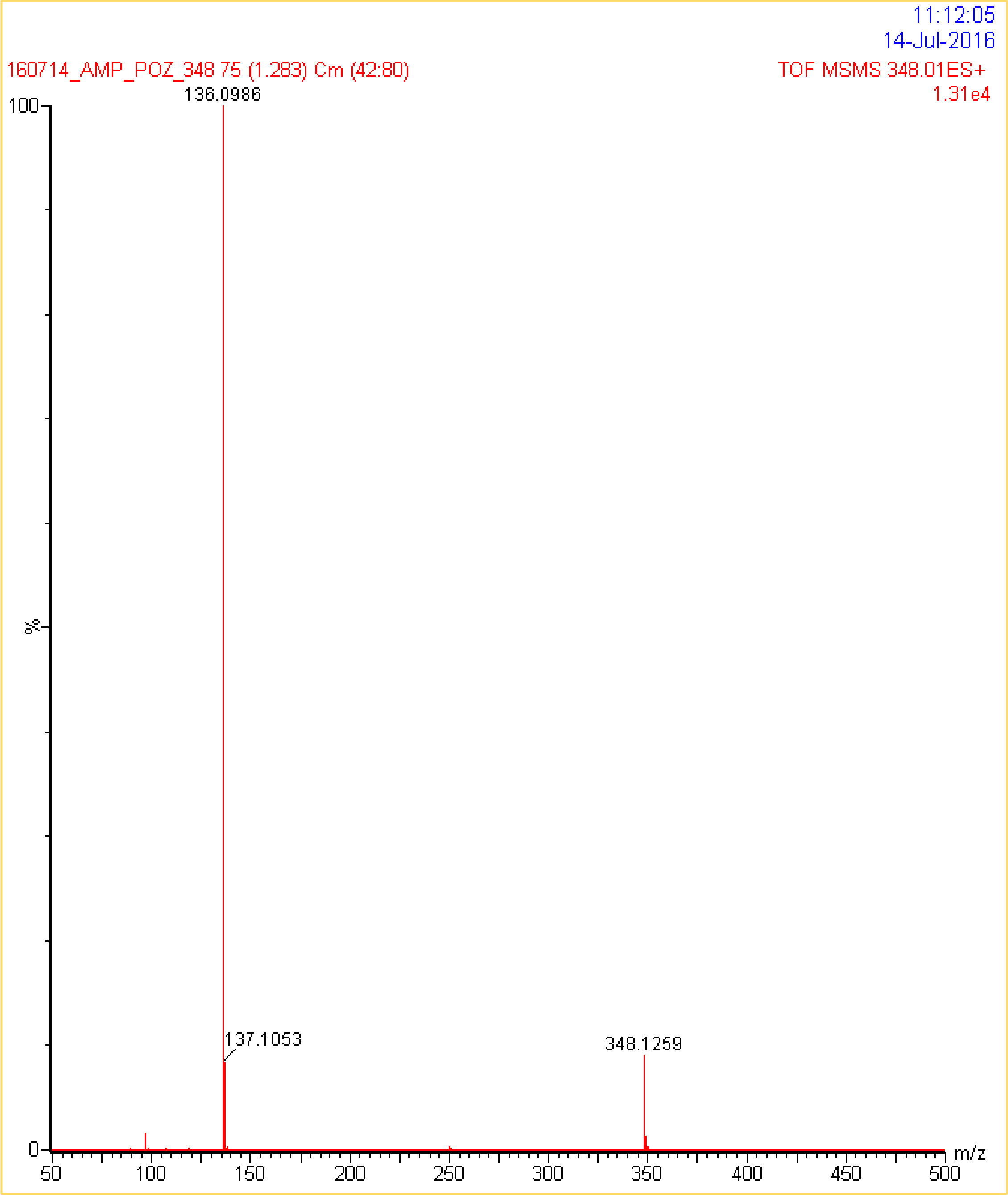

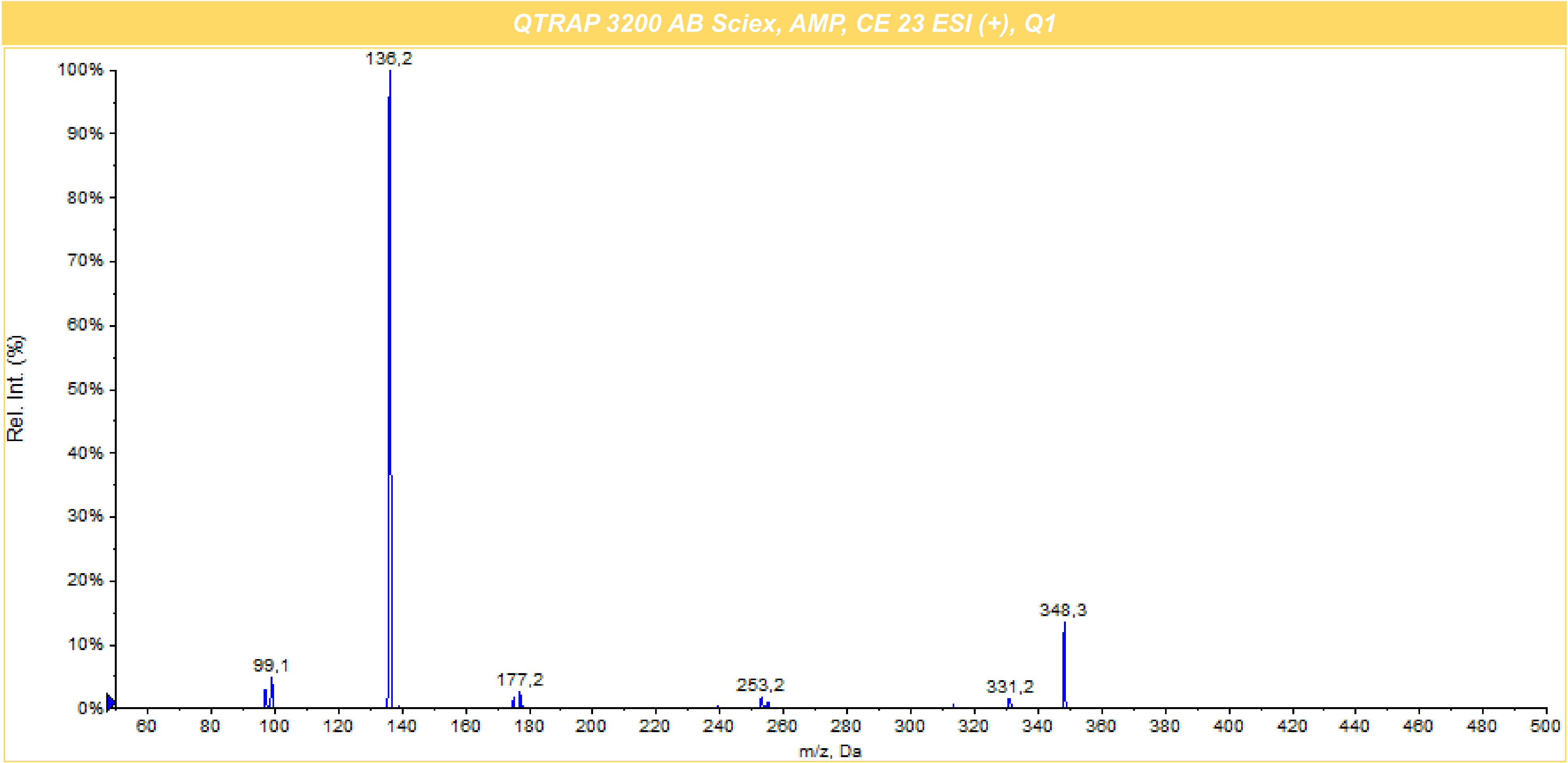
QTRAP 3200 AB Sciex, AMP, CE 23 ESI (+), Q1
Rel. Int. (%)
m/z, Da
136,2
99,1
177,2
253,2
331,2
348,3

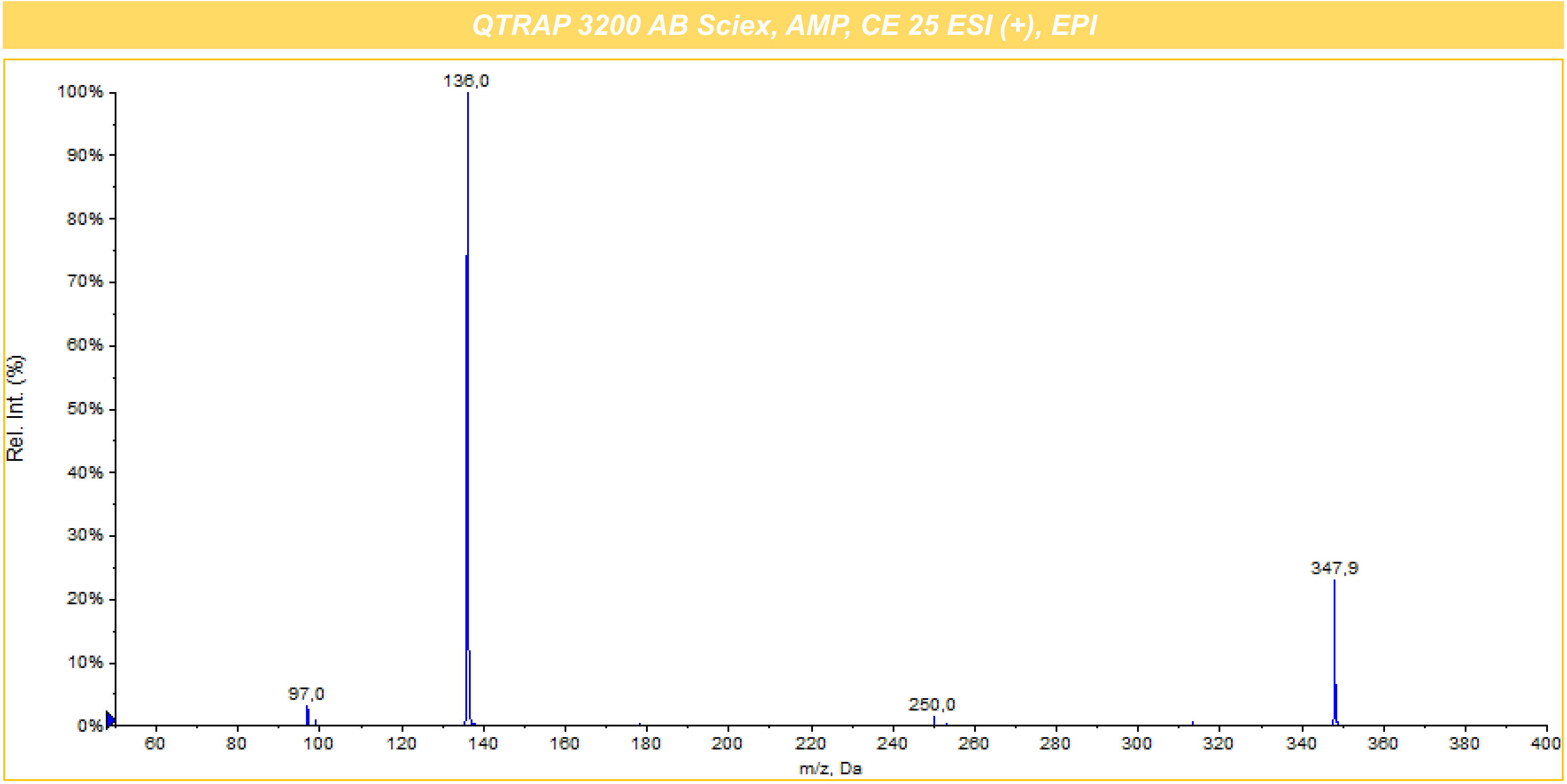

QTRAP 3200 AB Sciex, AMP, CE 25 ESI (+), EPI
Rel. Int. (%)
100%
90%
80%
70%
60%
50%
40%
30%
20%
10%
0%
136,0
97,0
250,0
347,9
60
80
100
120
140
160
180
200
220
240
260
280
300
320
340
360
380
400
m/z, Da

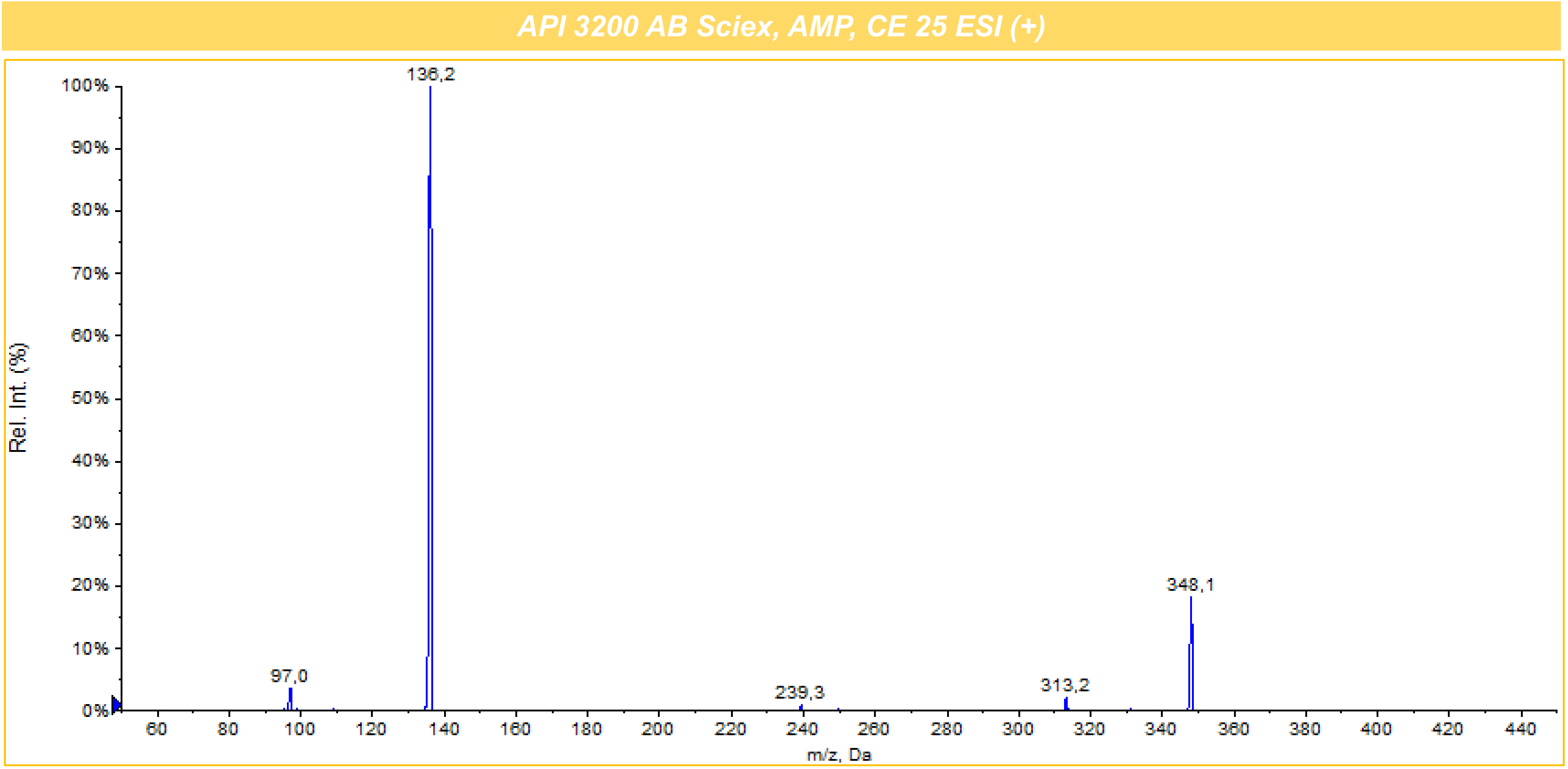
API 3200 AB Sciex, AMP, CE 25 ESI (+)
Rel. Int. (%)
100%
90%
80%
70%
60%
50%
40%
30%
20%
10%
0%
136,2
97,0
239,3
313,2
348,1
60
80
100
120
140
160
180
200
220
240
260
280
300
320
340
360
380
400
420
440
m/z, Da

References

1 Lin, L. *et al.* Facile synthesis of nucleoside 5 '-(alpha-P-seleno)-triphosphates and phosphoroselenoate RNA transcription. *RNA* **17**, 1932-1938, doi:10.1261/rna.2719311 (2011).

2 Amir, A. *et al.* Nucleoside-5 '-phosphorothioate analogues are biocompatible antioxidants dissolving efficiently amyloid beta-metal ion aggregates. *Dalton Transactions* **41**, 8539-8549, doi:10.1039/c2dt30631j (2012).

3 Haas, M., Ginsburg-Shmuel, T., Fischer, B. & Reiser, G. 5-OMe-uridine-5 '-O-(alpha-boranodiphosphate), a novel nucleotide derivative highly active at the human P2Y(6) receptor protects against death-receptor mediated glial apoptosis. *Neuroscience Letters* **578**, 80-84, doi:10.1016/j.neulet.2014.06.030 (2014).

4 Guranowski, A. *et al.* Fhit proteins can also recognize substrates other than dinucleoside polyphosphates. *Febs Letters* **582**, 3152-3158, doi:10.1016/j.febslet.2008.07.060 (2008).

5 Kirschberg, T. *et al.* Synthesis and characterization of 1 '-C-cyano-2 '-fluoro-2 '-C-methyl pyrimidine nucleosides as HCV polymerase inhibitors. *Bioorganic & Medicinal Chemistry Letters* **25**, 1040-1043, doi:10.1016/j.bmcl.2015.01.021 (2015).

6 Schelle, M. & Bertozzi, C. Sulfate metabolism in mycobacteria. *Chembiochem* **7**, 1516-1524, doi:10.1002/cbic.200600224 (2006).

7 Ghosh, B. *et al.* Nontoxic Chemical, Interdiction of the Epithelial-to-Mesenchymal Transition by Targeting Cap-Dependent Translation. *ACS Chemical Biology* **4**, 367-377, doi:10.1021/cb9000475 (2009).

8 Li, S. *et al.* Treatment of Breast and Lung Cancer Cells with a N-7 Benzyl Guanosine Monophosphate Tryptamine Phosphoramidate Pronucleotide (4Ei-1) Results in Chemosensitization to Gemcitabine and Induced eIF4E Proteasomal Degradation. *Molecular Pharmaceutics* **10**, 523-531, doi:10.1021/mp300699d (2013).

9 Wang, L. *et al.* Phosphorothioation of DNA in bacteria by dnd genes. *Nature Chemical Biology* **3**, 709-710, doi:10.1038/nchembio.2007.39 (2007).

10 Gollnest, T. *et al.* Membrane-permeable Triphosphate Prodrugs of Nucleoside Analogues. *Angewandte Chemie-International Edition* **55**, 5255-5258, doi:10.1002/anie.201511808 (2016).

11 Auriola, S., Frith, J., Rogers, M., Koivuniemi, A. & Monkkonen, J. Identification of adenine nucleotide-containing metabolites of bisphosphonate drugs using ion-pair liquid chromatography-electrospray mass spectrometry. *Journal of Chromatography B* **704**, 187-195, doi:10.1016/S0378-4347(97)00490-8 (1997).

12 Rogers, M., Russell, R., Blackburn, G., Williamson, M. & Watts, D. Metabolism of halogenated bisphosphonates by the cellular slime-mold dictyostelium-discoideum. *Biochemical and Biophysical Research Communications* **189**, 414-423, doi:10.1016/0006-291X(92)91574-A (1992).

13 Frith, J., Monkkonen, J., Blackburn, G., Russell, R. & Rogers, M. Clodronate and liposome-encapsulated clodronate are metabolized to a toxic ATP analog, adenosine 5'-(beta,gamma-dichloromethylene) triphosphate, by mammalian cells in vitro. *Journal of Bone and Mineral Research* **12**, 1358-1367, doi:10.1359/jbmr.1997.12.9.1358 (1997).
